# Group-based trajectory models of integrated vaccine delivery and equity in low- and middle-income countries

**DOI:** 10.1186/s12939-023-02088-x

**Published:** 2024-01-09

**Authors:** Sanjana J. Ravi, Andrés I. Vecino-Ortiz, Christina M. Potter, Maria W. Merritt, Bryan N. Patenaude

**Affiliations:** 1 Center for Health Security, Johns Hopkins Bloomberg School of Public Health, 700 East Pratt Street, Suite 900, Baltimore, MD 21202 USA; 2 Department of International Health, Johns Hopkins Bloomberg School of Public Health, 615 North Wolfe Street, Suite E8527, Baltimore, MD 21205 USA; 3 Berman Institute of Bioethics, Johns Hopkins Bloomberg School of Public Health, 1809 Ashland Avenue, Baltimore, MD 21205 USA; 4 International Vaccine Access Center, Johns Hopkins Bloomberg School of Public Health, 415 North Washington Street, 5th Floor, Baltimore, MD 21231 USA

## Abstract

**Background:**

Integrated vaccine delivery – the linkage of routine vaccination with provision of other essential health services – is a hallmark of robust primary care systems that has been linked to equitable improvements in population health outcomes.

**Methods:**

We gathered longitudinal data relating to routine immunization coverage and vaccination equity in 78 low- and middle-income countries that have ever received support from Gavi, the Vaccine Alliance, using multiple imputation to handle missing values. We then estimated several group-based trajectory models to describe the relationship between integrated vaccine delivery and vaccination equity in these countries. Finally, we used multinomial logistic regression to identify predictors of group membership.

**Results:**

We identified five distinct trajectories of geographic vaccination equity across both the imputed and non-imputed datasets, along with two and four trajectories of socioeconomic vaccination equity in the imputed and non-imputed datasets, respectively. Integration was associated with reductions in the slope index of inequality of measles vaccination in the countries analyzed. Integration was also associated with an increase in the percentage of districts reporting high measles vaccination coverage.

**Conclusions:**

Integrated vaccine delivery is most strongly associated with improvements in vaccination equity in settings with high baseline levels of inequity. Continued scholarship is needed to further characterize the relationship between integration and health equity, as well as to improve measurement of vaccination coverage and integration.

**Supplementary Information:**

The online version contains supplementary material available at 10.1186/s12939-023-02088-x.

## Background

Health systems research features a longstanding debate over the merits of vertical versus horizontal modes of health service delivery. Broadly, vertical programs are disease-specific, often freestanding initiatives with specified objectives to be achieved within a limited timeframe [1]. The Global Polio Eradication Initiative – a USD$20 billion program that has eliminated poliomyelitis incidence by 99.9% since its inception in 1988 – is a classic example of a vertical health program, as was the recent global COVID-19 vaccination effort [2]. By contrast, the World Health Organization (WHO) defines horizontal (i.e., “integrated”) approaches as “the process of bringing together common functions within and between organizations to solve common problems, developing a commitment to shared vision and goals and using common technologies and resources to achieve these goals” [3]. Systems for delivering comprehensive primary health care – including all preventive, curative, palliative, and rehabilitative services needed over a person’s lifetime – embody the ethos of integrated health service provision, which aims to “meet people’s health needs throughout their lives” [4].

The prevalence of vertical programs in resource-constrained settings raises questions about conditions under which it is appropriate to transition a given vertical program into an integrated, horizontal system – and, if appropriate, how best to facilitate this transition. A robust body of literature affirms the value of pursuing integrated approaches to delivering many routine health services, citing improvements in health system governance, program sustainability, community involvement, equitable provision of care, and access to and coverage of essential services [5–9]. Other studies, however, paint a murkier picture: several systematic reviews, for example, assert that the purported benefits of integration are highly variable across contexts or remain largely unproven in public health and healthcare practice, citing logistical challenges and unequal resource allocation as barriers to achieving desired levels of coverage [10–13]. Furthermore, some studies note that integration may risk undermining existing health services, including immunization, and that the dearth of quality evidence on the benefits and drawbacks of integration makes it difficult to accurately assess its true impacts [11, 14, 15].

Though integrated delivery may not be appropriate for every health service, integrating routine vaccinations with other core health services may yield positive benefits for population health. Because immunization coverage is relatively high in many countries, vaccination programs are an attractive vehicle for concomitantly increasing coverage of other critical health services. For this reason, WHO’s Immunization Agenda 2030 (IA2030) explicitly emphasizes the importance of “building a strong national immunization infrastructure integrated into primary health care services, as a way to both achieve and sustain elimination and eradication goals,” as well as to ensure that all people benefit from immunization throughout the life-course [16]. For the purposes of this investigation, we extrapolate from this description to define integrated vaccine delivery as the linkage of routine vaccination services with other core public health interventions within a primary healthcare system (versus standalone vertical programs), in alignment with Gavi’s description of integrated delivery [17].

Some evidence suggests that linking immunization with other public health interventions and core health services (e.g., deworming, Vitamin A supplementation, bednet distribution) could reduce vaccination inequities in low- and middle-income countries (LMICs) [14, 18–21]. For this reason, countries receiving financial and programmatic support from Gavi, the Vaccine Alliance (“Gavi”) have implemented strategies aimed at both improving health equity and strengthening health systems [22]. WHO further notes that “integrated health services by design enhance equity; they encourage the selection of services based on the holistic needs of a given population and deliver many different types of care across the life course, from health protection and promotion and disease prevention to diagnosis, treatment, disease management, long-term care, rehabilitation and palliative care” [23].

Despite the documented benefits and drawbacks of integrated vaccine delivery, its relationship with vaccination equity has not, to our knowledge, been systematically studied across LMICs. Moreover, we have not identified any studies measuring the longitudinal impacts of integrated vaccine delivery on vaccination equity at the country level. Here, we examine the relationship between integrated vaccine delivery and vaccination equity in low- and middle-income countries by using longitudinal data to develop several group-based trajectory models.

## Methods

### Measuring integrated vaccine delivery & vaccination equity

First, we purposively examined the peer-reviewed and grey literature for existing measures of vaccination equity and integration. We found that studies of vaccination distribution and uptake in LMICs generally frame equity in terms of crude coverage within and across key dimensions of vulnerability, including but not limited to age, sex or gender, race, wealth level, education level, citizenship status, and geography (i.e., urban vs. rural setting) [24–29]. Crude coverage refers to the proportion of individuals within a population targeted for vaccination that actually receives said vaccination (by contrast with effective coverage, which describes the proportion of a target population that receives a given vaccination and subsequently undergoes seroconversion, thereby developing protective immunity) [30].

The crude coverage-based approach to measuring vaccination equity has been embraced by many public health practitioners, donors, and decision-makers, including Gavi. As part of its 2016-2020 strategy to support equitable immunization programs in lower-income countries, Gavi published a set of accompanying indicators to monitor progress toward its stated vaccine, systems, sustainability, and market-shaping goals.[17] Two equity measures described in the strategy include equity of vaccination coverage by geography (i.e., the proportion of districts with coverage of the third dose of diphtheria-pertussis-tetanus-containing vaccine [DTP3] ≥80%, across all Gavi countries, hereinafter referred to as “geographic equity”) and equity of coverage by poverty status (i.e., the difference in coverage of the third dose of pentavalent vaccine between the richest and poorest quintiles, hereinafter referred to as “socioeconomic equity”) [17].

Arsenault et al. note that measuring absolute and relative coverage gaps between the wealthiest and poorest quintiles – the difference in coverage and the ratio of coverage, respectively – is an intuitive approach to quantifying vaccination equity when only two subgroups of analysis are under consideration (e.g., urban vs. rural, male vs. female) [31]. However, applying this approach to poverty status could conceal important coverage disparities within and between mid-range wealth quintiles [31].

For this reason, we modified Gavi’s measures of geographic and socioeconomic equity to examine, respectively, the proportion of districts within a given country to achieve ≥80% crude coverage of the first dose of measles-containing vaccine (MCV1) and the slope index of inequality (SII) of measles vaccination. WHO defines SII as “a complex, weighted measure of inequality that represents the absolute difference in estimated values of a health indicator between the most-advantaged and most disadvantaged (or vice versa for adverse health outcome indicators), while taking into consideration all the other subgroups” [32]. In this analysis, we calculated SII from estimates of MCV1 coverage disaggregated by wealth quintile. Larger, positive SII values indicate that high MCV1 coverage is more prevalent among wealthier quintiles, while smaller, negative values reflect greater coverage in poorer quintiles. This measure enables consideration of MCV1 coverage in mid-range wealth quintiles that would otherwise be excluded by using the absolute difference in coverage or the ratio of coverage.

We chose to frame equity in terms of MCV1 rather than DTP3 or pentavalent vaccination coverage for several reasons. First, DTP doses are administered almost exclusively through routine health programs, whereas measles vaccinations are delivered through both horizontal and vertical pathways, even in settings with high coverage [33]. Thus, considering MCV1 coverage enables us to discern whether equitable coverage varies by modality of vaccine delivery. Second, in many countries, the first three doses of DTP vaccines are typically administered at two, four, and six months of life, respectively (or at 6 weeks, 10 weeks, and 14 weeks of life if using the pentavalent vaccine) [34, 35]. MCV1, however, is not administered until at least 9 months of life to prevent maternal antibody interference with the live vaccine and subsequent vaccination failure [36]. Due to patient attrition often observed between DTP3 and MCV1 administration in resource-constrained settings, achieving and sustaining high MCV1 coverage thus represents a stretch goal for health systems in LMICs [37, 38]. Finally, measles outbreaks function as proverbial “canaries in a coalmine,” signaling poor health system functioning, persistent inequities in coverage, and challenges in immunization program implementation [37, 39–42]. Measles is also among the most contagious diseases, infecting and killing tens of thousands of children each year and leaving survivors with permanently weakened immune systems [43, 44]. Thus, we felt that framing our measures in terms of MCV1 coverage would serve as more meaningful indicators of vaccination equity in LMICs.

As part of its 2016-2020 strategy, Gavi utilized a measure of integrated vaccine delivery developed by WHO’s SAGE Decade of Vaccines Working Group (“the Working Group”). We chose to adopt this measure, which considers crude coverage of four core health services: DTP3, MCV1, protection at birth against neonatal tetanus (PAB), and at least one antenatal care visit (ANC1). If national co-coverage levels of these services are within ten percentage points of one another, and all four are greater than or equal to 70%, then the country in question is considered to have achieved integrated vaccine delivery. Per the Working Group, weak coverage correlation between these four services indicates poor integration, while the 70% threshold excludes weak health systems with poor service coverage across the board from being considered integrated [17]. This measure of integrated vaccine delivery is no longer a part of Gavi’s updated 2021-2025 strategy, but was in effect during the years in which our data was collected.

### Data collection

From our purposive literature review, we identified several studies by Arsenault et al. that characterized country-level predictors of vaccination equity in countries supported by Gavi [31, 45, 46]. Drawing from these studies, we determined which variables to gather for our investigation. Next, we designed a collation tool in Google Forms, which we used to compile a dataset of these variables, which relate to routine health service coverage, socioeconomic conditions, and additional measures of health system performance for 78 countries that had ever received Gavi support, focusing on the years 2003-2019 (i.e., the period for which longitudinal routine immunization data from WHO were available). We excluded data from 2020 to avoid confounding effects on vaccine delivery due to the COVID-19 pandemic. We also excluded the years preceding 2008 in our geographic equity analysis due to unreliable district-level coverage estimates reported during those years. The complete dataset is available in Appendix A, and a glossary of collected indicators and their sources is available in Appendix B.

### Group-based trajectory modeling

We applied group-based trajectory modeling (GBTM) to describe developmental trajectories of geographic and socioeconomic vaccination equity in Gavi countries between 2003 and 2019. GBTM was pioneered by Daniel S. Nagin and Kenneth C. Land, who first described the method in a landmark paper examining the relationships between age, rates of criminality, and differences between chronic and less-active criminal offenders [47]. GBTM has since been applied across a wide range of studies in psychology, sociology, criminology, and medicine [48–51]. GBTM is a method for approximating distinct developmental trajectories of an outcome of interest, drawing from longitudinal data. It has been described as a form of latent class modeling, wherein a set of observed variables are related to a set of latent variables (i.e., variables that are not directly observed, but inferred from observed variables) [52]. GBTM is a useful method for characterizing distinct longitudinal trends in a given outcome of interest, as well as for describing phenomena like vaccination equity that may not follow a predictable trajectory [53–55].

### Using GBTM to explore integration and equity

As a form of latent class analysis, GBTM lends itself to analyzing the relationship between integrated vaccine delivery and equity for several reasons. First, observable variables – such as immunization coverage, wealth, and population – are often prone to measurement error and method variance, and latent variable methods can minimize potential systematic bias stemming from a single variable [56]. Second, given significant heterogeneity within countries and the fact that countries develop and achieve equitable health outcomes at differing rates, classifying them based solely on observable characteristics measured at the national level may not adequately explain longitudinal trends in equity. For example, though income level is strongly associated with vaccination equity in LMICs, some countries (e.g., Nigeria, a lower-middle-income country) nevertheless report significantly lower levels of MCV1 coverage across wealth quintiles compared to their peers (e.g., Zambia, another lower-middle-income country) and in some cases, even their poorer counterparts (e.g., Malawi, a low-income country) [57, 58]. Examining membership in unobserved classes could elucidate unexpected patterns in vaccination equity and help relate these patterns to observable variables [59].

Finally, both integration and equity manifest heterogeneously across countries. For example, infrastructural improvements may initially spark rapid increases in vaccination coverage, but these increases often plateau once coverage surpasses 80% [60]. Health service integration is also a highly dynamic phenomenon that may have varying effects on equity in different settings. Thus, given the possibility of diminishing equity returns in settings with already-high coverage and the heterogeneous effects associated with different forms of health service integration, grouping countries by their respective developmental equity trajectories may serve as a useful comparative device.

To the best of our knowledge, GBTM has not been previously applied in peer-reviewed health systems research focusing on vaccination, equity, or LMICs, though the method has been used to examine country-level phenomena such as infant mortality and terrorism [61, 62]. We posit that GBTM could help chart the developmental trajectories of health system performance over time, thereby elucidating how health outcomes of interest (e.g., equitable vaccination coverage) evolve longitudinally. It may also help identify correlates of equity and facilitate comparison between high- and low-performing groups.

### Model specification, diagnostics, and data analysis

All data analysis was performed using Stata 17 [63]. We used *traj*, a Stata plugin developed by Bobby Jones and Daniel Nagin, to estimate several group-based trajectory models of geographic and socioeconomic vaccination equity across the 78 countries in our sample [64]. We also used *siilin*, a command developed by the International Center for Equity in Health, to estimate SII for countries with publicly available MCV1 coverage estimates disaggregated by wealth quintile [65]. Accompanying Stata code and output for each analysis is provided in Appendices C-F.

We first performed multivariate normal imputation to handle missing data in our sample, given a high degree of missingness in the variables required to measure integration (i.e., ANC1 and PAB), as well as those required to measure our equity outcomes (i.e., geographic equity, SII, and MCV1 coverage across each of the five wealth quintiles). We performed 10 imputations across these 9 variables for all 78 countries. This created an imputed dataset of 14,586 observations, of which 2,140 were ultimately used in the imputation regression model (see Table 1 for a summary of dropped observations). The analyses described hereinafter were conducted separately on both the imputed and non-imputed datasets. We chose to analyze both datasets to assess the extent to which our findings were sensitive to imputation.

**Table 1:** Observations omitted from the multiple imputation regression model

Country	Year									Total
	2003	2004	2005	2006	2007	2008	2009	2010	2011	
Afghanistan	11	11	11	11	11	11	11	11	1	167
Albania	11	11	11	11	11	11	1	11	11	167
Angola	11	11	11	11	11	11	11	11	11	177
Armenia	11	11	1	11	11	11	11	1	11	167
Azerbaijan	11	11	11	1	11	11	11	11	11	177
Bangladesh	11	1	11	1	1	11	11	11	1	127
Benin	11	11	11	1	11	11	11	11	11	147
Bhutan	11	11	11	11	11	11	11	11	11	187
Bolivia	1	11	11	11	11	1	11	11	11	167
Bosnia & Herzegovina	11	11	11	1	11	11	11	11	11	167
Burkina Faso	1	11	11	1	11	11	11	1	11	157
Burundi	11	11	1	11	11	11	11	1	11	157
Cambodia	11	11	1	11	11	11	11	1	11	157
Cameroon	11	1	11	11	11	11	11	11	1	147
Central African Rep..	11	11	11	1	11	11	11	1	11	157
Chad	11	1	11	11	11	11	11	1	11	147
China	11	11	11	11	11	11	11	11	11	187
Comoros	11	11	11	11	11	11	11	11	11	177
Cuba	11	11	11	11	11	11	11	11	11	187
Democratic People's Republic of Korea	11	11	11	11	11	11	11	11	11	177
Democratic Republic of the Congo	11	11	11	11	1	11	11	1	11	147
Djibouti	11	11	11	11	11	11	11	11	11	187
Eritrea	11	11	11	11	11	11	11	11	11	187
Ethiopia	11	11	1	11	11	11	11	11	1	147
Gambia	11	11	11	1	11	11	11	1	11	147
Georgia	11	11	11	11	11	11	11	11	11	187
Ghana	1	11	11	1	11	1	11	11	1	127
Guinea	11	11	1	11	11	11	11	11	11	147
Guinea-Bissau	11	11	11	1	11	11	11	1	11	147
Guyana	11	11	11	11	1	11	1	11	11	157
Haiti	11	11	11	1	11	11	11	11	11	157
Honduras	11	11	11	1	11	11	11	11	11	167
India	11	11	11	1	11	11	11	11	11	167
Indonesia	1	11	11	11	1	11	11	11	11	147
Ivory Coast	11	11	11	1	11	11	11	11	11	157
Kenya	1	11	11	11	11	11	1	11	11	157
Kiribati	11	11	11	11	11	11	11	11	11	177
Kyrgyzstan	11	11	11	11	11	11	11	11	11	157
Lao People's Democratic Republic	11	11	11	1	11	11	11	11	1	157
Lesotho	11	1	11	11	11	11	1	11	11	147
Liberia	11	11	11	11	1	11	11	11	11	157
Madagascar	11	1	11	11	11	11	1	11	11	147
Malawi	11	1	11	1	11	11	11	1	11	137
Mali	11	11	11	1	11	11	11	1	11	137
Mauritania	11	11	11	11	1	11	11	11	1	157
Moldova	11	11	1	11	11	11	11	11	11	167
Mongolia	11	11	1	11	11	11	11	1	11	147
Mozambique	1	11	11	11	11	1	11	11	1	147
Myanmar	11	11	11	11	11	11	11	1	11	167
Nepal	11	11	11	1	11	11	11	1	1	127
Nicaragua	11	11	11	11	11	11	11	11	11	187
Niger	11	11	11	1	11	11	11	11	11	167
Nigeria	1	11	11	11	1	1	11	11	1	117
Pakistan	11	11	11	11	1	11	11	11	11	157
Papua New Guinea	11	11	11	11	11	11	11	11	11	177
Republic of the Con..	11	11	1	11	11	11	11	11	11	157
Rwanda	11	11	1	11	11	1	11	1	11	147
Sao Tome & Principe	11	11	11	1	11	11	1	11	11	147
Senegal	11	11	1	11	11	11	11	11	1	97
Sierra Leone	11	11	1	11	11	1	11	1	11	127
Solomon Islands	11	11	11	11	11	11	11	11	11	187
Somalia	11	11	11	1	11	11	11	11	11	177
South Sudan	11	11	11	11	11	11	11	11	11	187
Sri Lanka	11	11	11	11	11	11	11	11	11	187
Sudan	11	11	11	11	11	11	11	1	11	167
Syria	11	11	11	1	11	11	11	11	11	177
Tajikistan	11	11	1	11	11	11	11	11	11	157
Tanzania	11	11	1	11	11	11	11	1	11	157
Timor-Leste	11	11	11	11	11	11	1	11	11	167
Togo	11	11	11	1	11	11	11	1	11	147
Turkmenistan	11	11	11	1	11	11	11	11	11	167
Uganda	11	11	11	1	11	11	11	11	1	157
Ukraine	11	11	11	11	11	11	11	11	11	177
Uzbekistan	11	11	11	1	11	11	11	11	11	177
Vietnam	11	11	11	11	11	11	11	11	1	167
Yemen	11	11	11	1	11	11	11	11	11	167
Zambia	11	11	11	11	1	11	11	11	11	157
Zimbabwe	11	11	11	1	11	11	1	11	1	127
Total	788	798	728	588	768	798	778	668	718	12,446
Country	Year								Total	
	2012	2013	2014	2015	2016	2017	2018	2019		
Afghanistan	11	11	11	1	11	11	11	11	167	
Albania	11	11	11	11	11	11	1	11	167	
Angola	11	11	11	11	1	11	11	11	177	
Armenia	11	11	11	11	11	11	11	11	167	
Azerbaijan	11	11	11	11	11	11	11	11	177	
Bangladesh	11	11	1	11	11	11	1	11	127	
Benin	1	11	1	11	11	11	1	11	147	
Bhutan	11	11	11	11	11	11	11	11	187	
Bolivia	11	11	11	11	11	11	11	11	167	
Bosnia & Herzegovina	1	11	11	11	11	11	11	11	167	
Burkina Faso	11	11	11	11	11	11	11	11	157	
Burundi	11	11	11	11	11	1	11	11	157	
Cambodia	11	11	1	11	11	11	11	11	157	
Cameroon	11	11	1	11	11	11	1	11	147	
Central African Rep..	11	11	11	11	11	11	11	1	157	
Chad	11	11	11	1	11	11	11	1	147	
China	11	11	11	11	11	11	11	11	187	
Comoros	1	11	11	11	11	11	11	11	177	
Cuba	11	11	11	11	11	11	11	11	187	
Democratic People's Republic of Korea	11	11	11	11	11	1	11	11	177	
Democratic Republic of the Congo	11	11	1	11	11	11	1	11	147	
Djibouti	11	11	11	11	11	11	11	11	187	
Eritrea	11	11	11	11	11	11	11	11	187	
Ethiopia	11	11	11	11	1	11	11	1	147	
Gambia	11	1	11	11	11	11	1	11	147	
Georgia	11	11	11	11	11	11	11	11	187	
Ghana	11	11	1	11	11	11	1	11	127	
Guinea	1	11	11	11	1	11	1	11	147	
Guinea-Bissau	11	11	1	11	11	11	11	1	147	
Guyana	11	11	1	11	11	11	11	11	157	
Haiti	1	11	11	11	11	1	11	11	157	
Honduras	1	11	11	11	11	11	11	11	167	
India	11	11	11	11	1	11	11	11	167	
Indonesia	1	11	11	11	11	1	11	11	147	
Ivory Coast	1	11	11	11	1	11	11	11	157	
Kenya	11	11	1	11	11	11	11	11	157	
Kiribati	11	11	11	11	11	11	11	1	177	
Kyrgyzstan	1	11	1	11	11	11	1	11	157	
Lao People's Democratic Republic	11	11	11	11	11	1	11	11	157	
Lesotho	11	11	1	11	11	11	1	11	147	
Liberia	11	1	11	11	1	11	11	11	157	
Madagascar	1	11	11	11	11	11	1	11	147	
Malawi	11	11	1	11	1	11	11	11	137	
Mali	11	1	11	1	11	11	1	11	137	
Mauritania	11	11	11	1	11	11	11	11	157	
Moldova	1	11	11	11	11	11	11	11	167	
Mongolia	11	11	1	11	11	11	1	11	147	
Mozambique	11	11	11	1	11	11	11	11	147	
Myanmar	11	11	11	11	1	11	11	11	167	
Nepal	11	11	1	11	1	11	11	1	127	
Nicaragua	11	11	11	11	11	11	11	11	187	
Niger	1	11	11	11	11	11	11	11	167	
Nigeria	11	1	11	11	11	1	1	11	117	
Pakistan	11	1	11	11	11	11	1	11	157	
Papua New Guinea	11	11	11	11	11	11	1	11	177	
Republic of the Con..	1	11	11	1	11	11	11	11	157	
Rwanda	11	11	11	1	11	11	11	11	147	
Sao Tome & Principe	11	11	1	11	11	11	11	1	147	
Senegal	11	1	1	1	1	1	1	1	97	
Sierra Leone	11	1	11	11	11	1	11	1	127	
Solomon Islands	11	11	11	11	11	11	11	11	187	
Somalia	11	11	11	11	11	11	11	11	177	
South Sudan	11	11	11	11	11	11	11	11	187	
Sri Lanka (former)	11	11	11	11	11	11	11	11	187	
Sudan	11	11	1	11	11	11	11	11	167	
Syria	11	11	11	11	11	11	11	11	177	
Tajikistan	1	11	11	11	11	1	11	11	157	
Tanzania	11	11	11	11	1	11	11	11	157	
Timor-Leste	11	11	11	11	1	11	11	11	167	
Togo	11	11	1	11	11	1	11	11	147	
Turkmenistan	11	11	11	11	1	11	11	11	167	
Uganda	11	11	11	11	1	11	11	11	157	
Ukraine	1	11	11	11	11	11	11	11	177	
Uzbekistan	11	11	11	11	11	11	11	11	177	
Vietnam	11	11	1	11	11	11	11	11	167	
Yemen	11	1	11	11	11	11	11	11	167	
Zambia	11	11	1	11	11	11	1	11	157	
Zimbabwe	11	11	1	1	11	11	11	1	127	
Total	708	778	648	768	718	758	678	758	12,446	

Next, we specified censored normal distribution models for each outcome of interest – geographic and socioeconomic vaccination equity – given that they are continuous measures with discrete minimum and maximum values. We then determined the optimal number of trajectory groups to include in each model: holding all other parameters constant, we estimated models with two, three, four, and five groups and found that a five-group model produced high Bayesian information criterion (BIC) and entropy values for the geographic equity model, while four-group and two-group specifications proved optimal for the non-imputed and imputed socioeconomic equity models, respectively. For comparison, Appendices C-F (containing our Stata output), also include diagnostics for the other models considered. Using procedures described by Soper, Cohen, and Westland, we estimated that a minimum of 1,599 observations would be required for the five-group geographic equity model to detect a small association (0.1) between integration and equity at 80% statistical power (*p* = 0.05), 150 observations to detect a medium association (0.3), and 38 observations to detect a large association (0.5). To achieve the same level of statistical power, the four-group socioeconomic equity model would require 1,454 observations to detect small associations, 137 observations to detect medium associations, and 34 observations to detect large associations [66–68]. The two-group socioeconomic equity model would require 947, 90, and 23 observations to detect small, medium, and large associations, respectively. Therefore, we concluded that the large number of observations across countries would sufficiently power our analysis to at least detect large associations over the study period.

To determine whether equity trajectories depend on integration in addition to time, we included integration as a time-varying covariate in all models. We then toggled the polynomial order of the imputed five-group geographic equity model and found that a linear specification for all groups produced high BIC and entropy values. A combination of intercept and linear polynomials produced a robust, non-imputed, four-group socioeconomic equity model, while linear polynomials alone proved sufficient in the imputed, two-group socioeconomic equity model. We used parametric bootstrap sampling to estimate group size confidence intervals. Next, we performed several diagnostic checks for each model: calculating average posterior probabilities of group assignment, determining the odds of correct group classification, and conducting a visual inspection of confidence intervals in resultant trajectory plots (Table 2) [69]. Finally, we performed multinomial logistic regression to identify predictors of group membership.

**Table 2:** Group-based trajectory model diagnostics

	**Geographic Equity (non-imputed model)**	**Geographic Equity (imputed model)**	**Socioeconomic Equity (non-imputed model)**	**Socioeconomic Equity (imputed model)**
**Model Diagnostics**				
**Bayesian information criterion**	-3786.16	-44042.75	109.11	1089.08
**Entropy**	0.951	0.896	0.701	0.735
**Average posterior probability of group assignment**	Group 1: 0.995 Group 2: 0.967 Group 3: 0.962 Group 4: 0.974 Group 5: 0.959	Group 1: 0.998 Group 2: 0.941 Group 3: 0.928 Group 4: 0.978 Group 5: 0.932	Group 1: 0.783 Group 2: 0.838 Group 3: 0.919 Group 4: 0.887	Group 1: 0.908 Group 2: 0.935
**Odds of correct group classification**	Group 1: 1419.48 Group 2: 135.58 Group 3: 70.24 Group 4: 91.29 Group 5: 159.73	Group 1: 2992.39 Group 2: 72.95 Group 3: 34.12 Group 4: 117.37 Group 5: 73.70	Group 1: 3.99 Group 2: 14.98 Group 3: 37.65 Group 4: 195.76	Group 1: 3.45 Group 2: 41.16

This table presents several diagnostics for each of our group-based trajectory models. Per Nagin, Bayesian information criterion values and entropy values should be as large as possible, average posterior probabilities should be at least 0.7, and the odds of correct classification should ideally be 5 or greater

As it did not qualify as human subjects research, this investigation was deemed exempt from full review by the Institutional Review Board at the Johns Hopkins Bloomberg School of Public Health (FWA #00000287).

## Results

### Country characteristics

Our sample consisted of 78 countries that have ever received Gavi support. The majority of the countries in the sample reside in sub-Saharan Africa (*n* = 40, 51.3%), per the World Bank’s regional classification scheme, while 12 countries reside in East Asia and the Pacific (15.4%), 11 in Europe and Central Asia (14.1%), 6 in Latin America and the Caribbean (7.7%), 2 in the Middle East and North Africa (2.6%), and 7 in South Asia (9%). Additionally, the World Bank classifies 28 countries (35.9%) in the sample as low-income, 40 (51.3%) as lower-middle-income, and 10 (12.8%) as upper-middle-income. Across both the imputed and non-imputed datasets, mean geographic and socioeconomic equity were highest in Europe & Central Asia and lowest in sub-Saharan Africa. Furthermore, upper-middle-income countries reported the highest mean levels of equity across both datasets, while low-income countries reported the lowest mean levels. Complete demographic details for the countries, along with mean geographic and socioeconomic equity measures disaggregated by region and income level, are summarized in Table 3.

**Table 3:** Summary of country characteristics

	**Total**	**Non-Imputed Dataset**		**Imputed Dataset**	
	**Countries *N* (%)**	**Mean geographic equity (SD)**	**Mean socioeconomic equity (SD)**	**Mean geographicequity (SD)**	**Mean socioeconomic equity (SD)**
**Region**					
**East Asia and the Pacific**	12 (15.4)	67.71 (31.72)	0.193 (0.160)	67.4 (31.88)	0.193 (0.157)
**Europe and Central Asia**	11 (14.1)	92.8 (19.0)	-0.0053 (0.102)	88.1 (25.53)	-0.0053 (0.0998)
**Latin America and the Caribbean**	6 (7.7)	75.56 (29.16)	0.0923 (0.150)	75.78 (29.09)	0.0923 (0.143)
**Middle East and North Africa**	2 (2.6)	47.36 (24.29)	0.273 (0.164)	48.19 (23.8)	0.273 (0.136)
**South Asia**	7 (9)	75.68 (20.49)	0.214 (0.187)	74.64 (21.4)	0.214 (0.182)
**Sub-Saharan Africa**	40 (51.3)	62.36 (26.64)	0.246 (0.189)	62.11 (26.87)	0.246 (0.188)
**Income Level**					
**Low-income**	28 (35.9)	63.74 (26.51)	0.222 (0.167)	63.43 (26.81)	0.222 (0.166)
**Lower-middle-income**	40 (51.3)	65.99 (29.01)	0.223 (0.202)	65.32 (29.46)	0.223 (0.202)
**Upper-middle-income**	10 (12.8)	95.32 (12.46)	0.011 (.134)	94.5 (14.78)	0.011 (0.13)

This table reflects regional and income level classifications as specified by the World Bank in 2022. Geographic equity refers to the proportion of districts within a country that have achieved 80% MCV1 coverage or greater. Socioeconomic equity refers to the slope index of inequality (SII) based on MCV1 coverage by wealth quintile. Larger SII values indicate that higher MCV1 coverage is more prevalent among wealthier quintiles

### Integrated vaccine delivery

Countries demonstrated varying levels of integrated vaccine delivery (hereinafter referred to as “integration,” as defined by the Gavi and the Working Group’s metric for integrated vaccine delivery) over the study period. Among low-income countries, 11 (out of 28 total, 39.3%) demonstrated integration of all four core services at various points between 2003 and 2019, compared to 20 (50%) lower-middle-income countries and 1 (10%) upper-middle-income country. Integrated vaccine delivery also varied geographically: 5 countries in East Asia and the Pacific (41.7% of all countries in the region), 3 (50%) countries in Latin America and the Caribbean, 5 (55.6%) countries in South Asia, and 19 (47.5%) countries in sub-Saharan Africa demonstrated integration at least once during the study period. By contrast, there were 46 countries that never demonstrated integration during the study period. Most of these countries reside in sub-Saharan Africa (*n* = 21), followed by Europe and Central Asia (*n* = 11), East Asia and the Pacific (*n* = 7), Latin America and the Caribbean (*n* = 3), the Middle East and North Africa (*n* = 2), and South Asia (*n* = 2). Table 4 summarizes integration scores earned by each country during the study period.

**Table 4:** Integrated vaccine delivery by country, 2003-2019

**Country**	**Integration Score**		**Total Observations**
	**0**	**1**	
**Afghanistan**	17	0	17
**Albania**	17	0	17
**Angola**	17	0	17
**Armenia**	17	0	17
**Azerbaijan**	17	0	17
**Bangladesh**	17	0	17
**Benin**	17	0	17
**Bhutan**	15	2	17
**Bolivia**	16	1	17
**Bosnia & Herzegovina**	17	0	17
**Burkina Faso**	15	2	17
**Burundi**	16	1	17
**Cambodia**	16	1	17
**Cameroon**	14	3	17
**Central African Republic**	17	0	17
**Chad**	17	0	17
**China**	17	0	17
**Comoros**	16	1	17
**Cuba**	17	0	17
**Democratic People's Republic of Korea**	15	2	17
**Democratic Republic of the Congo**	17	0	17
**Djibouti**	16	1	17
**Eritrea**	16	1	17
**Ethiopia**	17	0	17
**Gambia**	14	3	17
**Georgia**	17	0	17
**Ghana**	11	6	17
**Guinea**	17	0	17
**Guinea-Bissau**	17	0	17
**Guyana**	14	3	17
**Haiti**	17	0	17
**Honduras**	15	2	17
**India**	16	1	17
**Indonesia**	17	0	17
**Ivory Coast**	17	0	17
**Kenya**	17	0	17
**Kiribati**	15	2	17
**Kyrgyzstan**	17	0	17
**Lao People's Democratic Republic**	17	0	17
**Lesotho**	15	2	17
**Liberia**	17	0	17
**Madagascar**	17	0	17
**Malawi**	16	1	17
**Mali**	17	0	17
**Mauritania**	17	0	17
**Moldova**	17	0	17
**Mongolia**	17	0	17
**Mozambique**	15	2	17
**Myanmar**	16	1	17
**Nepal**	15	2	17
**Nicaragua**	17	0	17
**Niger**	17	0	17
**Nigeria**	17	0	17
**Pakistan**	16	1	17
**Papua New Guinea**	17	0	17
**Republic of the Congo (Brazzaville)**	17	0	17
**Rwanda**	15	2	17
**Sao Tome & Principe**	14	3	17
**Senegal**	13	4	17
**Sierra Leone**	16	1	17
**Solomon Islands**	17	0	17
**Somalia**	17	0	17
**South Sudan**	17	0	17
**Sri Lanka**	14	3	17
**Sudan**	16	1	17
**Syria**	17	0	17
**Tajikistan**	17	0	17
**Tanzania**	15	2	17
**Timor-Leste**	17	0	17
**Togo**	15	2	17
**Turkmenistan**	17	0	17
**Uganda**	17	0	17
**Ukraine**	17	0	17
**Uzbekistan**	17	0	17
**Vietnam**	14	3	17
**Yemen**	17	0	17
**Zambia**	17	0	17
**Zimbabwe**	16	1	17
**Total**	**1,263**	**63**	**1326**

^*^ This table displays the number of years in which a country earned a given integration score (0 or 1) over the course of the 17-year study period (2003-2019).

1: coverage of at least one of the four services is at or above 70%, and co-coverage levels are within 10 percentage points of each other.

0: The country did not meet the criteria for a score of “1.”

### Trajectory analysis

**Fig. 1:**
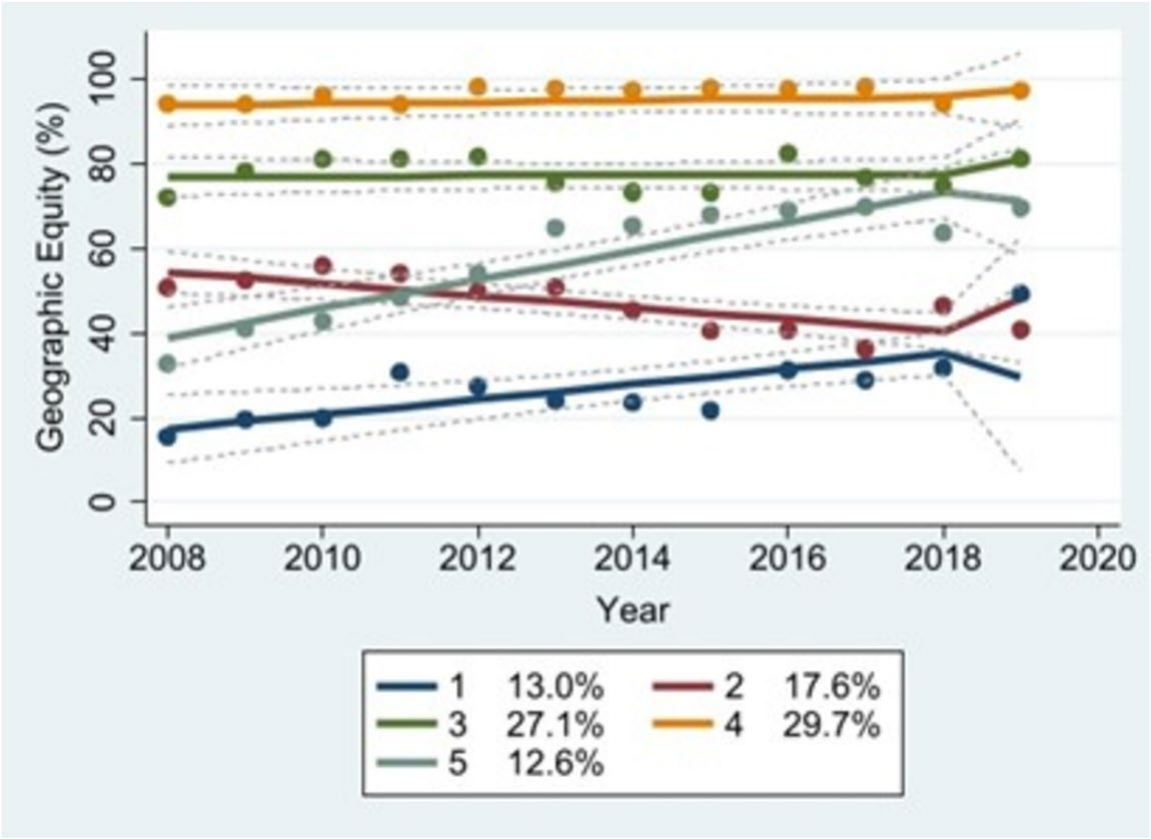
Geographic equity (non-imputed)

**Fig. 2:**
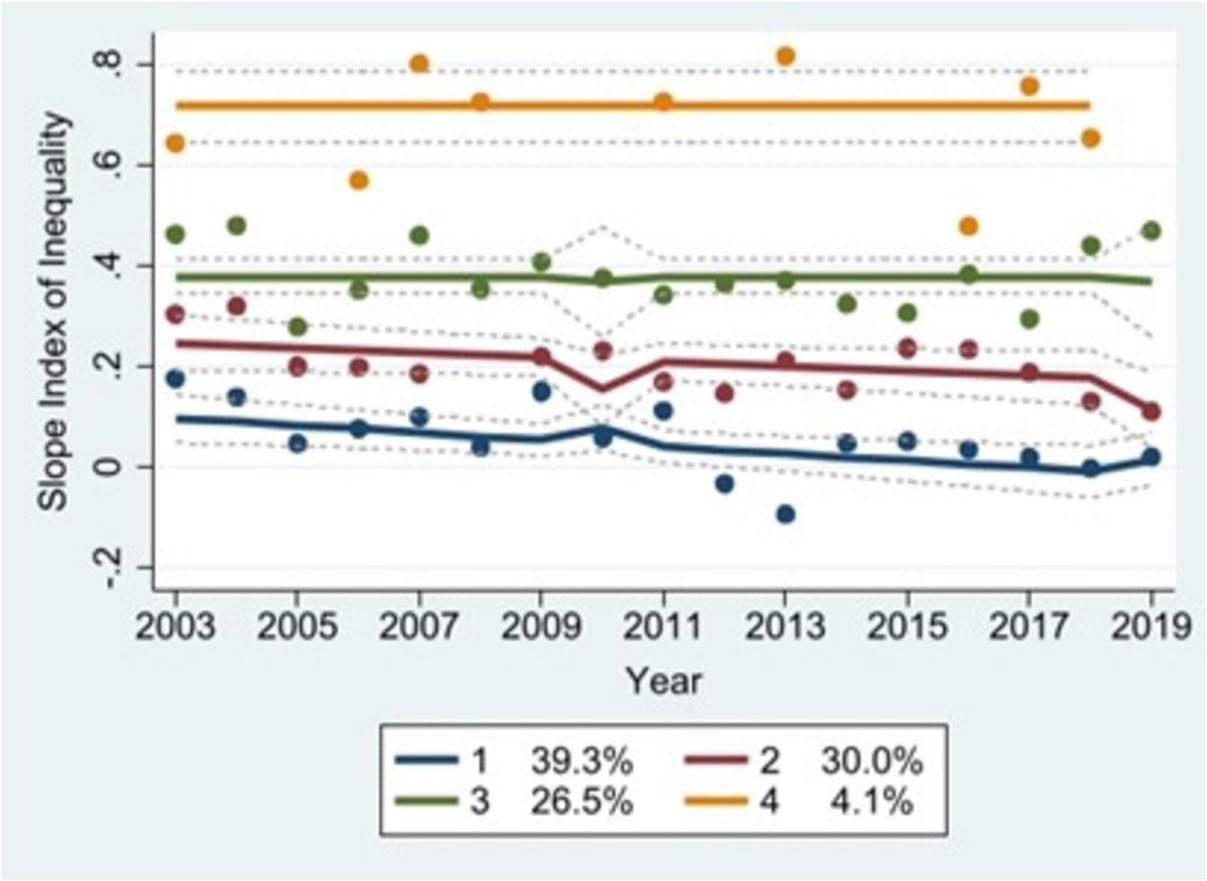
Socioeconomic equity (non-imputed)

**Fig. 3:**
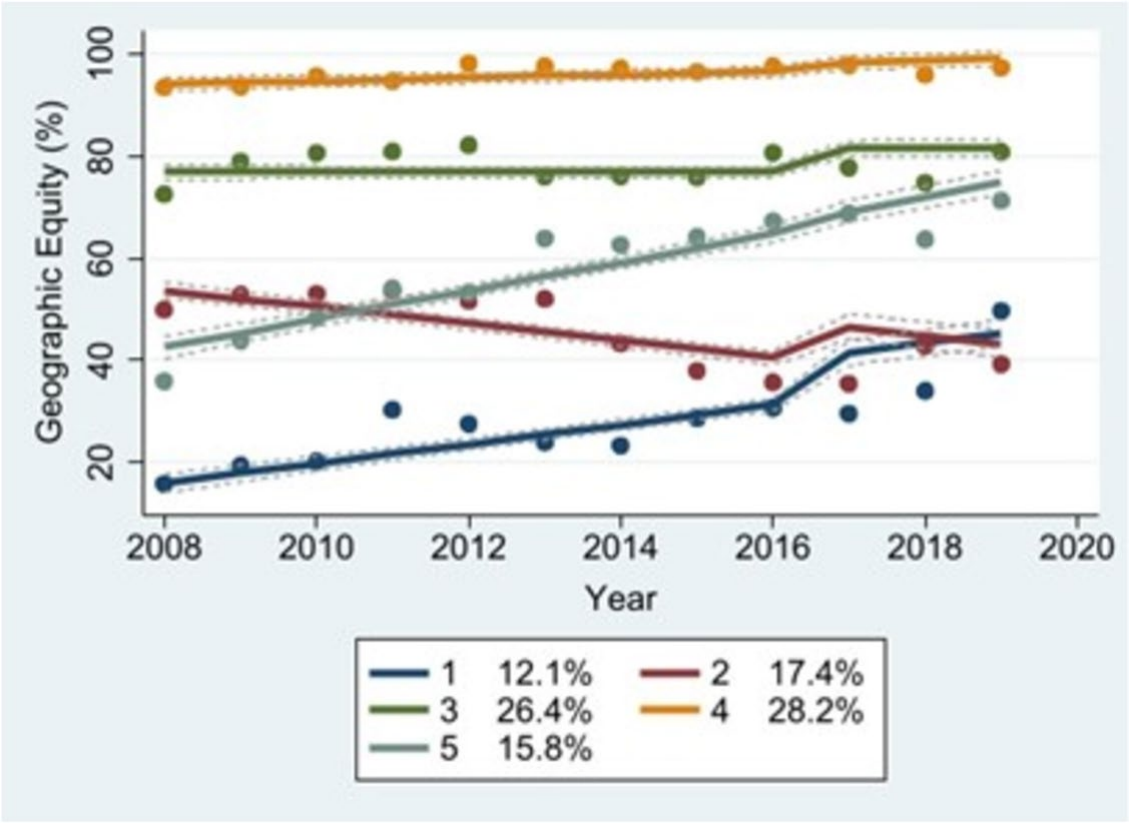
Geographic equity (imputed)

**Fig. 4:**
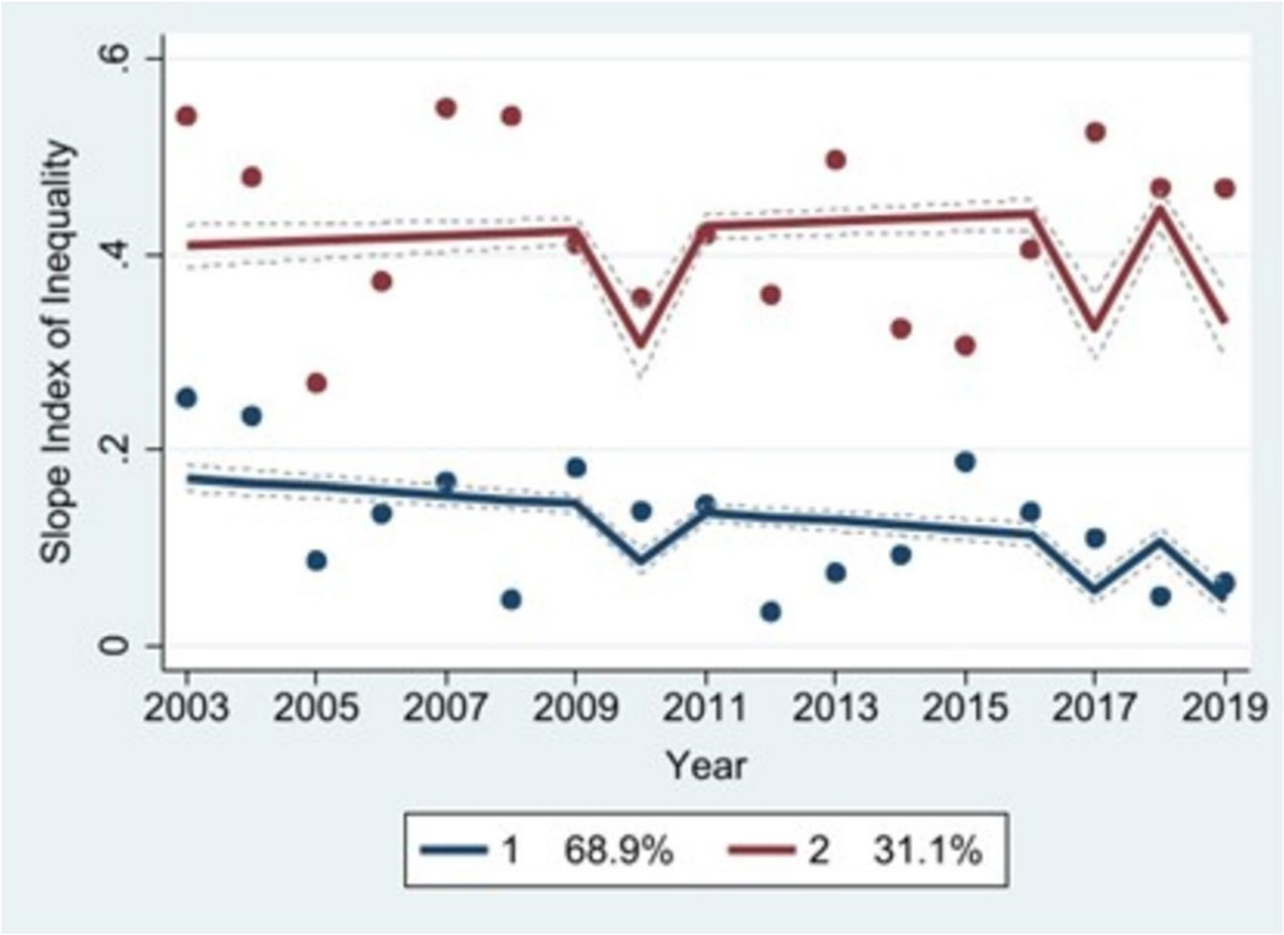
Socioeconomic equity (imputed)

Tables 5 and 6 present the maximum likelihood estimates for each model’s parameters, Tables 7 and 8 display the confidence intervals for each model’s equity estimates, Table 9 lists countries by group assignment, Tables 10 and 11 describe predictors of group membership, and Figures 1, 2, 3 and 4 illustrate the trajectories of geographic and socioeconomic equity over time. The geographic equity model produced five distinct trajectories across both the imputed and non-imputed datasets: a “low-increasing” curve (Group 1, blue) representing countries with relatively few districts with high MCV1 coverage across the study period; a “middle-decreasing” curve (Group 2, red) representing countries with middling proportions of districts with high MCV1 coverage; a “middle-stable” curve (Group 3, dark green) representing countries with fairly high and consistent proportions of districts with high MCV1 coverage; a “high-stable” curve (Group 4, orange) representing countries with the highest consistent proportions of districts with high MCV1 coverage; and a “middle-increasing” curve (Group 5, light green) representing countries with middling proportions of districts with high MCV1 coverage that gradually increased over the study period. In the non-imputed dataset, Groups 1-5 included roughly 13%, 18%, 27%, 30%, and 13% of the countries in our sample, respectively. The model based on the imputed dataset assigned 12%, 17%, 26%, 28%, and 16% of the countries to Groups 1-5, respectively.

**Table 5:** Maximum likelihood estimates: geographic equity

		**Non-Imputed Dataset**		**Imputed Dataset**	
**Group**	**Parameter**	**Estimate**	**Probability > |t|**	**Estimate**	**Probability > |t|**
**1 ("Low-increasing")**	Intercept	-3726.42	0.00	-3828.30	0.00
	Linear	1.86	0.00	1.91	0.00
	Integration	-7.71	0.39	8.32	0.00
**2 ("Middle-increasing")**	Intercept	2864.77	0.0001	3404.92	0.00
	Linear	1.40	0.0001	-1.67	0.00
	Integration	9.01	0.21	7.90	0.00
**3 ("Middle-stable")**	Intercept	8.82	0.99	40.05	0.84
	Linear	0.034	0.91	0.018	0.85
	Integration	3.87	0.30	4.60	0.00
**4 ("High-stable")**	Intercept	-368.32	0.50	-498.04	0.006
	Linear	0.23	0.40	0.29	0.001
	Integration	1.75	0.61	1.58	0.018
**5 ("High-stable")**	Intercept	-6808.17	0.00	-5519.41	0.00
	Linear	3.41	0.00	2.77	0.00
	Integration	-5.38	0.41	1.72	0.17
**Group Membership**					
**1 ("Low-increasing")**		13.04%	0.009	12.07%	0.00
**2 ("Middle-increasing")**		17.62%	0.0001	17.45%	0.00
**3 ("Middle-stable")**		27.05%	0.00	26.44%	0.00
**4 ("High-stable")**		29.68%	0.00	28.23%	0.00
**5 ("High-stable")**		12.61%	0.002	15.80%	0.00

**Table 6:** Maximum likelihood estimates: socioeconomic equity

**Non-Imputed Dataset**					**Imputed Dataset**			
**Group**	**Parameter**	**Estimate**	**Probability > |t|**	**Group**		**Parameter**	**Estimate**	**Probability > |t|**
**1 ("Low-inequity")**	Intercept	14.12	0.008	**1 ("Low-stable")**		Intercept	8.93	0.00
	Linear	-0.01	0.008			Linear	-0.0044	0.00
	Integration	0.03	0.26			Integration	-0.0054	0.00
**2 ("Medium-decreasing")**	Intercept	9.46	0.09	**2 ("High-stable")**		Intercept	-4.48	0.04
	Linear	-0.005	0.10			Linear	0.0024	0.026
	Integration	0.06	0.10			Integration	-0.12	0.000
**3 ("Medium-stable")**	Intercept	0.38	0.00					
	Integration	-0.012	0.84					
**4 ("High-inequity")**	Intercept	0.72	0					
	Integration	-0.53	0.0001					
**Group Membership**								
**1 ("Low-inequity")**		39.33%	0.00	**1 ("Low-stable")**			68.85%	0.00
**2 ("Medium-decreasing")**		30.01%	0.0001	**2 ("High-stable")**			31.15%	0.00
**3 ("Medium-stable")**		26.53%	0.00					
**4 ("High-inequity")**		4.12%	0.14

**Table 7:** Parametric bootstrap sampling confidence interval estimates: geographic equity

		**Non-Imputed Dataset**			**Imputed Dataset**		
**Group**	**Parameter**	**Observed Coefficient**	**Probability > |*z*|**	**95% Confidence Interval (Bias Corrected)**	**Observed Coefficient**	**Probability > |*z*|**	**95% Confidence Interval (Bias Corrected)**
**1 ("Low-increasing")**	Intercept	-3726.40	0.06	(-9566.50, -874.15)	-3828.3	0.00	(-4910.96, -2069.20)
	Linear	1.86	0.053	(0.45, 4.77)	1.91	0.00	(1.04, 2.45)
	Integration	-6.34	0.98	(-12.20, 17.89)	8.32	0.00	(4.82, 11.31)
**2 ("Middle-decreasing")**	Intercept	2864.78	0.016	(830.46, 5644.19)	3404.92	0.00	(2790.18, 4177.75)
	Linear	-1.40	0.018	(-2.78, -0.39)	-1.67	0.00	(-2.05, -1.36)
	Integration	8.88	0.74	(-0.94, 29.86)	7.89	0.00	(5.46, 10.23)
**3 ("Middle-stable")**	Intercept	8.82	1.00	(-1935.68, 11040.6)	40.05	0.93	(-777.67, 968.02)
	Linear	0.033	0.97	(-5.44, 0.997)	0.02	0.94	(-0.44, 0.42)
	Integration	3.87	0.99	(-2.42, 7.85)	4.60	0.00	(2.50, 6.30)
**4 ("High-stable")**	Intercept	-368.32	0.45	(-1181.89, 874.43)	-498.04	0.001	(-787.43, -212.46)
	Linear	0.23	0.34	(-0.42, 0.63)	0.29	0.00	(0.15, 0.44)
	Integration	1.75	0.18	(-1.20, 4.04)	1.58	0.00	(0.83, 2.60)
**5 ("Middle-increasing")**	Intercept	-6808.17	0.014	(-11790.93, -1102.48)	-5519.41	0.00	(-9723.36, -3875.15)
	Linear	3.41	0.013	(0.58, 5.88)	2.77	0.00	(1.95, 4.85)
	Integration	-5.41	0.996	(-644.69, 287.75)	1.72	0.55	(-2.45, 9.01)

**Table 8:** Parametric Bootstrap sampling confidence interval estimates: socioeconomic equity

	**Non-Imputed Dataset**				**Imputed Dataset**			
**Group**	**Parameter**	**Observed Coefficient**	**Probability > |*z*|**	**95% Confidence Interval (Bias Corrected)**	**Parameter**	**Observed Coefficient**	**Probability > |*z*|**	**95% Confidence Interval (Bias Corrected)**
**1**	Intercept	14.12	0.022	(-1.22, 23.94)	Intercept	8.93	0.00	(5.42, 11.71)
	Linear	-0.007	0.022	(-0.012, 0.00054)	Linear	-0.004	0.00	(-0.006, -0.003)
	Integration	0.031	0.43	(-0.05, .10)	Integration	-0.05	0.00	(-0.08, -0.04)
**2**	Intercept	9.46	0.29	(-12.91, 22.51)	Intercept	-4.48	0.005	(-7.48, -1.21)
	Linear	-0.005	0.30	(-0.011, 0.007)	Linear	0.0024	0.002	(0.0008, 0.004)
	Integration	-0.061	0.33	(-0.26, 0.018)	Integration	-0.12	0.00	(-0.17, -0.08)
**3**	Intercept	0.38	0.00	(0.33, 0.47)				
	Integration	-0.011	0.90	(-0.35, 0.09)				
**4**	Intercept	0.72	0.00	(0.41, 0.74)				
	Integration	-0.53	0.012	(-0.87, -0.25)

**Table 9:** Countries by Group Membership

		**Non-Imputed Dataset**	**Imputed Dataset**	
**Geographic Equity**				
**Group 1**		Central African Republic, Eritrea, Haiti, Lesotho, Mauritania, Papua New Guinea, Solomon Islands, Somalia, Timor-Leste, Ukraine	Central African Republic, Eritrea, Haiti, Lesotho, Mauritania, Papua New Guinea, Solomon Islands, Somalia, Timor-Leste, Ukraine	
**Group 2**		Afghanistan, Angola, Bolivia, Cameroon, Chad, Comoros, Djibouti, Guinea-Bissau, Kenya, Liberia, Republic of the Congo (Brazzaville), South Sudan, Syria, Yemen	Afghanistan, Angola, Bolivia, Bosnia & Herzegovina, Cameroon, Chad, Comoros, Djibouti, Guinea-Bissau, Kenya, Liberia, Republic of the Congo (Brazzaville), South Sudan, Ukraine, Yemen	
**Group 3**		Benin, Bosnia & Herzegovina, Burundi, Cambodia, Democratic Republic of the Congo, Ghana, Guinea, Honduras, India, Indonesia, Kiribati, Madagascar, Malawi, Mali, Mongolia, Mozambique, Myanmar, Niger, Sierra Leone, Togo Zambia	Benin, Bhutan, Bosnia & Herzegovina, Burundi, Cambodia, Democratic Republic of the Congo, Ghana, Guinea, Honduras, India, Indonesia, Kiribati, Kyrgyzstan, Madagascar, Malawi, Mali, Mongolia, Mozambique, Myanmar, Niger, Rwanda, Sierra Leone, Syria, Tanzania, Togo, Zambia	
**Group 4**		Albania, Armenia, Azerbaijan, Bangladesh, Bhutan, Burkina Faso, China, Cuba Democratic People's Republic of Korea, Gambia, Georgia, Guyana, Kyrgyzstan, Moldova, Nicaragua, Rwanda, Sao Tome & Principe, Sri Lanka, Tajikistan, Tanzania, Turkmenistan, Uzbekistan, Vietnam	Albania, Armenia, Azerbaijan, Bangladesh, Bhutan, Burkina Faso, China, Cuba, Democratic People's Republic of Korea, Gambia, Georgia, Guyana, Kyrgyzstan, Moldova, Nicaragua, Rwanda, Sao Tome and Principe, Sri Lanka, Tajikistan, Tanzania, Turkmenistan, Uzbekistan, Vietnam	
**Group 5**		Ethiopia, Ivory Coast, Lao People's Democratic Republic, Nepal, Nigeria, Pakistan, Senegal, Sudan, Uganda, Zimbabwe	Ethiopia, India, Ivory Coast, Kiribati, Lao People's Democratic Republic, Nepal, Nigeria, Pakistan, Senegal, Sudan, Syria, Uganda, Zimbabwe	
**Socioeconomic Equity**				
**Group 1**	Afghanistan, Albania, Armenia, Bhutan, Bolivia, Bosnia & Herzegovina, Burundi, China, Cuba, Democratic People's Republic of Korea, Djibouti, Eritrea, Gambia, Georgia, Ghana, Guyana, Honduras, Kiribati, Kyrgyzstan, Lesotho, Malawi, Moldova, Mongolia, Nepal, Nicaragua, Rwanda, Sao Tome & Principe, Sierra Leone, Solomon Islands, Sri Lanka, Syria, Tajikistan, Turkmenistan, Uganda, Ukraine, Uzbekistan, Vietnam			Afghanistan, Albania, Armenia, Azerbaijan, Bangladesh, Bhutan, Bolivia, Bosnia & Herzegovina, Burkina Faso, Burundi, Cambodia, Chad, China, Comoros, Cuba, Democratic People's Republic of Korea, Djibouti, Eritrea, Gambia, Georgia, Ghana, Guinea-Bissau, Guyana, Haiti, Honduras, Indonesia, Kenya, Kiribati, Kyrgyzstan, Lesotho, Malawi, Mali, Mauritania, Moldova, Mongolia, Myanmar, Nepal, Nicaragua, Niger, Rwanda, Sao Tome & Principe, Senegal, Sierra Leone, Solomon Islands, Somalia, Sri Lanka, Syria, Tajikistan, Tanzania, Timor-Leste, Togo, Turkmenistan, Uganda, Ukraine, Uzbekistan, Vietnam, Zambia
**Group 2**	Azerbaijan, Bangladesh, Burkina Faso, Cambodia, Chad, Comoros, Guinea-Bissau, Haiti, Indonesia, Kenya, Mali, Mauritania, Myanmar, Niger, Senegal, Somalia, Tanzania, Togo, Zambia, Zimbabwe			Angola, Benin, Cambodia, Cameroon, Central African Republic, Democratic Republic of the Congo, Ethiopia, Guinea, India, Ivory Coast, Lao People's Democratic Republic, Liberia, Madagascar, Mozambique, Nigeria, Pakistan, Papua New Guinea, Republic of the Congo (Brazzaville), South Sudan, Sudan, Yemen
**Group 3**	Benin, Cameroon, Central African Republic Democratic Republic of the Congo, Ethiopia, Guinea, Ivory Coast, Lao People's Democratic Republic Liberia, Madagascar, Mozambique, Pakistan, Papua New Guinea, Republic of the Congo (Brazzaville), South Sudan, Sudan, Timor-Leste, Yemen			
**Group 4**	Angola, India, Nigeria

**Table 10:** Predictors of group membership: geographic equity

		**Non-Imputed Dataset**			**Imputed Dataset**		
**Group**	**Predictor of Group Membership**	**Relative Risk**	**P>|*z*|**	**95% Confidence Interval**	**Relative Risk**	**P>|*z*|**	**95% Confidence Interval**
**1 (base outcome)**							
**2**	**Income**	1.23E-06	0.036	(3.65E-12, 0.4159266)	3.74E-06	0	(1.58E-07, 0.000089)
	**Region**	4.44E-06	0.021	(1.24E-10, 0.1587821)	0.0000608	0	(6.75E-06, 0.00055)
	**Female education**	1.525187	0.022	(1.06, 2.19)	1.407719	0	(1.29, 1.54)
	**Political stability**	3.07E+07	0.019	(18.00, 5.25E+13)	578528.8	0	(36110.48, 9268656)
	**Government effectiveness**	1308098	0.091	(0.11, 1.61E+13)	608730	0	(14035.65, 2.64E+07)
	**Corruption**	6.60E-07	0.015	(7.29E-12, 0.06)	5.59E-06	0	(4.32E-07, 0.000072)
	**Gender inequality**	5.50E+10	0.113	(0.003, 1.04E+24)	1.15E+08	0	(202035.3, 6.53E+10)
	**Out-of-pocket health expenditures**	2.00891	0.014	(1.15, 3.51)	1.756889	0	(1.57, 1.97)
	**Government expenditures on health**	1.021184	0.043	(1.00, 1.04)	1.018376	0	(1.01, 1.02)
	**External resources for health per capita**	1.140063	0.055	(0.997, 1.30)	1.089446	0	(1.05, 1.13)
	**Land area**	1.000014	0.07	(1.00, 1.00)	1.000011	0	(1.00, 1.00)
	**Linguistic fractionalization**	1.68E+19	0.014	(9140.60, 3.08E+34)	2.45E+14	0	(1.59E+11, 3.77E+17)
	**Distance**	0.3060222	0.007	(0.13, 0.72)	0.3788097	0	(0.32, 0.46)
	**Integration**	1.971843	0.872	(0.0005, 7573.06)	2.739571	0.036	(1.07, 7.04)
**Group**	**Predictor of Group Membership**	**Relative Risk**	**P>|*z*|**	**95% Confidence Interval**	**Relative Risk**	**P>|*z*|**	**95% Confidence Interval**
**3**	**Income**	1.32E-08	0.006	(2.89E-14, 0.0060)	1.30E-07	0	(5.32E-09, 3.18E-06)
	**Region**	1.46E-07	0.003	(3.83E-12, 0.006)	2.73E-06	0	(2.98E-07, 0.000025)
	**Female education**	1.41196	0.062	(0.98, 2.03)	1.322125	0	(1.21, 1.45)
	**Political stability**	5.05E+07	0.016	(26.09,	479464.5	0	(29547.59, 7780204)
	**Government effectiveness**	2.59E+09	0.011	(137.61, 4.86E+16)	2.05E+08	0	(4576649, 9.19E+09)
	**Corruption**	0.0000239	0.066	(2.78E-10, 2.06)	0.0001559	0	(0.000012, 0.002)
	**Gender inequality**	1.58E+12	0.074	(0.069, 3.62E+25)	1.47E+09	0	(2501470, 8.66E+11)
	**Out-of-pocket health expenditures**	2.144334	0.008	(1.22, 3.78)	1.840724	0	(1.64, 2.07)
	**Government expenditures on health**	0.9566394	0.006	(0.93, 0.99)	0.9690138	0	(0.96, 0.98)
	**External resources for health per capita**	1.223744	0.003	(1.07, 1.40)	1.146691	0	(1.11, 1.18)
	**Land area**	1.000021	0.008	(1.00, 1.00)	1.000016	0	(1.00, 1.00)
	**Linguistic fractionalization**	7.57E+13	0.067	(0.10, 5.57E+28)	8.38E+10	0	(6.00E+07, 1.17E+14)
	**Distance**	0.251116	0.002	(0.11, 0.60)	0.323638	0	(0.27, 0.39)
	**Integration**	6.282047	0.652	(0.0021, 18556.93)	6.276409	0	(2.49, 15.84)
**Group**	**Predictor of Group Membership**	**Relative Risk**	**P>|*z*|**	**95% Confidence Interval**	**Relative Risk**	**P>|*z*|**	**95% Confidence Interval**
**4**	**Income**	1.73E-07	0.018	(4.21E-13, 0.071)	6.08E-07	0	(2.52E-08, 0.0000147)
	**Region**	2.40E-07	0.005	(6.37E-12, 0.0090)	7.78E-06	0	(8.53E-07, 0.0000709)
	**Female education**	1.490188	0.033	(1.03, 2.15)	1.328794	0	(1.21, 1.45)
	**Political stability**	1073.834	0.335	(0.00073, 1.58E+09)	564.8665	0	(34.28, 9308.02)
	**Government effectiveness**	5.32E+12	0.001	(153471.1, 1.85E+20)	3.67E+10	0	(7.56E+08, 1.78E+12)
	**Corruption**	0.013079	0.445	(1.94E-07, 883.11)	0.0259146	0.004	(0.0021, 0.32)
	**Gender inequality**	4.04E+13	0.05	(1.01, 1.62E+27)	5.66E+09	0	(7951203, 4.03E+12)
	**Out-of-pocket health expenditures**	2.444354	0.003	(1.36, 4.38)	2.030428	0	(1.80, 2.29)
	**Government expenditures on health**	1.029165	0.003	(1.01, 1.05)	1.023285	0	(1.02, 1.03)
	**External resources for health per capita**	1.329672	0	(1.15, 1.53)	1.205292	0	(1.17, 1.25)
	**Land area**	0.999959	0.001	(1.00, 1,00)	0.9999704	0	(1,00, 1.00)
	**Linguistic fractionalization**	5.90E+22	0.004	(1.95E+07, 1.78E+38)	6.95E+15	0	(4.34E+12, 1.11E+19)
	**Distance**	0.2893782	0.004	(0.12, 0.68)	0.3631924	0	(0.30, 0.44)
	**Integration**	13.21398	0.521	(0.005, 34832.43)	5.877308	0	(2.32, 14.91)
**Group**	**Predictor of Group Membership**	**Relative Risk**	**P>|*z*|**	**95% Confidence Interval**	**Relative Risk**	**P>|*z*|**	**95% Confidence Interval**
**5**	**Income**	0.0001449	0.169	(4.84E-10, 43.33)	0.0002009	0	(8.08E-06, 0.005)
	**Region**	2.37E-06	0.014	(7.49E-11, 0.075)	0.000014	0	(1.56E-06, 0.0001)
	**Female education**	1.525957	0.023	(1.06, 2.196961)	1.362198	0	(1.25, 1.49)
	**Political stability**	2064366	0.043	(1.61, 2.65E+12)	59958.64	0	(3924.66, 916013)
	**Government effectiveness**	6816403	0.064	(0.39, 1.18E+14)	1431966	0	(31764.32, 6.46E+07)
	**Corruption**	0.0347788	0.548	(5.98E-07, 2021.75)	0.3779067	0.446	(0.031, 4.63)
	**Gender inequality**	9.55E+09	0.141	(0.00050, 1.81E+23)	2.74E+08	0	(456180.3, 1.65E+11)
	**Out-of-pocket health expenditures**	2.179092	0.007	(1.24, 3.83)	1.873801	0	(1.67, 2.10)
	**Government expenditures on health**	0.9757595	0.168	(0.94, 1.010422)	0.9727223	0	(0.96, 0.98)
	**External resources for health per capita**	1.04877	0.534	(0.90, 1.22)	1.040898	0.035	(1.00, 1.08)
	**Land area**	1.000016	0.043	(1.00, 1.00)	1.000013	0	(1.00, 1.00)
	**Linguistic fractionalization**	1.13E+23	0.005	(1.33E+07, 9.61E+38)	9.93E+16	0	(5.17E+13, 1.91E+20)
	**Distance**	0.361731	0.018	(0.16, 0.84)	0.4262153	0	(0.36, 0.51)
	**Integration**	1.393845	0.935	(0.00045, 4306.50)	3.268299	0.017	(1.23, 8.66)

**Table 11:** Predictors of group membership: socioeconomic equity

			**Non-Imputed Dataset**			**Imputed Dataset**				
**Group**		**Predictor of Group Membership**	**Relative Risk**	**P>|*z*|**	**95% Confidence Interval**	**Relative Risk**	**P>|*z*|**	**95% Confidence Interval**		
**1 (base outcome)**										
**2**		**Income**	2.241445	0.361	(0.40, 12.66)	5.307659	0	(3.82, 7.38)		
		**Region**	0.8764091	0.714	(0.43, 1.77)	1.088548	0.093	(0.99, 1.20)		
		**Female education**	0.9141557	0	(0.87, 0.96)	0.990228	0.005	(0.98, 1.00)		
		**Political stability**	2.524515	0.091	(0.86, 7.38)	1.27432	0.004	(1.08, 1.50)		
		**Government effectiveness**	1.342423	0.809	(0.12, 14.56)	1.756637	0.015	(1.12, 2.76)		
		**Corruption**	0.1219006	0.09	(0.011, 1.39)	0.207852	0	(0.13, 0.32)		
		**Gender inequality**	1.73E-06	0	(1.77E-08, 0.00017)	3.338941	0	(1.92, 5.82)		
		**Out-of-pocket health expenditures**	1.012023	0.528	(0.98, 1.05)	0.960434	0	(0.95, 0.97)		
		**Government expenditures on health**	0.9751033	0	(0.96, 0.99)	0.961332	0	(0.96, 0.97)		
		**External resources for health per capita**	0.9015603	0	(0.85, 0.95)	0.974827	0	(0.97, 0.98)		
		**Land area**	1.000006	0	(1.00, 1.00)	1.000001	0	(1.00, 1.00)		
		**Linguistic fractionalization**	1.246866	0.855	(0.12, 13.29)	2.272543	0	(1.45, 3.56)		
		**Distance**	1.018492	0.386	(0.98, 1.06)	1.024245	0	(1.02, 1.03)		
		**Integration**	0.837396	0.853	(0.13, 5.48)	0.489647	0	(0.38, 0.63)		
**Group**		**Predictor of Group Membership**	**Relative Risk**	**P>|*z*|**	**95% Confidence Interval**	**Relative Risk**	**Standard Error**	***z***	**P>|*z*|**	**95% Confidence Interval**
**3**		**Income**	4.655903	0.073	(0.87, 24.95)					
		**Region**	0.9844899	0.965	(0.49, 1.99)					
		**Female education**	0.9395753	0.001	(0.90, 0.98)					
		**Political stability**	1.540575	0.373	(0.60, 3.98)					
		**Government effectiveness**	1.124743	0.924	(0.10, 12.62)					
		**Corruption**	0.0858633	0.038	(0.0085, 0.87)					
		**Gender inequality**	0.000375	0	(5.10E-06, 0.028)					
		**Out-of-pocket health expenditures**	0.9496381	0.011	(0.91, 0.99)					
		**Government expenditures on health**	0.9457635	0	(0.93, 0.97)					
		**External resources for health per capita**	0.8876052	0	(0.84, 0.94)					
		**Land area**	1.000005	0	(1.00, 1.00)					
		**Linguistic fractionalization**	17.64553	0.012	(1.90, 163.90)					
		**Distance**	1.048426	0.016	(1.01, 1.09)					
		**Integration**	0.4460937	0.388	(0.07, 2.79)					
**Group**	**Predictor of Group Membership**		**Relative Risk**	**P>|*z*|**	**95% Confidence Interval**	**Relative Risk**	**Standard Error**	***z***	**P>|*z*|**	**95% Confidence Interval**
**4**	**Income**		5.44E+19	0.996	-					
	**Region**		3451.95	0.997	-					
	**Female education**		0.5746389	0.998	(4.10E-185, 8.10E+183)					
	**Political stability**		0.4704951	1	-					
	**Government effectiveness**		1.117109	1	-					
	**Corruption**		0.0000242	0.999	-					
	**Gender inequality**		0.1436708	1	-					
	**Out-of-pocket health expenditures**		0.9852097	1	(3.30E-228, 3.00E+227)					
	**Government expenditures on health**		1.00599	0.999	(6.19E-06, 163532.6)					
	**External resources for health per capita**		0.7057309	0.999	-					
	**Land area**		1.000039	0.993	(0.99, 1.01)					
	**Linguistic fractionalization**		6.05E-18	0.995	-					
	**Distance**		0.403834	0.993	(1.06E-90, 1.54E+89)					
	**Integration**		1.52547	1	-

The non-imputed socioeconomic equity model produced four distinct trajectories: a “low-inequity” curve (Group 1, blue) representing countries with high MCV1 coverage among poorer wealth quintiles; a “medium-decreasing” curve (Group 2, red) representing countries with slightly lower MCV1 coverage levels among poorer quintiles; a “medium-stable” curve (Group 3, green) representing countries with consistently middling levels of MCV1 coverage among poorer quintiles; and a “high-inequity” curve (Group 4, orange) representing countries with very low MCV1 coverage among poorer quintiles. Groups 1-4 included roughly 39%, 30%, 27%, and 4% of the countries in our sample, respectively. By contrast, the imputed model produced two trajectories: a “low-stable” curve (Group 1, blue) consisting of 69% of countries in the sample, and a “high-stable” curve (Group 2, red) that included 31% of countries. The Group 1 curve represents countries with high levels of MCV1 coverage among poorer quintiles, while the Group 2 curve represents those with MCV1 coverage skewed toward wealthier quintiles.

We did not detect a statistically significant association between integrated vaccine delivery and geographic equity using the non-imputed dataset, or in Groups 1-3 in the non-imputed socioeconomic equity model. However, there was a significant association between the two in countries belonging to Group 4 (high-inequity) in the non-imputed socioeconomic equity model, both groups in the imputed socioeconomic equity model, and Groups 1-4 in the imputed geographic equity model. In the non-imputed dataset, integration was associated with a 0.53-unit reduction in SII in Group 4 (high-inequity). In Groups 1-4 in the imputed dataset, it was associated with 8.3-, 7.9-, 4.6-, and 1.6-percentage point increases per one-unit change in integration score, respectively, in the proportion of districts reporting greater than 80% MCV1 coverage. In this same dataset, integration was associated with a 0.054-unit reduction in SII in Group 1 (low-stable) countries and a 0.12-unit reduction in SII in Group 2 (high-stable) countries.

### Predictors of group membership

Tables 10 and 11 summarize results from our multinomial logistic regression analyses, whereby we identified predictors of group membership for each trajectory model. In the non-imputed geographic equity dataset, we identified several predictors of membership in each group: in Group 2 (middle decreasing), income level, region, female education, political stability, corruption, out-of-pocket health expenditures, government expenditures on health, linguistic fractionalization, and distance (i.e., the proportion of the population more than 60 minutes by foot away from the nearest health facility); in Group 3 (middle-stable), income, region, political stability, government effectiveness, out-of-pocket health expenditures, government expenditures on health, external resources for health per capita, land area, and distance; in Group 4 (high-stable), income level, region, female education, government effectiveness, gender inequality, out-of-pocket health expenditures, government expenditures on health, external resources for health per capita, land area, linguistic fractionalization, and distance; and in Group 5 (middle-increasing), region, female education, political stability, gender inequality, land area, linguistic fractionalization, and distance. Notably, integration was not a significant predictor of membership in any of the five groups. Using the imputed dataset, however, we found that all of the variables included in the regression model were significant predictors of membership in Groups 1-4. Except for corruption, these variables were also significant predictors of Group 5 (middle-increasing) membership.

In the non-imputed socioeconomic equity dataset, we identified several significant predictors of Group 2 (medium-decreasing) membership, including female education, gender inequality, government expenditures on health, external resources for health per capita, and land area. Female education, gender inequality, out-of-pocket health expenditures, government health expenditures, external resources for health per capita, land area, linguistic fractionalization, and distance were also significant predictors of membership in Group 3 (medium-stable). Integration was not a significant predictor of membership in any group, nor did we identify any significant predictors of Group 4 (high-inequity) membership using the non-imputed dataset. Using the imputed dataset, however, we found that with the exception of geographic region, every variable in the model – including integration – was a significant predictor of Group 2 (high-stable) membership.

## Discussion

In this investigation, we applied GBTM to examine the relationship between integrated vaccine delivery and vaccination equity in 78 LMICs that had ever received Gavi support. Using our non-imputed longitudinal dataset, we constructed models of geographic and socioeconomic equity consisting of five and four distinct equity trajectories (i.e., groups), respectively. Though integration was not a statistically significant predictor of group membership in either model, we identified several other important predictors (see Tables 10 and 11). Using our imputed dataset, we developed a second five-group geographic equity model, as well as a two-group socioeconomic equity model. With the exception of corruption, all of the variables included in the multinomial logistic regression – including integration – were significant predictors of group membership in the geographic equity model. Integration, along with all variables except geographic region, was also a significant predictor of membership in Group 2 (high-stable) of the imputed socioeconomic model.

The statistically significant association between integration and Groups 1-4 in the imputed geographic equity model carries important implications for public health policy and practice. This finding suggests that integrated vaccine delivery is most strongly associated with equity improvements in settings with chronically high levels of inequity (i.e., Group 1, low-increasing), followed by those reporting decreasing levels of equity over time (i.e., Group 2), and finally, in settings in which geographic equity has largely plateaued (i.e., Groups 3 and 4). However, it may have a weaker association with equitable vaccination coverage in settings like Group 5 (middle-increasing) countries, whose developmental trajectory commences at a middling level of equity and increases more rapidly than its counterparts in Groups 1-4. In the imputed model, integration was also a predictor of membership in all but the lowest-performing group (Group 1, low-increasing), further suggesting that integration is associated with high geographic equity achievement. Due to our small sample of countries, we were unable to ascertain whether integration might have had small- or medium-sized associations with geographic equity in groups in which we detected no statistically significant associations between the two. Nevertheless, given that measles risk is often spatially clustered, this finding may still be relevant for countries pursuing measles elimination as part of a national or multi-district mitigation or elimination strategy [70].

In the non-imputed model, integration showed a significant association with socioeconomic equity in Group 4 (high-inequity) countries: a 0.53-unit reduction in SII. In the imputed model, integration with associated with two additional statistically significant reductions in SII: a 0.054-unit drop in Group 1 (low-stable) and a 0.12-unit drop in Group 2 (high-stable). This suggests that integrated vaccine delivery may be most strongly associated with equity improvements in settings with high vaccination inequity at baseline. In this vein, we found that the relative risk of a country being assigned to Group 2 (high-inequity) would decrease by a factor of 0.49 if the country in question demonstrated integration (see Table 11). In settings with lower levels of socioeconomic inequity (i.e., Group 1 countries, low-inequity), integration may still have positive, albeit diminishing associations with equitable vaccination coverage.

Importantly, groups identified via GBTM are unobservable, latent constructs, and observed trajectories are not immutable. Rather, in this analysis, grouping is an aggregated strategy for summarizing trends in vaccination equity across highly heterogenous settings. As such, neither the groups nor their estimated trajectories can predict future equity outcomes in any single country. The number of resultant trajectories and their associated paths will likely evolve as data availability improves for outcome measures, and as additional years’ worth of data are incorporated, particularly with respect to socioeconomic equity. Though integration was not a significant predictor of membership in every group, our analyses do suggest an overall positive association between integrated vaccine delivery and both geographic and socioeconomic vaccination equity in LMICs that have ever received Gavi support. This finding resonates with calls to integrate standalone routine immunization programs into broader systems of care [60, 71–73].

Whether by reducing opportunities for missed vaccination or providing a “one-stop shop” for immunization and other essential health services, integrated vaccine delivery mechanisms may play important roles in shaping health equity [71]. Our finding that integration is most strongly associated with equity improvements in settings characterized by high baseline levels of geographic or socioeconomic inequity aligns with previous studies demonstrating that integrated health platforms can help resource-constrained settings achieve equitable health outcomes [14, 74–76]. Encouragingly, these findings also comport with health worker experiences delivering and patient experiences receiving integrated care. Reporting on focus groups held in four African countries, for example, Ryman et al. note that integration afforded patients greater convenience and access to needed services, reduced transportation times and costs, increased health service utilization and health worker efficiency, and reduced reporting requirements [77]. In this vein, our imputed dataset indicates that countries were likelier to belong to Group 2 (high-stable) if they reported a significant degree of linguistic fractionalization (RR: 2.27 [CI: 1.45, 3.56]), high levels of gender inequality (RR: 3.34 [CI: 1.92, 5.82]), or long distances to the nearest health facility (RR: 1.02 [CI: 1.02, 1.03]). Thus, demand-side interventions targeting these factors might play an important role in improving vaccination equity in LMICs.

This analysis does have several limitations, most of which relate to the quality and availability of our data. Our socioeconomic equity trajectories had wider confidence intervals and fluctuated over the study period, reflecting the paucity of vaccination coverage data disaggregated by wealth quintile. However, given that our results did not meaningfully vary between the imputed and non-imputed datasets, our finding that integration is associated with reductions in inequity likely still holds true.

Additionally, our measures of geographic equity and vaccination coverage were extracted from the WHO-UNICEF Joint Reporting Forms (JRF) on Immunization and WHO-UNICEF Estimates of National Immunization Coverage (WUENIC) at the global level. Previous studies suggest that while the accuracy and completeness of JRFs improves over time and with greater familiarity, critical immunization data are still often missing [78]. In some cases, data provided in response to JRF questions draw from in-country assessments of unknown quality and rigor [79]. WUENIC, in turn, are created from JRF estimates, national administrative coverage estimates (which may be biased by inaccurate numerators or denominators), survey estimates (e.g., Demographic and Health Surveys [DHS] and Multiple Indicator Cluster Surveys [MICS]), and other sources [80]. Furthermore, our measures of socioeconomic equity were calculated from DHS and MICS estimates of vaccination coverage by wealth quintile, which are available only for select years in a handful of countries. Policymakers and decision-makers in LMICs could thus support improved monitoring activities in LMICs by investing in stronger vaccination data collection capacities, systems, and workforces. Additionally, newer measures of equity – such as the Vaccine Economics Research for Sustainability and Equity composite vaccination equity assessment metric – offer more a more sophisticated approach to accounting for the structural factors underpinning observed disparities in coverage between wealth quintiles or other axes of vulnerability [81].

Despite the large number of observations per country and positive model diagnostics, our sample was relatively small, which prevented detection of small and medium associations between integration and equity, especially in our non-imputed dataset. This challenge has also been documented in other GBTM analyses examining country-level phenomena [62]. Loughran and Nagin do report that robust GBTM analyses are possible with as few as 500 study subjects when using Poisson-based models, but whether this threshold applies to other models (e.g., censored normal, binary logit) and the absolute minimum sample size required to apply GBTM remain unknown [82]. Further work is therefore needed to determine how best to model developmental trajectories in inherently small samples. Fortunately, our analysis was still able to capture diverging trends in group membership.

Another limitation relates to the measurement of integration itself. The Working Group’s measure is readily determined from publicly available data collected on a routine basis across all countries; thus, its primary value lies in its convenience and accessibility. Conceptually, however, it is a flawed metric because it does not account for the heterogeneity of integrated health service delivery and structural barriers that might impede a well-integrated system – for example, one with low but near-equal coverage across all services – from raising coverage. Therefore, this measure likely underestimates the number of countries to have demonstrated integration. Furthermore, health system integration may be understood as a continuum ranging from highly vertical programs (e.g., the Global Polio Eradication Initiative) to robust horizontal systems (e.g., those that provide comprehensive primary care) [1]. A quantitative measure of integration, therefore, should ideally exist on a continuous scale, unlike the Working Group’s dichotomous metric. A potential alternative (though, to our knowledge, one that is not publicly reported or readily computable) is the proportion of children under 5 who receive measles vaccinations via primary health programs versus vertical supplemental immunization activities, such as campaigns. Another option, albeit more complex, is to build a composite index that accounts for immunization program financing, workforce structure, modes of delivery, effective coverage, and barriers to vaccine access. Notably, the IA2030 Monitoring and Evaluating Framework includes integrated vaccine delivery as a strategic objective, but many of the prescribed country-level indicators are not routinely made public [83]. This framework does suggest using the composite coverage index (CCI) – a weighted average of eight preventive and curative interventions – but these values are currently only reported for select Countdown 2030 countries and are not reported longitudinally [16, 83]. Furthermore, because CCI is comprised of eight different indicators, we would have likely obtained a very small sample of countries with enough data to compute CCI, which would have further reduced the power of our study and our ability to detect associations between integration and equity.

Despite these limitations, findings from this analysis could nevertheless inform efforts to monitor integrated vaccine delivery in LMICs – an endeavor of particular significance as more LMICs transition away from Gavi support. Our findings could also support future studies of integration and vaccination equity. Follow-on qualitative case studies, for example, might compare and contrast processes of integration between countries in the low-increasing and high-stable geographic equity groups, identify barriers to integrated vaccine delivery among countries following a trajectory marked by high socioeconomic inequity, examine integration and equity trends at subnational levels in large heterogeneous countries, or consider potential demand-side interventions targeting mid-range wealth quintiles in the middle-increasing and -decreasing groups. These follow-on analyses, in turn, could inform decision-making and resource allocation to improve vaccination equity in LMICs. Further work is also needed to explore potential causal mechanisms underpinning the associations identified in this investigation, particularly in the context of universal health coverage provision. In this vein, future analyses might examine whether countries with integrated vaccine delivery programs are more likely to offer health packages or insurance schemes that subsidize the cost of vaccination, thereby incentivizing uptake increasing coverage.

## Conclusion

Amid ongoing calls for universal health coverage and considering the persistent threat of vaccine-preventable diseases in resource-constrained settings, ensuring equitable vaccination remains an urgent public health imperative. The findings from this analysis – which applied GBTM to examine longitudinal trends in geographic and socioeconomic vaccination equity in 78 LMICs – suggest a positive association between integrated vaccine delivery and vaccination equity. Though continued scholarship is needed to further characterize the relationship between integration and health equity, this investigation constitutes a first step toward summarizing these complex phenomena at the country level.

### Supplementary Information

**Additional file 1.**

**Additional file 2.**

**Additional file 3.**

**Additional file 4.**

**Additional file 5.**

**Additional file 6.**

## Data Availability

The datasets generated and/or analyzed in this study are available in the supplementary content of this paper.

## Abbreviations

WHO: World Health Organization
LMIC: Low- and middle-income countries
Gavi: Gavi, the Vaccine Alliance
DTP3: Third dose of the diphtheria, tetanus, toxoid, and pertussis vaccine
MCV1: First dose of measles-containing vaccine
SII: Slope index of inequality
PAB: Protection at birth against neonatal tetanus vaccine
ANC1: Coverage of at least one antenatal care visit
GBTM: Group-based trajectory modeling
BIC: Bayesian information criterion
JRF: WHO-UNICEF Joint Reporting Form
WUENIC: WHO-UNICEF Estimates of National Immunization Coverage at the global level
DHS: Demographic and Health Surveys
MICS: Multiple Indicator Cluster Surveys

## References

[1] Oliveira-Cruz V, Kurowski C, Mills A (2003). Delivery of priority health services: searching for synergies within the vertical versus horizontal debate. J Int Dev..

[2] Razum O, Sridhar D, Jahn A, Zaidi S, Ooms G, Müller O. Polio: from eradication to systematic, sustained control. BMJ Glob Health. 2019;4. Available from: https://gh.bmj.com/content/4/4/e001633. [cited 2022 Feb 19]. 10.1136/bmjgh-2019-001633 PMC6730569 31544903

[3] World Health Organization. Integration of health care delivery: report of a WHO study group. Geneva, Switzerland: World Health Organization; 1996. Report No.: 861. Available from: https://apps.who.int/iris/handle/10665/38408 8669152

[4] World Health Organization. Primary health care. World Health Organ. . Available from: https://www.who.int/health-topics/primary-health-care#tab=tab_1. [cited 2022 Feb 27].

[5] Samb B, Evans T, Dybul M, Atun R, Moatti JP, World Health Organization Maximizing Positive Synergies Collaborative Group (2009). An assessment of interactions between global health initiatives and country health systems. Lancet Lond Engl.

[6] Wyss K, Doumagoum Moto D, Callewaert B (2003). Constraints to scaling-up health related interventions: the case of Chad. Central Africa. J Int Dev..

[7] Seshadri SR (2003). Constraints to scaling-up health programmes: a comparative study of two Indian states. J Int Dev..

[8] Munishi GK (2003). Intervening to address constraints through health sector reforms in Tanzania: some gains and the unfinished business. J Int Dev..

[9] Atun R, de Jongh TE, Secci FV, Ohiri K, Adeyi O, Car J (2011). Integration of priority population, health and nutrition interventions into health systems: systematic review. BMC Public Health..

[10] Wallace A, Dietz V, Cairns KL (2009). Integration of immunization services with other health interventions in the developing world: what works and why? Systematic literature review. Trop Med Int Health TM IH..

[11] Wallace AS, Ryman TK, Dietz V (2012). Experiences integrating delivery of maternal and child health services with childhood immunization programs: systematic review update. J Infect Dis..

[12] Atun R, de Jongh T, Secci FV, Ohiri K, Adeyi O. Clearing the Global Health Fog: A Systematic Review of the Evidence on Integration of Health Systems and Targeted Interventions. Washington, D.C.: World Bank; Report No.: 166. Available from: https://openknowledge.worldbank.org/handle/10986/5946

[13] Atun R, de Jongh T, Secci F, Ohiri K, Adeyi O (2010). A systematic review of the evidence on integration of targeted health interventions into health systems. Health Policy Plan..

[14] Partapuri T, Steinglass R, Sequeira J. Integrated Delivery of Health Services During Outreach Visits: A Literature Review of Program Experience Through a Routine Immunization Lens | The Journal of Infectious Diseases | Oxford Academic. J Infect Dis. 2012 ;205. Available from: https://academic-oup-com.proxy1.library.jhu.edu/jid/article/205/suppl_1/S20/868074?login=true. [cited 2022 Feb 18]. 10.1093/infdis/jir771 PMC3273971 22315382

[15] Montenegro H, Holder R, Ramagem C, Urrutia S, Fabrega R, Tasca R (2011). Combating health care fragmentation through integrated health service delivery networks in the Americas: lessons learned. J Integr Care..

[16] World Health Organization. Immunization Agenda 2030. World Health Organization; 2020. Available from: https://cdn.who.int/media/docs/default-source/immunization/strategy/ia2030/ia2030-draft-4-wha_b8850379-1fce-4847-bfd1-5d2c9d9e32f8.pdf?sfvrsn=5389656e_69&download=true. [cited 2023 Nov 26].

[17] Gavi, the Vaccine Alliance. 2016-2020 Strategy Indicator Definitions. Gavi, the Vaccine Alliance; 2016. Available from: https://www.gavi.org/sites/default/files/document/gavi-2016-2020-strategy-indicator-definitionspdf.pdf

[18] Guignard A, Praet N, Jusot V, Bakker M, Baril L (2019). Introducing new vaccines in low- and middle-income countries: challenges and approaches. Expert Rev Vaccines..

[19] Brearley L, Eggers R, Steinglass R, Vandelaer J (2013). Applying an equity lens in the Decade of Vaccines. Vaccine..

[20] Johri M, Verguet S, Morris SK, Sharma JK, Ram U, Gauvreau C (2016). Adding interventions to mass measles vaccinations in India. Bull World Health Organ..

[21] Goodson JL, Kulkarni MA, Vanden Eng JL, Wannemuehler KA, Cotte AH, Desrochers RE (2012). Improved equity in measles vaccination from integrating insecticide-treated bednets in a vaccination campaign, Madagascar. Trop Med Int Health TM IH..

[22] Dadari I, Higgins-Steele A, Sharkey A, Charlet D, Shahabuddin A, Nandy R (2021). Pro-equity immunization and health systems strengthening strategies in select Gavi-supported countries. Vaccine..

[23] Cash-Gibson L. Integrating Health Services. Geneva, Switzerland: World Health Organization; 2018. Available from: https://www.who.int/docs/default-source/primary-health-care-conference/linkages.pdf

[24] Mitchell S, Andersson N, Ansari NM, Omer K, Soberanis JL, Cockcroft A (2009). Equity and vaccine uptake: a cross-sectional study of measles vaccination in Lasbela District. Pakistan. BMC Int Health Hum Rights..

[25] Sundaram N, Voo TC, Tam CC (2020). Adolescent HPV vaccination: empowerment, equity and ethics. Hum Vaccines Immunother..

[26] Bishai D, Koenig M, Khan MA (2003). Measles vaccination improves the equity of health outcomes: evidence from Bangladesh. Health Econ..

[27] Geweniger A, Abbas KM (2020). Childhood vaccination coverage and equity impact in Ethiopia by socioeconomic, geographic, maternal, and child characteristics. Vaccine..

[28] Helleringer S, Abdelwahab J, Vandenent M (2014). Polio supplementary immunization activities and equity in access to vaccination: evidence from the demographic and health surveys. J Infect Dis..

[29] Bhatia A, Mahmud A, Fuller A, Shin R, Rahman A, Shatil T (2018). The Rohingya in Cox’s Bazar: When the Stateless Seek Refuge. Health Hum Rights..

[30] Colson KE, Zúñiga-Brenes P, Ríos-Zertuche D, Conde-Glez CJ, Gagnier MC, Palmisano E (2015). Comparative Estimates of Crude and Effective Coverage of Measles Immunization in Low-Resource Settings: Findings from Salud Mesoamérica 2015. PLOS ONE..

[31] Arsenault C, Harper S, Nandi A, Rodríguez JMM, Hansen PM, Johri M (2017). An equity dashboard to monitor vaccination coverage. Bull World Health Organ..

[32] World Health Organization. Health Equity Assessment Toolkit: Technical Notes. Geneva, Switzerland: World Health Organization; 2017. Available from: Health Equity Assessment Toolkit Built-in Database Edition Technical Notes

[33] Arambepola R, Yang Y, Hutchinson K, Mwansa FD, Doherty JA, Bwalya F (2021). Using geospatial models to map zero-dose children: factors associated with zero-dose vaccination status before and after a mass measles and rubella vaccination campaign in Southern province. Zambia. BMJ Glob Health..

[34] Immunization Action Coalition. DTaP, Tdap, and Td Catch-up Vaccination Recommendations by Prior Vaccine History and Age. 2020. Available from: https://www.immunize.org/catg.d/p2055.pdf. [cited 2022 Feb 27].

[35] World Health Organization (2017). Diphtheria vaccine: WHO position paper – August 2017. Wkly Epidemiol Rec..

[36] World Health Organization (2017). Measles vaccines: WHO position paper – April 2017. Wkly Epidemiol Rec..

[37] Hagan JE, Greiner A, Luvsansharav U-O, Lake J, Lee C, Pastore R (2017). Use of a Diagonal Approach to Health System Strengthening and Measles Elimination after a Large Nationwide Outbreak in Mongolia. Emerg Infect Dis..

[38] Mmanga K, Mwenyenkulu TE, Nkoka O, Ntenda PAM. Tracking immunization coverage, dropout and equity gaps among children ages 12–23 months in Malawi – bottleneck analysis of the Malawi Demographic and Health Survey. Int Health. 2021;ihab038. 10.1093/inthealth/ihab038 PMC9070459 34153106

[39] Orenstein WA, Hinman A, Nkowane B, Olive JM, Reingold A (2018). Measles and Rubella Global Strategic Plan 2012–2020 midterm review. Vaccine..

[40] Koh HK, Gellin BG (2020). Measles as Metaphor-What Resurgence Means for the Future of Immunization. JAMA..

[41] Measles and Rubella Initiative, American Red Cross, Centers for Disease Control and Prevention, UNICEF, United Nations Foundation, World Health Organization. Measles and Rubella Strategic Framework 2021-2030. Geneva, Switzerland: World Health Organization; Available from: https://apps.who.int/iris/bitstream/handle/10665/339801/9789240015616-eng.pdf?sequence=1

[42] Durrheim DN, Crowcroft NS, Strebel PM (2014). Measles – The epidemiology of elimination. Vaccine..

[43] World Health Organization. Worldwide measles deaths climb 50% from 2016 to 2019 claiming over 207,500 lives in 2019. World Health Organ. 2020 [cited 2022 Feb 27]. Available from: https://www.who.int/news/item/12-11-2020-worldwide-measles-deaths-climb-50-from-2016-to-2019-claiming-over-207-500-lives-in-2019

[44] Guglielmi G. Measles erases immune ‘memory’ for other diseases. Nature. 2019 Oct 31; Available from: https://www.nature.com/articles/d41586-019-03324-7. [cited 2022 Feb 27]. 10.1038/d41586-019-03324-7 33122832

[45] Arsenault C, Johri M, Nandi A, Mendoza Rodríguez JM, Hansen PM, Harper S (2017). Country-level predictors of vaccination coverage and inequalities in Gavi-supported countries. Vaccine..

[46] Arsenault C, Harper S, Nandi A, Mendoza Rodríguez JM, Hansen PM, Johri M (2017). Monitoring equity in vaccination coverage: A systematic analysis of demographic and health surveys from 45 Gavi-supported countries. Vaccine..

[47] Age, criminal careers, and population heterogeneity: specification and estimation of a nonparametric, mixed Poisson model. Criminology. 1993;31:327–62.

[48] Nagin DS, Odgers CL (2010). Group-based trajectory modeling in clinical research. Annu Rev Clin Psychol..

[49] Nagin DS, Odgers CL. Group-based trajectory modeling in developmental science. Handb Dev Res Methods. New York, NY, US: The Guilford Press; 2012. p. 464–80.

[50] Nagin DS (2016). Group-based Trajectory Modeling and Criminal Career Research. J Res Crime Delinquency..

[51] Proulx CM, Ermer AE, Kanter JB (2017). Group-Based Trajectory Modeling of Marital Quality: A Critical Review. J Fam Theory Rev..

[52] Nguefack HLN, Pagé MG, Katz J, Choinière M, Vanasse A, Dorais M (2020). Trajectory Modelling Techniques Useful to Epidemiological Research: A Comparative Narrative Review of Approaches. Clin Epidemiol..

[53] Dugan L, Yang S-M. Introducing Group-Based Trajectory Analysis and Series Hazard Modeling: Two Innovative Methods to Systematically Examine Terrorism Over Time. In: Lum C, Kennedy LW, editors. Evid-Based Counterterrorism Policy. New York, NY: Springer; 2012. p. 113–49. Available from: 10.1007/978-1-4614-0953-3_6. [cited 2023 Oct 16].

[54] Feldman B, Shen J, Chen C, Shi J, Xiang H (2020). Perceived health after adult traumatic brain injury: a Group-Based Trajectory Modeling (GBTM) analysis. Brain Inj..

[55] Ankuda CK, Ornstein KA, Kelley AS (2022). Assessing Health Care Use Trajectories After the Onset of Functional Disability: Application of a Group-Based Trajectory Model. J Gerontol Ser B..

[56] Latent Variable. Encycl Res Des. 2455 Teller Road, Thousand Oaks California 91320 United States: SAGE Publications, Inc.; 2010. Available from: http://methods.sagepub.com/reference/encyc-of-research-design/n213.xml. [cited 2022 Feb 27]

[57] Casey RM. Global Routine Vaccination Coverage, 2015. MMWR Morb Mortal Wkly Rep. 2016;65. Available from: https://www.cdc.gov/mmwr/volumes/65/wr/mm6545a5.htm. [cited 2022 Feb 27]. 10.15585/mmwr.mm6545a5 27855146

[58] The DHS Program. STATcompiler. Available from: https://www.statcompiler.com/en/. [cited 2022 Feb 20].

[59] Weller BE, Bowen NK, Faubert SJ (2020). Latent Class Analysis: A Guide to Best Practice. J Black Psychol..

[60] Sodha SV, Dietz V (2015). Strengthening routine immunization systems to improve global vaccination coverage. Br Med Bull..

[61] Aktaş S, Finch SJ. Project MUSE - Group-Based Trajectory Modeling of Longitudinal International Infant Mortality Rates. Popul Rev. 2014;53. Available from: https://muse.jhu.edu/article/562362/pdf?casa_token=olSfKjRTJIYAAAAA:QuJgrumAecA3bjDHSvNb9bOI9G0yz6Lcdf-zYvfRdz6PvQNy2cX_W_MEP3IWt8xV8O9RN1FCu7k. [cited 2022 Feb 27].

[62] Morris NA, Slocum LA (2012). Estimating Country-Level Terrorism Trends Using Group-Based Trajectory Analyses: Latent Class Growth Analysis and General Mixture Modeling. J Quant Criminol..

[63] Stata Statistical Software: Release 17. College Station, TX, USA: StataCorp; 2021.

[64] Jones BL, Nagin DS. A Stata Plugin for Estimating Group-Based Trajectory Models. 2012. Available from: https://ssrc.indiana.edu/doc/wimdocs/2013-03-29_nagin_trajectory_stata-plugin-info.pdf. [cited 2022 Feb 27]

[65] International Center for Equity in Health. Absolute and relative measures of inequality. Int. Cent. Equity Health Pelotas. Available from: https://equidade.org/siicix. [cited 2022 Feb 28].

[66] Soper D. Free A-priori Sample Size Calculator for Structural Equation Models. 2022. Available from: https://www.danielsoper.com/statcalc/calculator.aspx?id=89. [cited 2022 Feb 28]

[67] Cohen J. Statistical Power Analysis for the Behavioral Sciences. 2nd ed. New York, NY: Lawrence Erlbaum Associates; 1988. Available from: https://www.taylorfrancis.com/books/mono/10.4324/9780203771587/statistical-power-analysis-behavioral-sciences-jacob-cohen. [cited 2022 Feb 28]

[68] Christopher Westland J (2010). Lower bounds on sample size in structural equation modeling. Electron Commer Res Appl..

[69] Nagin DS. Group-Based Modeling of Development. 1st ed. Cambridge, MA, USA: Harvard University Press; 2005. Available from: https://www.hup.harvard.edu/catalog.php?isbn=9780674016866. [cited 2022 Feb 27]

[70] Brownwright TK, Dodson ZM, van Panhuis WG (2017). Spatial clustering of measles vaccination coverage among children in sub-Saharan Africa. BMC Public Health..

[71] Mantel C, Cherian T (2020). New immunization strategies: adapting to global challenges. Bundesgesundheitsblatt Gesundheitsforschung Gesundheitsschutz..

[72] Mihigo R, Anya B, Okeibunor J, Ajibola S, Boakye-Agyemang C, Muzenda L (2015). African vaccination week as a vehicle for integrated health service delivery. BMC Health Serv Res..

[73] Ropero-Álvarez AM, Kurtis HJ, Danovaro-Holliday MC, Ruiz-Matus C, Tambini G (2012). Vaccination Week in the Americas: an opportunity to integrate other health services with immunization. J Infect Dis..

[74] Lava JB, Claro V de, Quiñon MS, Labis R, Marcelo W, Lucero MA, et al. Integrating COVID-19 Vaccination in Primary Care Service Delivery: Insights From Implementation Research in the Philippines. Glob Health Sci Pract. 2023; Available from: https://www.ghspjournal.org/content/early/2023/11/09/GHSP-D-23-00202.1. [cited 2023 Nov 30] 10.9745/GHSP-D-23-00202 PMC10948126 38378272

[75] O’Brien KL, Lemango E, Nandy R, Lindstrand A. The immunization Agenda 2030: A vision of global impact, reaching all, grounded in the realities of a changing world. Vaccine. 2022; Available from: https://www.ncbi.nlm.nih.gov/pmc/articles/PMC9754085/. [cited 2023 Nov 30] 10.1016/j.vaccine.2022.02.073 PMC9754085 36528445

[76] Portnoy A, Jit M, Helleringer S, Verguet S (2020). Comparative Distributional Impact of Routine Immunization and Supplementary Immunization Activities in Delivery of Measles Vaccine in Low- and Middle-Income Countries. Value Health..

[77] Ryman TK, Wallace A, Mihigo R, Richards P, Schlanger K, Cappelier K (2012). Community and Health Worker Perceptions and Preferences Regarding Integration of Other Health Services With Routine Vaccinations: Four Case Studies. J Infect Dis..

[78] Ortiz JR, Perut M, Dumolard L, Wijesinghe PR, Jorgensen P, Ropero AM (2016). A global review of national influenza immunization policies: Analysis of the 2014 WHO/UNICEF Joint Reporting Form on immunization. Vaccine..

[79] Kulkarni S, Harvey B, Prybylski D, Jalloh MF (2021). Trends in classifying vaccine hesitancy reasons reported in the WHO/UNICEF Joint Reporting Form, 2014–2017: Use and comparability of the Vaccine Hesitancy Matrix. Hum Vaccines Immunother..

[80] Pan American Health Organization. Frequent Asked Questions (FAQs): WHO-UNICEF Joint Reporting Form on Immunization (JRF) and Estimates of National Immunization Coverage (WUENIC). 2020. Available from: https://iris.paho.org/bitstream/handle/10665.2/52625/PAHOFPLIM200015_eng.pdf?sequence=1&isAllowed=y

[81] Patenaude B, Odihi D, Sriudomporn S, Mak J, Watts E, de Broucker G. A Standardized Approach for Measuring Multi-Dimensional Equity in Vaccination Coverage, Cost-of-Illness, and Health Outcomes: Evidence from the Vaccine Economics Research for Sustainability & Equity (VERSE) Project. Rochester, NY: Social Science Research Network; 2021 Oct. Report No.: ID 3945450. Available from: https://papers.ssrn.com/abstract=3945450 10.1016/j.socscimed.2022.114979 PMC9127392 35462106

[82] Loughran T, Nagin DS (2006). Finite Sample Effects in Group-Based Trajectory Models. Sociol Methods Res..

[83] World Health Organization. IA2030 Monitoring and Evaluation Framework. 2021. Available from: https://www.immunizationagenda2030.org/images/documents/IA2030_Annex_FrameworkForActionv04.pdf. [cited 2023 Nov 28]

