# Supplementary material for: Group-based trajectory models of integrated vaccine delivery and equity in low- and middle-income countries
Source: Int J Equity Health. 2024 Jan 9;23:5. doi: 10.1186/s12939-023-02088-x (PMC10775446; doi:10.1186/s12939-023-02088-x)
Supplement: Supplementary file 2 — Additional file 2. [file 12939_2023_2088_MOESM2_ESM.docx]

| **Appendix B**  **Group-Based Trajectory Model Data Glossary** | | | |
| --- | --- | --- | --- |
| **Descriptive Indicators** | **Description** | **Type** | **Data Source** |
| **Alpha-3 code** | Standardized 3-letter country codes designated by the International Organization for Standardization | Alphanumeric | [ISO Alpha-3 codes](https://www.iso.org/obp/ui/#search) |
| **Independent Variables** | **Description** | **Type** | **Data Source** |
| **Antenatal care coverage** | National coverage of at least one antenatal care visit (%) | Continuous | [Demographic and Health Surveys (DHS)](https://www.statcompiler.com/en/) & [Multiple Indicator Cluster Surveys (MICS)](https://mics.unicef.org/surveys) |
| **Control of corruption** | Measurement of perceptions of the extent to which public power is exercised for private gain, including both petty and grand forms of corruption, as well as "capture" of the state by elites and private interests | Continuous | [Worldwide Governance Indicators](https://info.worldbank.org/governance/wgi/Home/Documents) |
| **Diphtheria-tetanus-pertussis vaccination coverage** | National coverage of the third dose of diphtheria-tetanus-pertussis-containing vaccine (%) | Continuous | [WHO-UNICEF Joint Reporting Forms on Immunization](https://immunizationdata.who.int/) |
| **Distance to the nearest health facility** | Proportion of the population more than 60 minutes away (by walking) from the nearest health facility (%) | Continuous | [Weiss et al. (“Global maps of travel time to healthcare facilities,” *Nature Medicine*)](https://www.nature.com/articles/s41591-020-1059-1) |
| **External resources for health per capita** | External resources for health per capita (expressed in current international dollars, purchasing power parity) | Continuous | [WHO Global Health Expenditure Database](https://apps.who.int/nha/database/Select/Indicators/en) |
| **Independent Variables** | **Description** | **Type** | **Data Source** |
| **Gender**  **inequality**  **index** | A composite measure reflecting inequality in achievement between women and men in three dimensions: reproductive health, empowerment and the labor market | Continuous | [United Nations Development Programme Human Development Reports](http://hdr.undp.org/en/indicators/68606) |
| **Government effectiveness** | Measurement of perceptions of the quality of public services, the quality of the civil service and the degree of its independence from political pressures, the quality of policy formulation and implementation, and the credibility of the  government's commitment to such policies | Continuous | [Worldwide Governance Indicators](https://info.worldbank.org/governance/wgi/Home/Documents) |
| **Government expenditure on health per capita** | Government health expenditures (in current international dollars, purchasing power parity) | Continuous | [WHO Global Health Expenditure Database](https://apps.who.int/nha/database/Select/Indicators/en) |
| **Income Level** | World Bank Region | Alphanumeric | - [World Bank Country and Lending Groups](https://datahelpdesk.worldbank.org/knowledgebase/articles/906519-world-bank-country-and-lending-groups) |
| **Integrated**  **vaccine delivery** | This analysis utilizes the dichotomous measure developed by the WHO SAGE working group and adopted by Gavi, wherein countries are considered to have achieved integration only if national co-coverage levels of MCV1, DTP3, PAB, and ANC1 exceed 70%, and are within 10 percentage points of one another. | Dichotomous | - [WHO-UNICEF Joint Reporting Forms on Immunization](https://immunizationdata.who.int/) - [WHO Global Health Observatory](https://www.who.int/data/gho/data/indicators/indicator-details/GHO/neonates-protected-at-birth-against-neonatal-tetanus-(pab)-(-)) - [Demographic and Health Surveys (DHS)](https://www.statcompiler.com/en/) & [Multiple Indicator Cluster Surveys (MICS)](https://mics.unicef.org/surveys) |
| **Independent Variables** | **Description** | **Type** | **Data Source** |
| **Land area** | National land area (square kilometers) | Continuous | [World Bank Open Data](https://data.worldbank.org/indicator/AG.LND.TOTL.K2) |
| **Linguistic fractionalization** | A measure of linguistic diversity within a given country | Continuous | [Alesina et al. (“Fractionalization,” *Journal of Economic Growth*)](https://scholar.harvard.edu/files/alesina/files/fractionalization.pdf) |
| **Measles vaccination coverage** | National coverage of the first dose of measles-containing vaccine (%) | Continuous | [WHO-UNICEF Joint Reporting Forms on Immunization](https://apps.who.int/immunization_monitoring/globalsummary/indicators) |
| **Measles vaccination coverage in the wealthiest quintile** | National coverage of the first dose of measles-containing vaccine in the wealthiest quintile (%) | Continuous | [Demographic and Health Surveys (DHS)](https://www.statcompiler.com/en/) & [Multiple Indicator Cluster Surveys (MICS)](https://mics.unicef.org/surveys) |
| **Measles vaccination coverage in the poorest quintile** | National coverage of the first dose of measles-containing vaccine in the poorest quintile (%) | Continuous | [Demographic and Health Surveys (DHS)](https://www.statcompiler.com/en/) & [Multiple Indicator Cluster Surveys (MICS)](https://mics.unicef.org/surveys) |
| **Out-of-pocket expenditure on health** | Share of out-of-pocket expenditure on health (as a percentage of total health expenditure) | Continuous | [WHO Global Health Expenditure Database](https://apps.who.int/nha/database/Select/Indicators/en) |
| **Political stability and absence of violence and terrorism** | Measurement of the likelihood of political instability and/or politically motivated violence, including terrorism | Continuous | [Worldwide Governance Indicators](https://info.worldbank.org/governance/wgi/Home/Documents) |
| **Protection at birth against neonatal tetanus** | National proportion of neonates protected at birth against neonatal tetanus via maternal immunization (%) | Continuous | [WHO Global Health Observatory](https://www.who.int/data/gho/data/indicators/indicator-details/GHO/neonates-protected-at-birth-against-neonatal-tetanus-(pab)-(-)) |
| **Region** | Current income level (as of 2022, per the World Bank) | Alphanumeric | [World Bank Country and Lending Groups](https://datahelpdesk.worldbank.org/knowledgebase/articles/906519-world-bank-country-and-lending-groups) |
| **Women with completed primary education** | National proportion of women who have completed their primary education | Continuous | [World Development Indicator Database](https://www.google.com/url?q=https://data.worldbank.org/indicator/SE.PRM.CMPT.FE.ZS&sa=D&source=docs&ust=1639413633784000&usg=AOvVaw0cZvxPIP02TvGXlL_QCyJf) |
| **Dependent Variables** | **Description** | **Type** | **Data Source** |
| **Geographic vaccination equity** | Proportion of districts reporting ≥ 80% coverage of measles-containing vaccine | Continuous | [WHO-UNICEF Joint Reporting Forms on Immunization](https://immunizationdata.who.int/) |
| **Socioeconomic vaccination equity** | Slope index of inequality (SII) based on MCV1 coverage disaggregated by wealth quintile | Continuous | [Demographic and Health Surveys (DHS)](https://www.statcompiler.com/en/) & [Multiple Indicator Cluster Surveys (MICS)](https://mics.unicef.org/surveys) |
