# Supplementary material for: Group-based trajectory models of integrated vaccine delivery and equity in low- and middle-income countries
Source: Int J Equity Health. 2024 Jan 9;23:5. doi: 10.1186/s12939-023-02088-x (PMC10775446; doi:10.1186/s12939-023-02088-x)
Supplement: Supplementary file 3 — Additional file 3. [file 12939_2023_2088_MOESM3_ESM.pdf]

```
  ___  ____  ____  ____  ____ (R)
 /__    /   ____/   /   ____/
___/   /   /___/   /   /___/   15.1
  Statistics/Data Analysis
```

Copyright 1985-2017 StataCorp LLC

StataCorp
4905 Lakeway Drive
College Station, Texas 77845 USA
800-STATA-PC   http://www.stata.com
979-696-4600   stata@stata.com
979-696-4601 (fax)

Notes:
      1. Unicode is supported; see help unicode advice.

```
. doedit "C:\

. do "C:\

. *Import dataset.*
.
. import delimited "C:\
(21 vars, 1,326 obs)

.
. *Assign labels to each variable in the dataset.*
.
. label variable alpha3 "Alpha-3 Code"

. label variable q5coverage "MCV1 Coverage in the Wealthiest Quintile"

. label variable q1coverage "MCV1 Coverage in the Poorest Quintile"

. label variable geoequity "Geographic Equity"

. label variable dtp3 "DTP3 Coverage"

. label variable mcv1 "MCV1 Coverage"

. label variable anc1 "Antenatal Care Coverage (1+ Visits)"

. label variable pab "Protection at Birth Against Neonatal Tetanus"

. label variable femeduc "Female Primary Education Completed"

. label variable polstability "Political Stability"

. label variable goveffect "Government Effectiveness"
```

```
20 . label variable corrupt "Corruption"

21 . label variable gii "Gender Inequality Index"

22 . label variable oopexp "Out-of-Pocket Health Expenditures"

23 . label variable govexp "Domestic Government Health Expenditures"

24 . label variable exthlth "External Health Spending"

25 . label variable land "Land Area"

26 . label variable lingfrac "Linguistic Fractionalization"

27 . label variable distance "Distance to Nearest Health Facility (Walking, 60+ Minutes)"

28 .
29 . *****************************************************
30 . ***Perform multiple imputation to handle missing data***
31 . *****************************************************
32 .
33 . *First, identify missing values in the dataset. Install the "mdesc" package if needed.*
34 .
35 . mdesc q5coverage q1coverage geoequity dtp3 mcv1 anc1 pab femeduc polstability goveffect corrupt
   > rac distance
```

Variable	Missing	Total	Percent Missing
q5coverage	1,110	1,326	83.71
q1coverage	1,113	1,326	83.94
geoequity	173	1,326	13.05
dtp3	10	1,326	0.75
mcv1	8	1,326	0.60
anc1	1,008	1,326	76.02
pab	233	1,326	17.57
femeduc	537	1,326	40.50
polstability	8	1,326	0.60
goveffect	8	1,326	0.60
corrupt	7	1,326	0.53
gii	649	1,326	48.94
oopexp	140	1,326	10.56
govexp	140	1,326	10.56
exthlth	140	1,326	10.56
land	8	1,326	0.60
lingfrac	51	1,326	3.85
distance	24	1,326	1.81

```
36 .
37 . *Create a pairwise correlation matrix to identify potential auxiliary variables.*
38 .
39 . pwcorr q5coverage q1coverage geoequity dtp3 mcv1 anc1 pab femeduc polstability goveffect corrup
   > frac distance
```

	q5cove~e	q1cove~e	geoequ~y	dtp3	mcv1	anc1	pab
q5coverage	**1.0000**						
q1coverage	**0.6281**	**1.0000**					
geoequity	**0.2300**	**0.3073**	**1.0000**				
dtp3	**0.5752**	**0.8465**	**0.4849**	**1.0000**			
mcv1	**0.5457**	**0.8753**	**0.5076**	**0.9327**	**1.0000**		
anc1	**0.4729**	**0.5951**	**0.3168**	**0.6588**	**0.6774**	**1.0000**	
pab	**0.4227**	**0.5877**	**0.4671**	**0.5965**	**0.5821**	**0.4524**	**1.0000**
femeduc	**0.3703**	**0.5205**	**0.3353**	**0.5543**	**0.6116**	**0.4605**	**0.3549**
polstability	**0.2494**	**0.4526**	**0.2423**	**0.5301**	**0.4956**	**0.5417**	**0.3489**
goveffect	**0.3012**	**0.3078**	**0.2922**	**0.4600**	**0.4626**	**0.3677**	**0.3302**
corrupt	**0.2340**	**0.2832**	**0.1959**	**0.4221**	**0.4001**	**0.3809**	**0.3100**
gii	**0.0286**	**0.1052**	**0.0784**	**0.1231**	**0.1048**	**0.0473**	**-0.0129**
oopexp	**-0.2164**	**-0.3076**	**-0.0370**	**-0.1919**	**-0.1982**	**-0.3969**	**-0.0332**
govexp	**0.0955**	**0.3204**	**0.1907**	**0.2190**	**0.2678**	**0.2461**	**0.1847**
exthlth	**0.1314**	**0.2588**	**0.0542**	**0.1583**	**0.1176**	**0.1924**	**0.1691**
land	**-0.0590**	**-0.3470**	**0.0120**	**-0.0627**	**-0.0418**	**0.0529**	**-0.2108**
lingfrac	**-0.0580**	**-0.2804**	**-0.1435**	**-0.2875**	**-0.3367**	**-0.1515**	**-0.0855**
distance	**-0.0729**	**0.0351**	**0.0133**	**0.0245**	**0.0984**	**-0.1866**	**-0.0842**

	femeduc	polsta~y	goveff~t	corrupt	gii	oopexp	govexp
femeduc	**1.0000**						
polstability	**0.3344**	**1.0000**					
goveffect	**0.4249**	**0.4793**	**1.0000**				
corrupt	**0.2481**	**0.5881**	**0.7399**	**1.0000**			
gii	**-0.0221**	**-0.0051**	**0.0268**	**0.0044**	**1.0000**		
oopexp	**-0.0728**	**-0.4911**	**-0.2437**	**-0.4723**	**-0.0045**	**1.0000**	
govexp	**0.2737**	**0.2610**	**0.2712**	**0.3047**	**-0.1186**	**-0.2392**	**1.0000**
exthlth	**0.0355**	**0.2336**	**-0.0415**	**0.1797**	**-0.0721**	**-0.4768**	**-0.1415**
land	**-0.1103**	**-0.1418**	**0.1638**	**-0.0273**	**0.1948**	**0.0910**	**0.0105**
lingfrac	**-0.4576**	**-0.1729**	**-0.0583**	**-0.0730**	**0.0277**	**0.0683**	**-0.3200**
distance	**0.2705**	**0.0602**	**-0.0898**	**-0.0958**	**-0.0326**	**0.0677**	**0.2000**

	exthlth	land	lingfrac	distance
exthlth	**1.0000**			
land	**-0.2399**	**1.0000**		
lingfrac	**0.0890**	**0.0268**	**1.0000**	
distance	**-0.2347**	**0.0031**	**-0.3394**	**1.0000**

```
40 .
41 . *Set the data for multiple imputation. The "mi set mlong" command generates three additional va
   > the imputed datasets and values*
42 .
43 . mi set mlong

44 .
45 . *Summarize missing values in the dataset.*
46 .
47 . mi misstable summarize q5coverage q1coverage geoequity dtp3 mcv1 anc1 pab femeduc polstability 
   > xthlth land lingfrac distance
```

				Obs<.		
Variable	Obs=.	Obs>.	Obs<.	Unique values	Min	Max
q5coverage	**1,110**		**216**	153	20	100
q1coverage	**1,113**		**213**	182	8.2	100
geoequity	**173**		**1,153**	102	0	104
dtp3	**10**		**1,316**	75	19	99
mcv1	**8**		**1,318**	72	16	99
anc1	**1,008**		**318**	204	16.1	100
pab	**233**		**1,093**	63	31	99

femeduc	**537**	**789**	>500	16.65596	125.6158
polstability	**8**	**1,318**	357	-3.31	1.42
goveffect	**8**	**1,318**	251	-2.48	.83
corrupt	**7**	**1,319**	235	-1.87	1.65
gii	**649**	**677**	419	.038	.776
oopexp	**140**	**1,186**	85	0	86
govexp	**140**	**1,186**	286	1	2873
exthlth	**140**	**1,186**	102	0	189
land	**8**	**1,318**	175	810	9424703
lingfrac	**51**	**1,275**	74	0	.9227
distance	**24**	**1,302**	76	6.8	91.9

```
48 .
49 . *Summarize missing patterns in the dataset.*
50 .
51 . mi misstable patterns q5coverage q1coverage geoequity dtp3 mcv1 anc1 pab femeduc polstability g
   > thlth land lingfrac distance
```

Missing-value patterns
(1 means complete)

Percent	Pattern 1	2	3	4	5	6	7	8	9	10	11	12	13	14	15	16
**4%**	1	1	1	1	1	1	1	1	1	1	1	1	1	1	1	1
	1	1														
**15**	1	1	1	1	1	1	1	1	1	1	1	1	1	1	0	0
	0	0														
**15**	1	1	1	1	1	1	1	1	1	1	1	1	1	1	1	0
	0	0														
**10**	1	1	1	1	1	1	1	1	1	1	1	1	1	0	1	0
	0	0														
**7**	1	1	1	1	1	1	1	1	1	1	1	1	1	0	0	0
	0	0														
**3**	1	1	1	1	1	1	1	1	1	1	1	1	1	1	0	1
	1	1														
**3**	1	1	1	1	1	1	1	1	0	0	0	1	1	0	1	0
	0	0														
**3**	1	1	1	1	1	1	1	1	1	1	1	1	0	1	1	0
	0	0														
**3**	1	1	1	1	1	1	1	1	1	1	1	0	1	1	0	0
	0	0														
**3**	1	1	1	1	1	1	1	1	1	1	1	1	0	1	0	0
	0	0														
**3**	1	1	1	1	1	1	1	1	1	1	1	1	1	0	1	1
	1	1														
**2**	1	1	1	1	1	1	1	1	1	1	1	1	0	0	0	0
	0	0														
**2**	1	1	1	1	1	1	1	1	1	1	1	0	0	1	0	0
	0	0														
**2**	1	1	1	1	1	1	1	1	1	1	1	1	1	1	1	1

	0	0														
**2**	1	1	1	1	1	1	1	1	1	1	1	0	1	0	0	0
	0	0														
**1**	1	1	1	1	1	1	1	1	0	0	0	1	1	0	0	0
	0	0														
**1**	1	1	1	1	1	1	1	1	1	1	1	1	1	0	0	1
	1	1														
**1**	1	1	1	1	1	1	1	1	0	0	0	1	1	1	1	0
	0	0														
**1**	1	1	1	1	1	1	1	1	1	1	1	1	0	0	1	0
	0	0														
<1	1	1	1	1	1	1	1	1	1	1	1	1	0	1	0	1
	0	0														
<1	1	1	1	1	1	1	1	1	1	1	1	1	1	0	0	1
	0	0														
<1	1	1	1	1	1	1	1	1	1	1	1	1	1	1	0	1
	0	0														
<1	1	1	1	1	1	1	1	1	1	1	1	1	1	0	1	1
	0	0														
<1	1	1	1	1	1	1	1	0	1	1	1	1	1	0	1	0
	0	0														
<1	1	1	1	1	1	1	1	1	1	1	1	0	0	0	0	0
	0	0														
<1	1	1	1	1	1	1	1	1	1	1	1	1	0	0	1	1
	0	0														
<1	1	1	1	1	1	1	1	1	1	1	1	1	0	1	1	1
	0	0														
<1	1	1	1	1	1	1	1	1	1	1	1	1	0	1	1	1
	1	1														
<1	1	1	1	1	1	1	1	1	0	0	0	0	1	0	0	0
	0	0														
<1	1	1	1	1	1	1	1	1	1	1	1	0	1	1	1	0
	0	0														
<1	0	0	0	0	0	0	0	0	0	0	0	0	0	0	0	0
	0	0														
<1	1	1	1	1	1	1	0	1	1	1	1	1	1	1	1	0
	0	0														
<1	1	1	1	1	1	1	1	1	1	1	1	0	1	1	0	1
	0	0														
<1	1	1	1	1	1	1	1	0	1	1	1	1	1	0	0	0
	0	0														
<1	1	1	1	1	1	1	1	1	0	0	0	1	1	0	1	1
	1	1														

<1	1	1	1	1	1	1	1	1	1	1	1	0	0	1	0	1	0	0
<1	1	1	1	1	1	1	1	1	1	1	1	0	1	0	1	0	0	0
<1	1	1	1	1	1	1	1	0	0	0	0	1	1	0	1	0	0	0
<1	1	1	1	1	1	1	1	0	1	1	1	1	1	1	1	0	0	0
<1	1	1	1	1	1	1	1	1	0	0	0	1	0	1	1	0	0	0
<1	1	1	1	1	1	1	1	1	0	0	0	1	1	1	0	0	0	0
<1	1	1	1	1	1	1	1	1	1	1	1	1	1	1	1	0	1	1
<1	1	1	1	1	1	1	0	1	1	1	1	0	1	0	0	0	0	0
<1	1	1	1	1	1	1	1	1	1	1	1	0	0	1	1	0	0	0
<1	1	1	1	1	1	1	1	1	1	1	1	0	1	0	0	1	1	1
<1	1	1	1	1	1	1	1	1	1	1	1	0	1	1	0	1	1	1
<1	1	1	1	1	1	1	1	1	1	1	1	1	0	1	0	1	1	1
<1	1	1	1	1	1	1	1	0	1	1	1	1	1	0	1	1	1	1
<1	1	1	1	1	1	1	1	1	0	0	0	1	1	0	0	1	1	1
<1	1	1	1	1	1	1	1	1	0	0	0	1	1	0	1	1	0	0
<1	1	1	1	1	1	1	1	1	0	0	0	1	1	1	1	1	1	1
<1	1	1	1	1	1	1	1	1	1	1	1	0	1	0	0	1	0	0
<1	1	1	1	1	1	1	1	1	1	1	1	1	0	0	0	1	0	0
<1	1	1	1	1	1	1	1	1	1	1	1	1	1	1	0	0	1	1
<1	1	1	1	1	1	1	0	1	1	1	1	1	1	0	0	0	0	0
<1	1	1	1	1	1	1	1	0	0	0	0	1	1	1	1	0	0	0
<1	1	1	1	1	1	1	1	0	1	1	1	0	1	1	0	0	0	0

<1	1	1	1	1	1	1	1	1	0	0	0	1	0	0	1	0	0	0
<1	1	1	1	1	1	1	1	1	1	1	1	0	0	0	0	1	0	0
<1	1	1	1	1	1	1	1	1	1	1	1	0	1	0	1	0	1	0
<1	1	1	1	1	1	1	1	1	1	1	1	0	1	1	1	1	1	1
<1	1	1	1	1	1	1	1	1	1	1	1	1	0	0	0	1	1	1
<1	1	1	1	1	1	1	1	1	1	1	1	1	0	0	1	1	1	1
<1	1	0	0	0	0	0	1	0	0	0	0	0	0	0	1	1	1	1
<1	1	1	1	1	1	0	1	1	1	1	1	0	0	1	0	1	0	0
<1	1	1	1	1	1	0	1	1	1	1	1	1	1	0	0	0	1	1
<1	1	1	1	1	1	1	0	1	0	0	0	1	1	0	1	0	0	0
<1	1	1	1	1	1	1	0	1	1	1	1	0	1	0	0	1	0	0
<1	1	1	1	1	1	1	0	1	1	1	1	1	1	0	1	0	0	0
<1	1	1	1	1	1	1	0	1	1	1	1	1	1	1	1	1	0	0
<1	1	1	1	1	1	1	1	0	0	0	0	0	1	0	1	0	0	0
<1	1	1	1	1	1	1	1	0	0	0	0	1	1	0	1	1	0	0
<1	1	1	1	1	1	1	1	0	1	1	1	0	1	0	0	0	0	0
<1	1	1	1	1	1	1	1	0	1	1	1	0	1	0	1	0	0	0
<1	1	1	1	1	1	1	1	0	1	1	1	0	1	1	1	1	1	1
<1	1	1	1	1	1	1	1	0	1	1	1	1	1	0	0	1	1	1
<1	1	1	1	1	1	1	1	0	1	1	1	1	1	0	1	1	0	0
<1	1	1	1	1	1	1	1	0	1	1	1	1	1	1	0	0	0	0
<1	1	1	1	1	1	1	1	0	1	1	1	1	1	1	0	1		

																	1	1
<1	1	1	1	1	1	1	1	0	1	1	1	1	1	1	1	1	0	0
<1	1	1	1	1	1	1	1	0	1	1	1	1	1	1	1	1	1	1
<1	1	1	1	1	1	1	1	1	0	0	0	0	0	0	0	0	0	0
<1	1	1	1	1	1	1	1	1	0	0	0	0	0	0	1	0	0	0
<1	1	1	1	1	1	1	1	1	0	0	0	0	1	0	1	0	0	0
<1	1	1	1	1	1	1	1	1	0	0	0	0	1	1	0	0	0	0
<1	1	1	1	1	1	1	1	1	0	0	0	1	0	0	0	0	0	0
<1	1	1	1	1	1	1	1	1	0	0	0	1	0	0	0	1	0	0
<1	1	1	1	1	1	1	1	1	0	0	0	1	0	1	0	0	0	0
<1	1	1	1	1	1	1	1	1	0	0	0	1	0	1	1	1	0	0
<1	1	1	1	1	1	1	1	1	0	0	0	1	1	0	0	0	1	1
<1	1	1	1	1	1	1	1	1	0	0	0	1	1	0	0	1	0	0
<1	1	1	1	1	1	1	1	1	0	0	0	1	1	1	0	1	0	0
<1	1	1	1	1	1	1	1	1	1	1	1	0	0	0	0	1	1	1
<1	1	1	1	1	1	1	1	1	1	1	1	0	0	0	1	0	0	0
<1	1	1	1	1	1	1	1	1	1	1	1	0	0	0	1	1	1	1
<1	1	1	1	1	1	1	1	1	1	1	1	0	0	1	0	1	1	1
<1	1	1	1	1	1	1	1	1	1	1	1	0	1	0	0	0	1	1
<1	1	1	1	1	1	1	1	1	1	1	1	0	1	0	1	1	0	0
<1	1	1	1	1	1	1	1	1	1	1	1	0	1	1	1	0	1	0
<1	1	1	1	1	1	1	1	1	1	1	1	1	1	0	1	0	1	1

```
    100%  |

  Variables are
      Row 1:   (1) corrupt  (2) goveffect  (3) land  (4) mcv1  (5) polstability  (6) dtp3  (7) di
               (10) govexp  (11) oopexp  (12) geoequity  (13) pab  (14) femeduc  (15) gii  (16) a
      Row 2:   (1) q5coverage  (2) q1coverage

52 .
53 . *Register the variables to be imputed.*
54 .
55 . mi register imputed q5coverage q1coverage geoequity anc1 pab
   (1157 m=0 obs. now marked as incomplete)

56 .
57 . *Specify the imputed model.*
58 .
59 . mi impute mvn geoequity anc1 pab = mcv1, add(10) rseed(54321) force

   Performing EM optimization:
   note: 37 observations omitted from EM estimation because of all imputation variables missing
     observed log likelihood = -8367.4122 at iteration 40

   Performing MCMC data augmentation ...

   Multivariate imputation                      Imputations =       10
   Multivariate normal regression                     added =       10
   Imputed: m=1 through m=10                        updated =        0

   Prior: uniform                                Iterations =     1000
                                                    burn-in =      100
                                                    between =      100
```

	Observations per *m*			
Variable	Complete	Incomplete	Imputed	Total
geoequity	1153	173	165	1326
anc1	318	1008	1001	1326
pab	1093	233	225	1326

```
   (complete + incomplete = total; imputed is the minimum across m
    of the number of filled-in observations.)

   Note: Right-hand-side variables (or weights) have missing values;
         model parameters estimated using listwise deletion.

60 .
61 . *Specify a linear regression model to estimate the missing values.*
62 .
63 . mi estimate: regress q5coverage q1coverage geoequity anc1 pab

   Multiple-imputation estimates                    Imputations       =         10
   Linear regression                                Number of obs     =        212
                                                    Average RVI       =     0.1211
                                                    Largest FMI       =     0.2395
                                                    Complete DF       =        207
   DF adjustment:   Small sample                    DF:     min       =      82.41
                                                            avg       =     135.34
                                                            max       =     186.54
   Model F test:       Equal FMI                    F(   4,  181.6)   =      29.27
   Within VCE type:          OLS                    Prob > F          =     0.0000
```

q5coverage	Coef.	Std. Err.	t	P>|t|	[95% Conf.	Interval]
q1coverage	**.2735273**	**.0421289**	**6.49**	**0.000**	**.190417**	**.3566377**
geoequity	**.0000196**	**.0226001**	**0.00**	**0.999**	**-.044601**	**.0446402**
anc1	**.0722887**	**.0531523**	**1.36**	**0.178**	**-.0334402**	**.1780176**
pab	**.1054068**	**.0831516**	**1.27**	**0.208**	**-.0598366**	**.2706501**
_cons	**51.2888**	**5.458233**	**9.40**	**0.000**	**40.50594**	**62.07167**

```
. 
. *Perform a diagnostic check to assess how well the imputation was performed.*
. 
. mi estimate, vartable dftable
```

Multiple-imputation estimates Imputations = **10**
Linear regression

Variance information

	Imputation variance					Relative
	Within	Between	Total	RVI	FMI	efficiency
q1coverage	**.001692**	**.000076**	**.001775**	**.049141**	**.047829**	**.99524**
geoequity	**.00047**	**.000037**	**.000511**	**.087544**	**.082642**	**.991804**
anc1	**.002179**	**.000587**	**.002825**	**.296505**	**.239515**	**.976609**
pab	**.005428**	**.001351**	**.006914**	**.27388**	**.224846**	**.97801**
_cons	**26.8366**	**2.68696**	**29.7923**	**.110135**	**.102158**	**.989887**

Multiple-imputation estimates		Imputations	=	**10**
Linear regression		Number of obs	=	**212**
		Average RVI	=	**0.1211**
		Largest FMI	=	**0.2395**
		Complete DF	=	**207**
DF adjustment:	**Small sample**	DF: min	=	**82.41**
		avg	=	**135.34**
		max	=	**186.54**
Model F test:	**Equal FMI**	F(**4**, **181.6**)	=	**29.27**
Within VCE type:	**OLS**	Prob > F	=	**0.0000**

q5coverage	Coef.	Std. Err.	t	P>|t|	DF	% Increase Std. Err.
q1coverage	**.2735273**	**.0421289**	**6.49**	**0.000**	**186.5**	**2.43**
geoequity	**.0000196**	**.0226001**	**0.00**	**0.999**	**166.0**	**4.29**
anc1	**.0722887**	**.0531523**	**1.36**	**0.178**	**82.4**	**13.86**
pab	**.1054068**	**.0831516**	**1.27**	**0.208**	**88.1**	**12.87**
_cons	**51.2888**	**5.458233**	**9.40**	**0.000**	**153.7**	**5.36**

```
68 .
69 . ***************************************************************************************
70 . ***Creating new variables for socioeconomic vaccine equity & integrated vaccine delivery.***
71 . ***************************************************************************************
72 .
73 . *Create and label a new variable, "ceqr," representing the ratio of MCV1 coverage in the wealth
74 .
75 . generate ceqr = q5coverage/q1coverage
   (12,243 missing values generated)

76 . label variable ceqr "Socioeconomic Equity"

77 .
78 . *Create and label a new variable, integration, representing integrated vaccine delivery.*
79 .
80 . generate integration = 0

81 . label variable integration "Integrated Vaccine Delivery"

82 .
83 . *Create new variables representing all possible absolute differences between MCV1, DTP3, ANC1,
84 .
85 . generate abs_mcv1dtp3 = abs(mcv1-dtp3)
   (110 missing values generated)

86 . generate abs_mcv1anc1 = abs(mcv1-anc1)
   (1,089 missing values generated)

87 . generate abs_mcv1pab = abs(mcv1-pab)
   (313 missing values generated)

88 . generate abs_dtp3anc1 = abs(dtp3-anc1)
   (1,110 missing values generated)

89 . generate abs_dtp3pab = abs(dtp3-pab)
   (334 missing values generated)

90 . generate abs_anc1pab = abs(anc1-pab)
   (1,152 missing values generated)

91 .
92 . *Set "integration" to 0 if MCV1, DTP3, ANC1, and PAB all fall below 70%.*
93 .
94 . replace integration=0 if mcv1<70 & dtp3<70 & anc1<70 & pab<70
   (0 real changes made)

95 .
96 . *Set "integration" to 1 if coverage of at least one of the four services is greater than or equ
97 .
98 . replace integration=1 if mcv1>=70 | dtp3>=70 | anc1>=70 | pab>=70
   (12,089 real changes made)

99 .
```

```
100 . *Set "integration" to 2 if co-coverage levels of at least two of the four services are greater
    > ercentage points of one another.*
101 .
102 . replace integration=2 if mcv1>=70 & dtp3>=70 & abs_mcv1dtp3<=10 | mcv1>=70 & anc1>=70 & abs_mcv
    > mcv1pab<=10 | dtp3>=70 & anc1>=70 & abs_dtp3anc1<=10 | dtp3>=70 & pab>=70 & abs_dtp3pab<=10 | a
    (10,517 real changes made)

103 .
104 . *Set "integration" to 3 if co-coverage levels of at least three of the four services are greate
    >  percentage points of one another.*
105 .
106 . replace integration=3 if mcv1>=70 & dtp3>=70 & anc1>=70 & abs_mcv1dtp3<=10 & abs_mcv1anc1<=10 &
    > 70 & pab>=70 & abs_mcv1dtp3<=10 & abs_mcv1pab<=10 & abs_dtp3pab<=10 | mcv1>=70 & anc1>=70 & pab
    > <=10 & abs_anc1pab<=10 | dtp3>=70 & anc1>=70 & pab>=70 & abs_dtp3anc1<=10 & abs_dtp3pab<=10 & a
    (7,627 real changes made)

107 .
108 . *Set "integration" to 4 if co-coverage levels of all four services are greater than or equal to
    >  of one another.*
109 .
110 . replace integration=4 if mcv1>=70 & dtp3>=70 & anc1>=70 & pab>=70 & abs_mcv1dtp3<=10 & abs_mcv1
    > anc1<=10 & abs_dtp3pab<=10 & abs_anc1pab<=10
    (2,503 real changes made)

111 .
112 . *Create a table summarizing all values of "integration."
113 .
114 . tabulate integration
```

Integrated Vaccine Delivery	Freq.	Percent	Cum.
0	**807**	**6.26**	**6.26**
1	**1,572**	**12.19**	**18.45**
2	**2,890**	**22.41**	**40.86**
3	**5,124**	**39.73**	**80.59**
4	**2,503**	**19.41**	**100.00**
Total	**12,896**	**100.00**	

```
115 .
116 . *Create a new variable ("year_dup") that duplicates the "Year" column of the dataset.*
117 .
118 . generate year_dup = year

119 .
120 . ***************************************************************************************************
    > ***
121 . **Group-based trajectory analysis using geographic equity ("geoequity") as the outcome of inter
    > e**
122 . ***************************************************************************************************
    > ***
```

```
123 .
124 . *Specify a directory for reshaped files.*
125 .
126 . cd "C:\Users\sjrav\Desktop\STATA Folder Temp"
    C:\Users\sjrav\Desktop\STATA Folder Temp

127 .
128 . *Reshape the dataset from long to wide format; wide format is required to run the traj Stata pl
129 . mi reshape wide country q5coverage q1coverage geoequity dtp3 mcv1 anc1 pab femeduc polstability
    > exthlth land lingfrac distance ceqr abs_mcv1dtp3 abs_mcv1anc1 abs_mcv1pab abs_dtp3anc1 abs_dtp3
    >  i(alpha3) j(year)

    reshaping m=0 data ...
    (note: j = 2003 2004 2005 2006 2007 2008 2009 2010 2011 2012 2013 2014 2015 2016 2017 2018 2019)

    Data                               long   ->   wide
    -----------------------------------------------------------------------------
    Number of obs.                     1326   ->     78
    Number of variables                  30   ->    477
    j variable (17 values)             year   ->   (dropped)
    xij variables:
                                    country   ->   country2003 country2004 ... country2019
                                 q5coverage   ->   q5coverage2003 q5coverage2004 ... q5coverage2019
                                 q1coverage   ->   q1coverage2003 q1coverage2004 ... q1coverage2019
                                  geoequity   ->   geoequity2003 geoequity2004 ... geoequity2019
                                       dtp3   ->   dtp32003 dtp32004 ... dtp32019
                                       mcv1   ->   mcv12003 mcv12004 ... mcv12019
                                       anc1   ->   anc12003 anc12004 ... anc12019
                                        pab   ->   pab2003 pab2004 ... pab2019
                                    femeduc   ->   femeduc2003 femeduc2004 ... femeduc2019
                               polstability   ->   polstability2003 polstability2004 ... polstability
                                  goveffect   ->   goveffect2003 goveffect2004 ... goveffect2019
                                    corrupt   ->   corrupt2003 corrupt2004 ... corrupt2019
                                        gii   ->   gii2003 gii2004 ... gii2019
                                     oopexp   ->   oopexp2003 oopexp2004 ... oopexp2019
                                     govexp   ->   govexp2003 govexp2004 ... govexp2019
                                    exthlth   ->   exthlth2003 exthlth2004 ... exthlth2019
                                       land   ->   land2003 land2004 ... land2019
                                   lingfrac   ->   lingfrac2003 lingfrac2004 ... lingfrac2019
                                   distance   ->   distance2003 distance2004 ... distance2019
                                       ceqr   ->   ceqr2003 ceqr2004 ... ceqr2019
                               abs_mcv1dtp3   ->   abs_mcv1dtp32003 abs_mcv1dtp32004 ... abs_mcv1dtp3
                               abs_mcv1anc1   ->   abs_mcv1anc12003 abs_mcv1anc12004 ... abs_mcv1anc1
                                abs_mcv1pab   ->   abs_mcv1pab2003 abs_mcv1pab2004 ... abs_mcv1pab201
                               abs_dtp3anc1   ->   abs_dtp3anc12003 abs_dtp3anc12004 ... abs_dtp3anc1
                                abs_dtp3pab   ->   abs_dtp3pab2003 abs_dtp3pab2004 ... abs_dtp3pab201
                                abs_anc1pab   ->   abs_anc1pab2003 abs_anc1pab2004 ... abs_anc1pab201
                                integration   ->   integration2003 integration2004 ... integration201
                                   year_dup   ->   year_dup2003 year_dup2004 ... year_dup2019
    -----------------------------------------------------------------------------

    reshaping m=1 data ...

    reshaping m=2 data ...

    reshaping m=3 data ...

    reshaping m=4 data ...

    reshaping m=5 data ...

    reshaping m=6 data ...

    reshaping m=7 data ...
```

reshaping *m*=8 data ...

reshaping *m*=9 data ...

reshaping *m*=10 data ...

assembling results ...

```
130 .
131 . *Run the traj plugin using a censored normal model, with "year_dup" as the independent variable
    > terest. The censored normal distribution ("cnorm") model is specified because geoequity is a re
    >  censored by minimum and maximum values.*
132 .
133 . *First, drop "geoequity2003" and "year_dup2003" since there is no available outcome data for th
    > imes, changing only the number of groups specified ("order") and holding all other parameters c
    >  produces the highest BIC value.*
134 .
135 . drop geoequity2003

136 . drop year_dup2003

137 . drop integration2003

138 .
139 . traj, model(cnorm) var(geoequity*) indep(year_dup*) min(-1000) max(1000) order(0 0)

    ==== traj stata plugin ====  Jones BL  Nagin DS,  build: Mar 17 2021

    858 observations read.
    858 observations used in the trajectory model.

                            Maximum Likelihood Estimates
                            Model: Censored Normal (cnorm)
```

Group	Parameter	Estimate	Standard Error	T for H0: Parameter=0	Prob > |T|
1	Intercept	37.21814	0.47916	77.674	0.0000
2	Intercept	71.42367	0.37870	188.602	0.0000
	Sigma	29.08228	0.17730	164.029	0.0000
Group membership					
1	(%)	41.32175	1.80656	22.873	0.0000
2	(%)	58.67825	1.80656	32.481	0.0000

```
     BIC=-65502.21 (N=13563)  BIC=-65496.69 (N=858)  AIC=-65487.18  ll= -65483.18

     Entropy = 0.911

140 . traj, model(cnorm) var(geoequity*) indep(year_dup*) min(-1000) max(1000) order(0 0 0)

    ==== traj stata plugin ====  Jones BL  Nagin DS,  build: Mar 17 2021

    858 observations read.
    858 observations used in the trajectory model.

                            Maximum Likelihood Estimates
                            Model: Censored Normal (cnorm)
```

Group	Parameter	Estimate	Standard Error	T for H0: Parameter=0	Prob > |T|
1	Intercept	34.48031	0.63902	53.958	0.0000
2	Intercept	58.93798	1.52642	38.612	0.0000
3	Intercept	76.92272	0.77407	99.375	0.0000
	Sigma	28.47316	0.17618	161.614	0.0000
Group membership					
1	(%)	33.85012	2.17934	15.532	0.0000
2	(%)	29.27772	2.52098	11.614	0.0000
3	(%)	36.87216	3.20559	11.502	0.0000

BIC=-65409.32 (N=13563) BIC=-65401.04 (N=858) AIC=-65386.77 ll= -65380.77

Entropy = 0.761

```
141 . traj, model(cnorm) var(geoequity*) indep(year_dup*) min(-1000) max(1000) order(0 0 0 0)
```

==== traj stata plugin ==== Jones BL Nagin DS, build: Mar 17 2021

858 observations read.
858 observations used in the trajectory model.

Maximum Likelihood Estimates
Model: Censored Normal (cnorm)

Group	Parameter	Estimate	Standard Error	T for H0: Parameter=0	Prob > |T|
1	Intercept	22.18748	1.19543	18.560	0.0000
2	Intercept	41.32798	0.75649	54.631	0.0000
3	Intercept	63.80735	1.12093	56.924	0.0000
4	Intercept	78.75269	0.89054	88.432	0.0000
	Sigma	28.10855	0.17490	160.713	0.0000
Group membership					
1	(%)	9.36152	1.37983	6.785	0.0000
2	(%)	30.67877	1.94765	15.752	0.0000
3	(%)	31.23747	3.12988	9.980	0.0000
4	(%)	28.72223	3.39676	8.456	0.0000

BIC=-65348.06 (N=13563) BIC=-65337.02 (N=858) AIC=-65318.00 ll= -65310.00

Entropy = 0.745

```
142 . traj, model(cnorm) var(geoequity*) indep(year_dup*) min(-1000) max(1000) order(0 0 0 0 0)

    ==== traj stata plugin ====  Jones BL  Nagin DS,  build: Mar 17 2021

    858 observations read.
    858 observations used in the trajectory model.

                            Maximum Likelihood Estimates
                            Model: Censored Normal (cnorm)

                                             Standard       T for H0:
     Group   Parameter        Estimate        Error     Parameter=0   Prob > |T|

     1       Intercept        22.18685      1.19548          18.559       0.0000

     2       Intercept        41.32638      0.75622          54.649       0.0000

     3       Intercept        63.79922      1.11458          57.241       0.0000

     4       Intercept        78.73550      0.82156          95.837       0.0000

     5       Intercept        86.04447     42.68093           2.016       0.0438

             Sigma            28.10837      0.17489         160.718       0.0000

      Group membership
     1       (%)               9.36067      1.37980           6.784       0.0000
     2       (%)              30.67628      1.94742          15.752       0.0000
     3       (%)              31.21126      3.10064          10.066       0.0000
     4       (%)              28.71695      3.40292           8.439       0.0000
     5       (%)               0.03485      0.90134           0.039       0.9692

     BIC=-65357.58 (N=13563)  BIC=-65343.77 (N=858)  AIC=-65320.00  ll= -65310.00

     Entropy = 0.779

143 .
144 . *The two-group model produced a high BIC value, so we will move forward with this option. Next,
    >  group to achieve the largest possible BIC. The model below represents the tested option with t
    > membership in each group. The "detail" command generates parameter estimates that will be used
    > a later step.*
145 .
146 . traj, model(cnorm) var(geoequity*) indep(year_dup*) min(-1000) max(1000) order(1 1) tcov(integr

    ==== traj stata plugin ====  Jones BL  Nagin DS,  build: Mar 17 2021

    858 observations read.
    858 observations used in the trajectory model.

    Start
    Parameter estimates

       35.01100,    0.00000,    0.00000,   79.80680,    0.00000,    0.00000,
       33.59685,   50.00000,   50.00000

                  Neg. Log       Percent
                  Likelihood     Decrease

         0       65968.9581171
    too big of a step
    too big of a step
         1       65751.5765772   0.32952095
         2       65749.3238922   0.00342605
         3       65747.4340507   0.00287431
         4       65743.6878109   0.00569793
```

5	65739.0457890	0.00706079
6	65734.3557857	0.00713427
7	65523.9087774	0.32014767
8	65500.3373013	0.03597386
9	65497.6523533	0.00409914
10	65497.3213103	0.00050543
11	65497.2993145	0.00003358
12	65497.2436275	0.00008502
13	65469.3978156	0.04251448
14	65402.2554153	0.10255540
15	64929.7921616	0.72239596
16	64887.7164876	0.06480180
17	64853.8489856	0.05219401
18	64801.5897323	0.08058003
19	64796.3001512	0.00816273
20	64795.8364988	0.00071555
21	64795.4314414	0.00062513
22	64794.4066987	0.00158150
23	64791.1645075	0.00500381
24	64783.1238211	0.01241016
25	64761.9073839	0.03274994
26	64708.2619383	0.08283488
27	64575.5820429	0.20504321
28	64274.0243521	0.46698408
29	63753.0146241	0.81060698
30	63339.5853713	0.64848581
31	62928.9183533	0.64835760
32	62679.7152629	0.39600727
33	62538.9340260	0.22460414
34	62523.5477515	0.02460271
35	62520.8194404	0.00436365
36	62520.5509345	0.00042947
37	62520.4938731	0.00009127
38	62520.4871447	0.00001076
39	62520.4813751	0.00000923
40	62520.4021108	0.00012678
41	62520.2348776	0.00026749
42	62519.8106931	0.00067848
43	62518.7124596	0.00175662
44	62515.9789242	0.00437235
45	62509.6100490	0.01018760
46	62497.2721119	0.01973766
47	62482.0289358	0.02439015
48	62471.7584717	0.01643747
49	62467.8411770	0.00627051
50	62467.7067824	0.00021514
51	62467.7049393	0.00000295
52	62467.7049121	0.00000004
53	62467.7049101	0.00000000

Maximum Likelihood Estimates
Model: Censored Normal (cnorm)

Group	Parameter	Estimate	Standard Error	T for H0: Parameter=0	Prob > |T|
1	Intercept	-3508.41607	157.90245	-22.219	0.0000
	Linear	1.75532	0.07861	22.329	0.0000
	integration2	6.61464	0.29670	22.294	0.0000
2	Intercept	-8101.39374	118.93935	-68.114	0.0000
	Linear	4.05227	0.05923	68.419	0.0000
	integration2	7.26871	0.33440	21.736	0.0000
	Sigma	23.28173	0.14201	163.943	0.0000

```
  Group membership
 1       (%)             39.68346      1.80566          21.977       0.0000
 2       (%)             60.31654      1.80566          33.404       0.0000

 BIC=-62505.77 (N=13563)  BIC=-62494.72 (N=858)  AIC=-62475.70  ll= -62467.70

Parameter estimates for adding risk factors

-3508.41607,    1.75532,    6.61464,-8101.39374,    4.05227,    7.26871,
   23.28173,    0.41867

Parameter estimates

-3508.41607,    1.75532,    6.61464,-8101.39374,    4.05227,    7.26871,
   23.28173,   39.68346,   60.31654


 Entropy = 0.923

147 .
148 . *Plot the trajectories for the two groups, along with accompanying confidence intervals. Assess
    >  an additional diagnostic check for the model (i.e., narrower, non-overlapping CIs are preferre
149 .
150 . trajplot, xtitle("Year") ytitle("Percentage of districts with 80% MCV1 coverage or higher") ci

151 .
152 . *Perform parametric bootstrap sampling to estimate group size confidence intervals.*
153 .
154 . matrix strt = -3508.41607, 1.75532, 6.61464, -8101.39374, 4.05227, 7.26871, 23.28173, 39.68346,

155 .
156 . bootstrap _b (100/(1+exp(_b[theta2]))) (100*exp(_b[theta2])/(1+exp(_b[theta2]))), reps(1000) do
    > ity*) indep(year_dup*) min(-1000) max(1000) order(1 1) tcov(integration*) start(strt) novar
    (running traj on estimation sample)

Bootstrap replications (1000)
────┼─── 1 ───┼─── 2 ───┼─── 3 ───┼─── 4 ───┼─── 5
..................................................   500
..................................................  1000

Bootstrap results                               Number of obs     =        858
                                                Replications      =      1,000

      command:  traj, model(cnorm) var(geoequity*) indep(year_dup*) min(-1000) max(1000) order(1
                    novar
  [_eq2]_bs_1:  100/(1+exp(_b[theta2]))
  [_eq2]_bs_2:  100*exp(_b[theta2])/(1+exp(_b[theta2]))
```

	Observed Coef.	Bootstrap Std. Err.	z	P>|z|	Normal-based [95% Conf.	Interval]
**_eq1**						
interc1	**-3508.416**	**230.3577**	**-15.23**	**0.000**	**-3959.909**	**-3056.923**
linear1	**1.755322**	**.1148566**	**15.28**	**0.000**	**1.530207**	**1.980437**
integration2G1	**6.61464**	**.5016852**	**13.18**	**0.000**	**5.631355**	**7.597925**
interc2	**-8101.394**	**164.3837**	**-49.28**	**0.000**	**-8423.58**	**-7779.208**
linear2	**4.052272**	**.0821005**	**49.36**	**0.000**	**3.891359**	**4.213186**
integration2G2	**7.26871**	**.4921719**	**14.77**	**0.000**	**6.304071**	**8.233349**
sigma	**23.28172**	**.2549613**	**91.31**	**0.000**	**22.782**	**23.78143**
theta2	**.4186719**	**.0845333**	**4.95**	**0.000**	**.2529897**	**.5843541**
**_eq2**						
_bs_1	**39.68346**	**2.017103**	**19.67**	**0.000**	**35.73001**	**43.63691**
_bs_2	**60.31654**	**2.017103**	**29.90**	**0.000**	**56.36309**	**64.26999**

```
157 .
158 . estat bootstrap, percentile bc

Bootstrap results                               Number of obs     =        858
                                                Replications      =       1000

      command:  traj, model(cnorm) var(geoequity*) indep(year_dup*) min(-1000) max(1000) order(1
                    novar
  [_eq2]_bs_1:  100/(1+exp(_b[theta2]))
  [_eq2]_bs_2:  100*exp(_b[theta2])/(1+exp(_b[theta2]))
```

	Observed Coef.	Bias	Bootstrap Std. Err.	[95% Conf.	Interval]	
**_eq1**						
interc1	**-3508.4161**	**9.88824**	**230.35772**	**-3948.979**	**-3017.529**	(P)
				**-3950.927**	**-3022.375**	(BC)
linear1	**1.755322**	**-.0049411**	**.11485661**	**1.510217**	**1.974707**	(P)
				**1.513038**	**1.975496**	(BC)
integratio~1	**6.61464**	**.0158754**	**.50168517**	**5.663135**	**7.671028**	(P)
				**5.64512**	**7.632634**	(BC)
interc2	**-8101.3937**	**3.586975**	**164.38365**	**-8408.338**	**-7776.332**	(P)
				**-8411.163**	**-7779.813**	(BC)
linear2	**4.0522725**	**-.0018116**	**.08210047**	**3.889759**	**4.205448**	(P)
				**3.892282**	**4.207128**	(BC)
integratio~2	**7.2687099**	**.0188222**	**.49217193**	**6.342952**	**8.263218**	(P)
				**6.273336**	**8.211984**	(BC)
sigma	**23.281717**	**-.0164842**	**.25496134**	**22.76464**	**23.79466**	(P)
				**22.82456**	**23.84458**	(BC)
theta2	**.41867188**	**.0010867**	**.08453328**	**.2564751**	**.5947749**	(P)
				**.2540792**	**.5897055**	(BC)
**_eq2**						
_bs_1	**39.68346**	**-.0082364**	**2.0171027**	**35.55401**	**43.62304**	(P)
				**35.67024**	**43.68197**	(BC)
_bs_2	**60.31654**	**.0082364**	**2.0171027**	**56.37696**	**64.446**	(P)
				**56.31803**	**64.32976**	(BC)

```
(P)    percentile confidence interval
(BC)   bias-corrected confidence interval

159 .
160 . *Create a program, "trajstats," to calculate several other diagnostic criteria for group-based
161 .
162 . program trajstats
  1.
163 . preserve
  2.
164 . *This step calculates the average posterior probability.*
165 .
```

```
166 . generate Mp=0
      3.
167 . foreach i of varlist _traj_ProbG* {
      4.               replace Mp = `i' if `i' > Mp
      5.          }
      6.     sort _traj_Group
      7.
168 . by _traj_Group: generate countG = _N
      8.
169 . *This step calculates the odds of correct classification.*
170 .
171 .     by _traj_Group: egen groupAPP = mean(Mp)
      9.     by _traj_Group: generate counter = _n
     10.     generate n = groupAPP/(1 - groupAPP)
     11.     generate p = countG/ _N
     12.     generate d = p/(1-p)
     13.     generate occ = n/d
     14.
172 . *This step calculates the estimated group probabilities vs. the proportion of the sample assign
173 .
174 .         scalar c = 0
     15.     gen TotProb = 0
     16.     foreach i of varlist _traj_ProbG* {
     17.        scalar c = c + 1
     18.        quietly summarize `i'
     19.        replace TotProb = r(sum)/ _N if _traj_Group == c
     20.     }
     21.         gen d_pp = TotProb/(1 - TotProb)
     22.         gen occ_pp = n/d_pp
     23.
175 .  *This step displays:
176 .     *Group number [_traj_~p],
177 .     *Count per group (based on the max post prob), [countG]
178 .     *Average posterior probability for each group, [groupAPP]
179 .     *Odds of correct classification (based on the maximum posterior group assignment rule), [oc
180 .     *Odds of correct classification (based on the weighted posterior probabilities), [occ_pp]
181 .     *Observed probability of groups [p] versus the probability based on the posterior probabili
182 .
183 . list _traj_Group countG groupAPP occ occ_pp p TotProb if counter == 1
     24.
184 . restore
     25.
185 . end

186 .
187 . *Now, run the trajstats program.*
188 .
189 . trajstats
    (858 real changes made)
    (511 real changes made)
    (347 real changes made)
    (511 real changes made)
```

	traj~p	countG	groupAPP	occ	occ_pp	p	TotProb
1.	1	347	.9624406	37.73522	38.94767	.4044289	.3968351
348.	2	511	.9872454	52.56156	50.9253	.5955711	.603165

```
190 .
    end of do-file

191 . graph export "C:\Users\sjrav\Desktop\Dissertation\Aim 3 - Quantitative Analysis\Data\STATA Outp
    > - trajplot.png", as(png) replace
    (file C:\Users\sjrav\Desktop\Dissertation\Aim 3 - Quantitative Analysis\Data\STATA Output\MI GBTM
    > .png written in PNG format)

192 . do "C:\Users\sjrav\AppData\Local\Temp\STD1e2c_000000.tmp"

193 . *Reshape the dataset from wide to long format.*
194 .
195 . mi reshape long country q5coverage q1coverage geoequity dtp3 mcv1 anc1 pab femeduc polstability
    > exthlth land lingfrac distance ceqr abs_mcv1dtp3 abs_mcv1anc1 abs_mcv1pab abs_dtp3anc1 abs_dtp3
    >  i(alpha3) j(year)
    (imputed variable geoequity2003 unregistered because not in m=0)

    reshaping m=0 data ...
    (note: j = 2003 2004 2005 2006 2007 2008 2009 2010 2011 2012 2013 2014 2015 2016 2017 2018 2019)
    (note: geoequity2003 not found)
    (note: integration2003 not found)
    (note: year_dup2003 not found)

    Data                                   wide   ->   long
    -----------------------------------------------------------------------------
    Number of obs.                           78   ->    1326
    Number of variables                     477   ->      33
    j variable (17 values)                        ->   year
    xij variables:
    country2003 country2004 ... country2019   ->   country
    q5coverage2003 q5coverage2004 ... q5coverage2019->q5coverage
    q1coverage2003 q1coverage2004 ... q1coverage2019->q1coverage
    geoequity2003 geoequity2004 ... geoequity2019->geoequity
             dtp32003 dtp32004 ... dtp32019   ->   dtp3
             mcv12003 mcv12004 ... mcv12019   ->   mcv1
             anc12003 anc12004 ... anc12019   ->   anc1
                pab2003 pab2004 ... pab2019   ->   pab
    femeduc2003 femeduc2004 ... femeduc2019   ->   femeduc
    polstability2003 polstability2004 ... polstability2019->polstability
    goveffect2003 goveffect2004 ... goveffect2019->goveffect
    corrupt2003 corrupt2004 ... corrupt2019   ->   corrupt
                gii2003 gii2004 ... gii2019   ->   gii
       oopexp2003 oopexp2004 ... oopexp2019   ->   oopexp
       govexp2003 govexp2004 ... govexp2019   ->   govexp
    exthlth2003 exthlth2004 ... exthlth2019   ->   exthlth
             land2003 land2004 ... land2019   ->   land
    lingfrac2003 lingfrac2004 ... lingfrac2019->   lingfrac
    distance2003 distance2004 ... distance2019->   distance
             ceqr2003 ceqr2004 ... ceqr2019   ->   ceqr
    abs_mcv1dtp32003 abs_mcv1dtp32004 ... abs_mcv1dtp32019->abs_mcv1dtp3
    abs_mcv1anc12003 abs_mcv1anc12004 ... abs_mcv1anc12019->abs_mcv1anc1
    abs_mcv1pab2003 abs_mcv1pab2004 ... abs_mcv1pab2019->abs_mcv1pab
    abs_dtp3anc12003 abs_dtp3anc12004 ... abs_dtp3anc12019->abs_dtp3anc1
    abs_dtp3pab2003 abs_dtp3pab2004 ... abs_dtp3pab2019->abs_dtp3pab
    abs_anc1pab2003 abs_anc1pab2004 ... abs_anc1pab2019->abs_anc1pab
    integration2003 integration2004 ... integration2019->integration
    year_dup2003 year_dup2004 ... year_dup2019->   year_dup
    -----------------------------------------------------------------------------

    reshaping m=1 data ...

    reshaping m=2 data ...

    reshaping m=3 data ...
```

reshaping *m*=4 data ...

reshaping *m*=5 data ...

reshaping *m*=6 data ...

reshaping *m*=7 data ...

reshaping *m*=8 data ...

reshaping *m*=9 data ...

reshaping *m*=10 data ...

assembling results ...

```
196 .
197 . *Perform multinomial logistic regression to identify covariates with statistically significant
    > etting Group 1 membership as the base outcome.*
198 .
199 . mlogit _traj_Group femeduc polstability goveffect corrupt gii oopexp govexp exthlth land lingfr
    > 1)

    Iteration 0:   log likelihood = -2112.8518
    Iteration 1:   log likelihood = -1649.5314
    Iteration 2:   log likelihood = -1624.3003
    Iteration 3:   log likelihood = -1621.6018
    Iteration 4:   log likelihood = -1621.4697
    Iteration 5:   log likelihood = -1621.4697

    Multinomial logistic regression                 Number of obs     =      3,286
                                                    LR chi2(12)       =     982.76
                                                    Prob > chi2       =     0.0000
    Log likelihood = -1621.4697                     Pseudo R2         =     0.2326
```

_traj_Group	Coef.	Std. Err.	z	P>|z|	[95% Conf.	Interval]
**1**	(base outcome)					
**2**						
femeduc	-.0131726	.0031188	-4.22	0.000	-.0192852	-.0070599
polstability	-.1589456	.0804469	-1.98	0.048	-.3166187	-.0012725
goveffect	2.346586	.1676277	14.00	0.000	2.018042	2.675131
corrupt	-.9473753	.1416815	-6.69	0.000	-1.225066	-.6696847
gii	2.357569	.2474551	9.53	0.000	1.872566	2.842572
oopexp	.0153801	.003042	5.06	0.000	.0094179	.0213422
govexp	.003264	.0005131	6.36	0.000	.0022584	.0042697
exthlth	.0392419	.0035584	11.03	0.000	.0322675	.0462163
land	5.02e-07	9.25e-08	5.43	0.000	3.20e-07	6.83e-07
lingfrac	-2.795083	.2120787	-13.18	0.000	-3.21075	-2.379417
distance	-.0300936	.0023104	-13.03	0.000	-.0346219	-.0255653
integration	.0886595	.0498386	1.78	0.075	-.0090224	.1863414
_cons	2.446878	.3885081	6.30	0.000	1.685416	3.20834

```
200 .
    end of do-file

201 .
```
