# Supplementary material for: Group-based trajectory models of integrated vaccine delivery and equity in low- and middle-income countries
Source: Int J Equity Health. 2024 Jan 9;23:5. doi: 10.1186/s12939-023-02088-x (PMC10775446; doi:10.1186/s12939-023-02088-x)
Supplement: Supplementary file 5 — Additional file 5. [file 12939_2023_2088_MOESM5_ESM.pdf]

```
  ___  ____  ____  ____  ____ (R)
 /__    /   ____/   /   ____/
___/   /   /___/   /   /___/   15.1
  Statistics/Data Analysis
```

Copyright 1985-2017 StataCorp LLC
StataCorp
4905 Lakeway Drive
College Station, Texas 77845 USA
800-STATA-PC   http://www.stata.com
979-696-4600   stata@stata.com
979-696-4601 (fax)

Notes:
1. Unicode is supported; see help unicode advice.

```
. doedit "C:\

. do "C:\

. *Import dataset.*
.
. import delimited "C:\
(21 vars, 1,326 obs)

.
. *Assign labels to each variable in the dataset.*
.
. label variable alpha3 "Alpha-3 Code"

. label variable q5coverage "MCV1 Coverage in the Wealthiest Quintile"

. label variable q1coverage "MCV1 Coverage in the Poorest Quintile"

. label variable geoequity "Geographic Equity"

. label variable dtp3 "DTP3 Coverage"

. label variable mcv1 "MCV1 Coverage"

. label variable anc1 "Antenatal Care Coverage (1+ Visits)"

. label variable pab "Protection at Birth Against Neonatal Tetanus"

. label variable femeduc "Female Primary Education Completed"

. label variable polstability "Political Stability"

. label variable goveffect "Government Effectiveness"
```

```
. label variable corrupt "Corruption"

. label variable gii "Gender Inequality Index"

. label variable oopexp "Out-of-Pocket Health Expenditures"

. label variable govexp "Domestic Government Health Expenditures"

. label variable exthlth "External Health Spending"

. label variable land "Land Area"

. label variable lingfrac "Linguistic Fractionalization"

. label variable distance "Distance to Nearest Health Facility (Walking, 60+ Minutes)"

.
. ****************************************************************************************
. ***Creating new variables for socioeconomic vaccine equity & integrated vaccine delivery.***
. ****************************************************************************************
.
. *Create and label a new variable, "ceqr," representing the ratio of MCV1 coverage in the wealth
.
. generate ceqr = q5coverage/q1coverage
(1,113 missing values generated)

. label variable ceqr "Socioeconomic Equity"

.
. *Create and label a new variable, integration, representing integrated vaccine delivery.*
.
. generate integration = 0

. label variable integration "Integrated Vaccine Delivery"

.
. *Create new variables representing all possible absolute differences between MCV1, DTP3, ANC1,
.
. generate abs_mcv1dtp3 = abs(mcv1-dtp3)
(10 missing values generated)

. generate abs_mcv1anc1 = abs(mcv1-anc1)
(1,009 missing values generated)

. generate abs_mcv1pab = abs(mcv1-pab)
(233 missing values generated)

. generate abs_dtp3anc1 = abs(dtp3-anc1)
(1,010 missing values generated)

. generate abs_dtp3pab = abs(dtp3-pab)
(234 missing values generated)
```

```
50 . generate abs_anc1pab = abs(anc1-pab)
   (1,072 missing values generated)

51 .
52 . *Set "integration" to 0 if MCV1, DTP3, ANC1, and PAB all fall below 70%.*
53 .
54 . replace integration=0 if mcv1<70 & dtp3<70 & anc1<70 & pab<70
   (0 real changes made)

55 .
56 . *Set "integration" to 1 if coverage of at least one of the four services is greater than or equ
57 .
58 . replace integration=1 if mcv1>=70 | dtp3>=70 | anc1>=70 | pab>=70
   (1,304 real changes made)

59 .
60 . *Set "integration" to 2 if co-coverage levels of at least two of the four services are greater 
   > ercentage points of one another.*
61 .
62 . replace integration=2 if mcv1>=70 & dtp3>=70 & abs_mcv1dtp3<=10 | mcv1>=70 & anc1>=70 & abs_mcv
   > mcv1pab<=10 | dtp3>=70 & anc1>=70 & abs_dtp3anc1<=10 | dtp3>=70 & pab>=70 & abs_dtp3pab<=10 | a
   (1,006 real changes made)

63 .
64 . *Set "integration" to 3 if co-coverage levels of at least three of the four services are greate
   >  percentage points of one another.*
65 .
66 . replace integration=3 if mcv1>=70 & dtp3>=70 & anc1>=70 & abs_mcv1dtp3<=10 & abs_mcv1anc1<=10 &
   > 70 & pab>=70 & abs_mcv1dtp3<=10 & abs_mcv1pab<=10 & abs_dtp3pab<=10 | mcv1>=70 & anc1>=70 & pab
   > <=10 & abs_anc1pab<=10 | dtp3>=70 & anc1>=70 & pab>=70 & abs_dtp3anc1<=10 & abs_dtp3pab<=10 & a
   (585 real changes made)

67 .
68 . *Set "integration" to 4 if co-coverage levels of all four services are greater than or equal to
   >  of one another.*
69 .
70 . replace integration=4 if mcv1>=70 & dtp3>=70 & anc1>=70 & pab>=70 & abs_mcv1dtp3<=10 & abs_mcv1
   > anc1<=10 & abs_dtp3pab<=10 & abs_anc1pab<=10
   (63 real changes made)

71 .
72 . *Create a table summarizing all values of "integration."
73 .
74 . tabulate integration
```

Integrated Vaccine Delivery	Freq.	Percent	Cum.
0	**22**	**1.66**	**1.66**
1	**298**	**22.47**	**24.13**
2	**421**	**31.75**	**55.88**
3	**522**	**39.37**	**95.25**
4	**63**	**4.75**	**100.00**
Total	**1,326**	**100.00**	

```
75 .
76 . *Create a new variable ("year_dup") that duplicates the "Year" column of the dataset.*
77 .
78 . generate year_dup = year

79 .
   end of do-file

80 . do "C:\Users\sjrav\AppData\Local\Temp\STD102c_000000.tmp"

81 . ************************************************************************************************
   > ***
82 . ***Group-based trajectory analysis using socioeconomic equity ("ceqr") as the outcome of intere
   > ***
83 . ************************************************************************************************
   > ***
84 .
85 . *Repeat the trajectory analysis using "ceqr" (socioeconomic vaccine equity) as the outcome of i
   > g variable. Re-import the dataset, create the variable "integration," etc.*
86 .
87 . *Reshape the dataset from long to wide format; wide format is required to run the traj Stata pl
88 . reshape wide country q5coverage q1coverage geoequity dtp3 mcv1 anc1 pab femeduc polstability go
   > hlth land lingfrac distance ceqr abs_mcv1dtp3 abs_mcv1anc1 abs_mcv1pab abs_dtp3anc1 abs_dtp3pab
   > alpha3) j(year)
   (note: j = 2003 2004 2005 2006 2007 2008 2009 2010 2011 2012 2013 2014 2015 2016 2017 2018 2019)

   Data                               long   ->   wide
   -----------------------------------------------------------------------------
   Number of obs.                     1326   ->      78
   Number of variables                  30   ->     477
   j variable (17 values)             year   ->   (dropped)
   xij variables:
                                   country   ->   country2003 country2004 ... country2019
                                q5coverage   ->   q5coverage2003 q5coverage2004 ... q5coverage2019
                                q1coverage   ->   q1coverage2003 q1coverage2004 ... q1coverage2019
                                 geoequity   ->   geoequity2003 geoequity2004 ... geoequity2019
                                      dtp3   ->   dtp32003 dtp32004 ... dtp32019
                                      mcv1   ->   mcv12003 mcv12004 ... mcv12019
                                      anc1   ->   anc12003 anc12004 ... anc12019
                                       pab   ->   pab2003 pab2004 ... pab2019
                                   femeduc   ->   femeduc2003 femeduc2004 ... femeduc2019
                              polstability   ->   polstability2003 polstability2004 ... polstability
                                 goveffect   ->   goveffect2003 goveffect2004 ... goveffect2019
                                   corrupt   ->   corrupt2003 corrupt2004 ... corrupt2019
                                       gii   ->   gii2003 gii2004 ... gii2019
                                    oopexp   ->   oopexp2003 oopexp2004 ... oopexp2019
                                    govexp   ->   govexp2003 govexp2004 ... govexp2019
                                   exthlth   ->   exthlth2003 exthlth2004 ... exthlth2019
                                      land   ->   land2003 land2004 ... land2019
                                  lingfrac   ->   lingfrac2003 lingfrac2004 ... lingfrac2019
                                  distance   ->   distance2003 distance2004 ... distance2019
                                      ceqr   ->   ceqr2003 ceqr2004 ... ceqr2019
                              abs_mcv1dtp3   ->   abs_mcv1dtp32003 abs_mcv1dtp32004 ... abs_mcv1dtp3
                              abs_mcv1anc1   ->   abs_mcv1anc12003 abs_mcv1anc12004 ... abs_mcv1anc1
                               abs_mcv1pab   ->   abs_mcv1pab2003 abs_mcv1pab2004 ... abs_mcv1pab201
                              abs_dtp3anc1   ->   abs_dtp3anc12003 abs_dtp3anc12004 ... abs_dtp3anc1
                               abs_dtp3pab   ->   abs_dtp3pab2003 abs_dtp3pab2004 ... abs_dtp3pab201
                               abs_anc1pab   ->   abs_anc1pab2003 abs_anc1pab2004 ... abs_anc1pab201
                               integration   ->   integration2003 integration2004 ... integration201
                                  year_dup   ->   year_dup2003 year_dup2004 ... year_dup2019
   -----------------------------------------------------------------------------
```

```
89 .
90 . *Run the plugin several times, changing only the number of groups specified ("order") and holdi
   > ect the group number that produces the highest BIC value.*
91 .
92 . traj, model(cnorm) var(ceqr*) indep(year_dup*) min(0) max(100) order (0 0)

   ==== traj stata plugin ====  Jones BL  Nagin DS,  build: Mar 17 2021

   78 observations read.
   9 had no trajectory data.
   69 observations used in the trajectory model.

                            Maximum Likelihood Estimates
                            Model: Censored Normal (cnorm)

                                             Standard       T for H0:
    Group   Parameter        Estimate         Error     Parameter=0   Prob > |T|

    1       Intercept         1.30917      0.03235          40.474       0.0000

    2       Intercept         3.83211      0.15708          24.396       0.0000

            Sigma             0.45353      0.02224          20.396       0.0000

     Group membership
    1       (%)              96.70227      2.32377          41.614       0.0000
    2       (%)               3.29773      2.32377           1.419       0.1573

    BIC=  -153.72 (N=213)  BIC=  -151.46 (N=69)  AIC=  -146.99  ll=   -142.99

    Entropy = 0.956

93 . traj, model(cnorm) var(ceqr*) indep(year_dup*) min(0) max(100) order (0 0 0)

   ==== traj stata plugin ====  Jones BL  Nagin DS,  build: Mar 17 2021

   78 observations read.
   9 had no trajectory data.
   69 observations used in the trajectory model.

                            Maximum Likelihood Estimates
                            Model: Censored Normal (cnorm)

                                             Standard       T for H0:
    Group   Parameter        Estimate         Error     Parameter=0   Prob > |T|

    1       Intercept         2.12900      0.11432          18.623       0.0000

    2       Intercept         1.23249      0.03045          40.476       0.0000

    3       Intercept         4.30515      0.14293          30.122       0.0000

            Sigma             0.37365      0.01903          19.630       0.0000

     Group membership
    1       (%)              13.59572      4.78556           2.841       0.0049
    2       (%)              84.95492      4.94332          17.186       0.0000
    3       (%)               1.44937      1.45602           0.995       0.3207

    BIC=  -136.95 (N=213)  BIC=  -133.57 (N=69)  AIC=  -126.87  ll=   -120.87

    Entropy = 0.838
```

```
94 . traj, model(cnorm) var(ceqr*) indep(year_dup*) min(0) max(100) order (0 0 0 0)

   ==== traj stata plugin ====  Jones BL  Nagin DS,  build: Mar 17 2021

   78 observations read.
   9 had no trajectory data.
   69 observations used in the trajectory model.

                        Maximum Likelihood Estimates
                        Model: Censored Normal (cnorm)

                                          Standard       T for H0:
    Group   Parameter        Estimate        Error     Parameter=0   Prob > |T|

    1       Intercept         1.18527      0.03510          33.765       0.0000

    2       Intercept         1.77021      0.08357          21.181       0.0000

    3       Intercept         2.76229      0.16775          16.467       0.0000

    4       Intercept         4.30517      0.13225          32.554       0.0000

            Sigma             0.34409      0.01893          18.176       0.0000

     Group membership
    1       (%)              74.96326      7.26990          10.311       0.0000
    2       (%)              19.35628      6.93336           2.792       0.0057
    3       (%)               4.23117      2.64867           1.597       0.1116
    4       (%)               1.44929      1.46299           0.991       0.3230

    BIC=  -135.06 (N=213)  BIC=  -130.55 (N=69)  AIC=  -121.61  ll=   -113.61

    Entropy = 0.697

95 . traj, model(cnorm) var(ceqr*) indep(year_dup*) min(0) max(100) order (0 0 0 0 0)

   ==== traj stata plugin ====  Jones BL  Nagin DS,  build: Mar 17 2021

   78 observations read.
   9 had no trajectory data.
   69 observations used in the trajectory model.

                        Maximum Likelihood Estimates
                        Model: Censored Normal (cnorm)

                                          Standard       T for H0:
    Group   Parameter        Estimate        Error     Parameter=0   Prob > |T|

    1       Intercept         1.18527      0.14576           8.131       0.0000

    2       Intercept         1.18527      0.04202          28.210       0.0000

    3       Intercept         1.77021      0.08398          21.078       0.0000

    4       Intercept         2.76229      0.16857          16.387       0.0000

    5       Intercept         4.30517      0.13289          32.396       0.0000

            Sigma             0.34409      0.01902          18.087       0.0000

     Group membership
    1       (%)              10.41682  44390.04670           0.000       0.9998
    2       (%)              64.54645  44390.04624           0.001       0.9988
    3       (%)              19.35627      7.03278           2.752       0.0064
    4       (%)               4.23118      2.65508           1.594       0.1125
```

```
 5       (%)               1.44929      1.47329           0.984       0.3264

 BIC=  -140.42 (N=213)  BIC=  -134.78 (N=69)  AIC=  -123.61  ll=   -113.61
```

Warning: variance matrix is nonsymmetric or highly singular

```
 Entropy = 0.581

96 .
97 . *The three-group model produced the largest BIC value, so we will move forward with this optior
   > or each of the four groups to achieve the largest possible BIC. The model below represents the
   > high entropy, and at least 1% membership in the smallest group.*
98 .
99 . traj, model (cnorm) var(ceqr*) indep(year_dup*) min(0) max(100) order (0 1 0) tcov(integration*

==== traj stata plugin ====  Jones BL  Nagin DS,  build: Mar 17 2021

78 observations read.
9 had no trajectory data.
69 observations used in the trajectory model.

Start
Parameter estimates

    0.72526,    0.00000,    1.43083,    0.00000,    0.00000,    2.13641,
    0.00000,    0.70558,   33.33333,   33.33333,   33.33333

              Neg. Log       Percent
              Likelihood     Decrease

     0         226.9413254
     1         224.3433006   1.14480022
     2         224.3177128   0.01140565
     3         207.7389386   7.39075572
     4         194.2147038   6.51020695
     5         186.1502213   4.15235425
     6         178.6386724   4.03520817
     7         167.9231464   5.99843571
     8         155.1998833   7.57683706
     9         148.3032601   4.44370380
    10         136.1171482   8.21702229
    11         125.2100619   8.01301408
    12         118.1043889   5.67500163
    13         111.9428180   5.21705496
    14         105.7993263   5.48806240
    15         105.7637251   0.03364974
    16         105.7408996   0.02158158
    17         105.3504941   0.36920962
    18         104.2769626   1.01900945
    19         104.2096633   0.06453900
    20         102.0303433   2.09128397
    21         100.9681275   1.04107834
    22         100.1857419   0.77488375
    23          99.7536249   0.43131584
    24          99.7025861   0.05116483
    25          99.6902503   0.01237259
    26          99.6530081   0.03735796
    27          99.6364195   0.01664633
    28          99.6312015   0.00523707
    29          99.6232328   0.00799820
    30          99.6054135   0.01788664
    31          99.5487392   0.05689881
    32          99.5281619   0.02067058
    33          99.2352360   0.29431462
    34          98.9393498   0.29816644
    35          98.4293229   0.51549449
```

36	97.7006305	0.74032045
37	97.1191651	0.59515010
38	95.7850648	1.37367358
39	93.8645019	2.00507553
40	92.2022175	1.77094049
41	91.6593050	0.58882796
42	91.5509678	0.11819557
43	91.5284256	0.02462260
44	91.5175272	0.01190712
45	91.5071818	0.01130420
46	91.4683165	0.04247247
47	91.4117697	0.06182114
48	91.3320103	0.08725294
49	91.2829003	0.05377075
50	91.2698152	0.01433468
51	91.2688774	0.00102757
52	91.2688563	0.00002307
53	91.2688561	0.00000024
54	91.2688561	0.00000000

Maximum Likelihood Estimates
Model: Censored Normal (cnorm)

Group	Parameter	Estimate	Standard Error	T for H0: Parameter=0	Prob > |T|
1	Intercept	2.42788	0.24762	9.805	0.0000
	integration2	-0.22938	0.10530	-2.178	0.0305
2	Intercept	-2.69469	11.49512	-0.234	0.8149
	Linear	0.00219	0.00573	0.382	0.7029
	integration2	-0.16401	0.02965	-5.532	0.0000
3	Intercept	4.15015	0.11149	37.223	0.0000
	integration2	-2.87112	0.35274	-8.140	0.0000
	Sigma	0.32734	0.01736	18.851	0.0000
Group membership					
1	(%)	12.70901	5.64893	2.250	0.0255
2	(%)	84.39238	5.92067	14.254	0.0000
3	(%)	2.89861	2.06379	1.405	0.1616

BIC= -118.08 (N=213) BIC= -112.44 (N=69) AIC= -101.27 ll= -91.27

Parameter estimates for adding risk factors

2.42788, -0.22938, -2.69469, 0.00219, -0.16401, 4.15015,
-2.87112, 0.32734, 1.89317, -1.47808

Parameter estimates

2.42788, -0.22938, -2.69469, 0.00219, -0.16401, 4.15015,
-2.87112, 0.32734, 12.70901, 84.39238, 2.89861

Entropy = 0.703

```
100 .
101 . *Plot the trajectories for the three groups, along with accompanying confidence intervals. Asse
    > es an additional diagnostic check for the model (i.e., narrower, non-overlapping CIs are prefer
102 .
103 . trajplot, xtitle("Year") ytitle("Socioeconomic Vaccine Equity Ratio") ci

104 .
105 . *Perform parametric bootstrap sampling to estimate group size confidence intervals.*
106 .
107 . matrix strt = 2.42788, -0.22938, -2.69469, 0.00219, -0.16401, 4.15015, -2.87112, 0.32734, 12.70

108 .
109 . bootstrap _b (100/(1+exp(_b[theta2]))) (100*exp(_b[theta2])/(1+exp(_b[theta2]))), reps(1000) do
    >  indep(year_dup*) min(0) max(110) order(0 1 0) tcov(integration*) start(strt) novar
    (running traj on estimation sample)

    Bootstrap replications (1000)
    ────┼─── 1 ───┼─── 2 ───┼─── 3 ───┼─── 4 ───┼─── 5
    ..................................................   500
    ..................................................  1000

    Bootstrap results                                Number of obs     =         78
                                                     Replications      =        993

          command:  traj, model(cnorm) var(ceqr*) indep(year_dup*) min(0) max(110) order(0 1 0) tcov(
      [_eq2]_bs_1:  100/(1+exp(_b[theta2]))
      [_eq2]_bs_2:  100*exp(_b[theta2])/(1+exp(_b[theta2]))
```

	Observed Coef.	Bootstrap Std. Err.	z	P>|z|	Normal-based [95% Conf.	Interval]
**_eq1**						
interc1	**2.427881**	**.5221046**	**4.65**	**0.000**	**1.404575**	**3.451188**
integration2G1	**-.2293803**	**.2276194**	**-1.01**	**0.314**	**-.6755061**	**.2167456**
interc2	**-2.69469**	**13.86172**	**-0.19**	**0.846**	**-29.86316**	**24.47378**
linear2	**.0021874**	**.0069789**	**0.31**	**0.754**	**-.011491**	**.0158658**
integration2G2	**-.1640136**	**.060712**	**-2.70**	**0.007**	**-.2830071**	**-.0450202**
interc3	**4.150147**	**.5404707**	**7.68**	**0.000**	**3.090844**	**5.20945**
integration2G3	**-2.871122**	**.8074618**	**-3.56**	**0.000**	**-4.453718**	**-1.288525**
sigma	**.3273346**	**.0572921**	**5.71**	**0.000**	**.2150443**	**.439625**
theta2	**1.893165**	**2.35164**	**0.81**	**0.421**	**-2.715964**	**6.502295**
theta3	**-1.47808**	**7.020148**	**-0.21**	**0.833**	**-15.23732**	**12.28116**
**_eq2**						
_bs_1	**13.0884**	**10.81997**	**1.21**	**0.226**	**-8.118348**	**34.29515**
_bs_2	**86.9116**	**10.81997**	**8.03**	**0.000**	**65.70485**	**108.1183**

```
    Note: One or more parameters could not be estimated in 7 bootstrap replicates;
          standard-error estimates include only complete replications.

110 .
111 . estat bootstrap, percentile bc

    Bootstrap results                                Number of obs     =         78
                                                     Replications      =        993

          command:  traj, model(cnorm) var(ceqr*) indep(year_dup*) min(0) max(110) order(0 1 0) tcov(
      [_eq2]_bs_1:  100/(1+exp(_b[theta2]))
      [_eq2]_bs_2:  100*exp(_b[theta2])/(1+exp(_b[theta2]))
```

	Observed Coef.	Bias	Bootstrap Std. Err.	[95% Conf.	Interval]	
**_eq1**						
interc1	**2.4278812**	**-.039195**	**.52210463**	**1.346565**	**3.526153**	(P)
				**1.677544**	**3.946244**	(BC)
integratio~1	**-.22938027**	**.0071198**	**.22761941**	**-.8196257**	**.3061728**	(P)
				**-.8791572**	**.0776538**	(BC)
interc2	**-2.69469**	**3.011022**	**13.861717**	**-24.99538**	**25.81442**	(P)
				**-31.79388**	**21.38537**	(BC)
linear2	**.00218743**	**-.001532**	**.00697889**	**-.0122041**	**.0135169**	(P)
				**-.0100766**	**.0167612**	(BC)
integratio~2	**-.16401364**	**.0210064**	**.06071204**	**-.2310562**	**-.0006093**	(P)
				**-.2524408**	**-.0355059**	(BC)
interc3	**4.150147**	**-.1039263**	**.54047069**	**3.607574**	**4.305168**	(P)
				**3.60757**	**4.305168**	(BC)
integrati~G3	**-2.8711215**	**.3840458**	**.80746182**	**-2.960094**	**-.8588111**	(P)
				**-17.99372**	**-2.328633**	(BC)
sigma	**.32733464**	**-.0134357**	**.05729206**	**.2134433**	**.43461**	(P)
				**.2368034**	**.4737191**	(BC)
theta2	**1.8931652**	**.1607706**	**2.3516399**	**.3209563**	**3.80779**	(P)
				**.6755874**	**17.51871**	(BC)
theta3	**-1.4780795**	**-1.9637**	**7.0201476**	**-22.85132**	**1.177824**	(P)
				**-22.77366**	**1.187684**	(BC)
**_eq2**						
_bs_1	**13.0884**	**3.351745**	**10.819968**	**2.171516**	**42.04427**	(P)
				**2.46e-06**	**33.72469**	(BC)
_bs_2	**86.9116**	**-3.351745**	**10.819968**	**57.95573**	**97.82848**	(P)
				**66.27531**	**100**	(BC)

```
(P)    percentile confidence interval
(BC)   bias-corrected confidence interval
Note: One or more parameters could not be estimated in 7 bootstrap replicates;
      standard-error estimates include only complete replications.

. 
. *Create a program, "trajstats," to calculate several other diagnostic criteria for group-based
. 
. program trajstats
  1. 
. preserve
  2. 
. *This step calculates the average posterior probability.*
. 
. generate Mp=0
  3. 
. foreach i of varlist _traj_ProbG* {
  4.              replace Mp = `i' if `i' > Mp
  5.          }
  6.     sort _traj_Group
  7. 
```

```
121 . by _traj_Group: generate countG = _N
  8.
122 . *This step calculates the odds of correct classification.*
123 .
124 .     by _traj_Group: egen groupAPP = mean(Mp)
  9.     by _traj_Group: generate counter = _n
 10.     generate n = groupAPP/(1 - groupAPP)
 11.     generate p = countG/ _N
 12.     generate d = p/(1-p)
 13.     generate occ = n/d
 14.
125 . *This step calculates the estimated group probabilities vs. the proportion of the sample assig
126 .
127 .         scalar c = 0
 15.     gen TotProb = 0
 16.     foreach i of varlist _traj_ProbG* {
 17.        scalar c = c + 1
 18.        quietly summarize `i'
 19.        replace TotProb = r(sum)/ _N if _traj_Group == c
 20.     }
 21.         gen d_pp = TotProb/(1 - TotProb)
 22.         gen occ_pp = n/d_pp
 23.
128 .  *This step displays:
129 .     *Group number [_traj_~p],
130 .     *Count per group (based on the max post prob), [countG]
131 .     *Average posterior probability for each group, [groupAPP]
132 .     *Odds of correct classification (based on the maximum posterior group assignment rule), [oc
133 .     *Odds of correct classification (based on the weighted posterior probabilities), [occ_pp]
134 .     *Observed probability of groups [p] versus the probability based on the posterior probabili
135 .
136 . list _traj_Group countG groupAPP occ occ_pp p TotProb if counter == 1
 24.
137 . restore
 25.
138 . end

139 .
140 . *Run the trajstats program to perform model diagnostics.*
141 .
142 . trajstats
    (77 real changes made)
    (67 real changes made)
    (2 real changes made)
    (9 real changes made)
    (67 real changes made)
    (2 real changes made)
```

	traj~p	countG	groupAPP	occ	occ_pp	p	TotProb
1.	1	9	.8243302	35.97582	32.22996	.1153846	.1270906
10.	2	67	.958881	3.828603	4.312788	.8589743	.8439231
77.	3	2	.9999985	2.45e+07	2.16e+07	.025641	.0289863

```
.
. *Create a list of group assignments and group membership probabilities for all subjects.*
.
. list _traj_Group - _traj_ProbG3
```

	traj~p	_traj_~1	_traj_~2	_traj_~3
1.	2	.0000941	.9999059	1.50e-15
2.	1	.9744619	.0255381	1.37e-37
3.	2	.0004645	.9995355	0
4.	2	.0000512	.9999487	0
5.	2	.0543283	.9456717	1.65e-21
6.	2	.0006904	.9993096	0
7.	2	.085706	.914294	0
8.	2	.0226249	.9773751	0
9.	2	6.89e-06	.9999931	0
10.	2	.0088617	.9911383	0
11.	2	.000541	.999459	0
12.	2	.1270902	.8439237	.0289861
13.	2	.0598217	.9401783	6.40e-20
14.	2	.1270902	.8439237	.0289861
15.	1	.5802357	.4197643	0
16.	1	.7048917	.2951083	0
17.	2	.013982	.986018	0
18.	2	.1654904	.8345096	0
19.	2	.1291536	.8708464	0
20.	2	.1270902	.8439237	.0289861
21.	2	.1270902	.8439237	.0289861
22.	2	.1270902	.8439237	.0289861
23.	2	.0137899	.9862102	6.83e-28
24.	2	.1270902	.8439237	.0289861
25.	2	.0010112	.9989888	0
26.	1	.9872755	.0127245	0
27.	2	.0000412	.9999588	0
28.	2	.0004463	.9995537	0
29.	2	.0156139	.9843861	0
30.	2	.0133901	.9866099	0
31.	2	.0000564	.9999436	5.80e-35
32.	2	.0028184	.9971815	0
33.	1	.6090932	.3909068	0
34.	2	.0264486	.9735515	0
35.	2	.0000938	.9999062	0
36.	2	.0119327	.9880673	0
37.	2	.0422212	.9577788	0
38.	2	.023619	.9763811	0
39.	2	.0125882	.9874118	0
40.	2	.1270902	.8439237	.0289861
41.	2	.0003043	.9996957	0
42.	2	.0077428	.9922572	0
43.	1	.9292977	.0707023	0
44.	2	.0000776	.9999223	0
45.	2	.0130976	.9869024	0
46.	2	.0000261	.9999739	0
47.	2	.0535328	.9464672	0
48.	2	.0286653	.9713347	0

```
 49.           2    .000048    .999952          0
 50.           2   .0066991   .9933009          0

 51.           3          0          0          1
 52.           2   .1270902   .8439237   .0289861
 53.           2   .0000101   .9999899          0
 54.           1   .9963554   .0036447          0
 55.           1   .6760216   .3239784   4.13e-30

 56.           2   .0458308   .9541692          0
 57.           2   .0000826   .9999174          0
 58.           2   .0756492   .9243508          0
 59.           2   .0000232   .9999768          0
 60.           2   .1270902   .8439237   .0289861

 61.           2   6.84e-07   .9999993          0
 62.           2   .2006732   .7993268   4.03e-10
 63.           1   .9613391   .0386021   .0000589
 64.           2    .001035    .998965          0
 65.           2   .0224104   .9775897          0

 66.           3   3.02e-06   4.32e-13    .999997
 67.           2   .0084377   .9915623          0
 68.           2   .0000642   .9999357          0
 69.           2   .0013049   .9986951          0
 70.           2    .108612   .8913881          0

 71.           2   .0181121   .9818879          0
 72.           2   .0000392   .9999608          0
 73.           2   .0050644   .9949356   3.11e-16
 74.           2    .014767   .9852329          0
 75.           2   .0313622   .9686378          0

 76.           2   2.71e-06   .9999973          0
 77.           2   .0007021   .9992979          0
 78.           2   .0000181   .9999819          0


147 .
    end of do-file

148 . graph export "C:\Users\sjrav\Desktop\Dissertation\Aim 3 - Quantitative Analysis\Data\STATA Outp
    > - trajplot.png", as(png) replace
    could not find Graph window
    r(693);

149 . do "C:\Users\sjrav\AppData\Local\Temp\STD102c_000000.tmp"

150 . reshape long
    (note: j = 2003 2004 2005 2006 2007 2008 2009 2010 2011 2012 2013 2014 2015 2016 2017 2018 2019)

    Data                                   wide   ->   long
    -----------------------------------------------------------------------------
    Number of obs.                           78   ->    1326
    Number of variables                     481   ->      34
    j variable (17 values)                        ->   year
    xij variables:
    country2003 country2004 ... country2019   ->   country
    q5coverage2003 q5coverage2004 ... q5coverage2019->q5coverage
    q1coverage2003 q1coverage2004 ... q1coverage2019->q1coverage
    geoequity2003 geoequity2004 ... geoequity2019->geoequity
             dtp32003 dtp32004 ... dtp32019   ->   dtp3
             mcv12003 mcv12004 ... mcv12019   ->   mcv1
             anc12003 anc12004 ... anc12019   ->   anc1
                pab2003 pab2004 ... pab2019   ->   pab
```

```
femeduc2003 femeduc2004 ... femeduc2019   ->   femeduc
polstability2003 polstability2004 ... polstability2019->polstability
goveffect2003 goveffect2004 ... goveffect2019->goveffect
corrupt2003 corrupt2004 ... corrupt2019   ->   corrupt
            gii2003 gii2004 ... gii2019   ->   gii
   oopexp2003 oopexp2004 ... oopexp2019   ->   oopexp
   govexp2003 govexp2004 ... govexp2019   ->   govexp
exthlth2003 exthlth2004 ... exthlth2019   ->   exthlth
         land2003 land2004 ... land2019   ->   land
lingfrac2003 lingfrac2004 ... lingfrac2019->   lingfrac
distance2003 distance2004 ... distance2019->   distance
         ceqr2003 ceqr2004 ... ceqr2019   ->   ceqr
abs_mcv1dtp32003 abs_mcv1dtp32004 ... abs_mcv1dtp32019->abs_mcv1dtp3
abs_mcv1anc12003 abs_mcv1anc12004 ... abs_mcv1anc12019->abs_mcv1anc1
abs_mcv1pab2003 abs_mcv1pab2004 ... abs_mcv1pab2019->abs_mcv1pab
abs_dtp3anc12003 abs_dtp3anc12004 ... abs_dtp3anc12019->abs_dtp3anc1
abs_dtp3pab2003 abs_dtp3pab2004 ... abs_dtp3pab2019->abs_dtp3pab
abs_anc1pab2003 abs_anc1pab2004 ... abs_anc1pab2019->abs_anc1pab
integration2003 integration2004 ... integration2019->integration
year_dup2003 year_dup2004 ... year_dup2019->   year_dup
```

```
151 .
152 . *Perform multinomial logistic regression to identify covariates with statistically significant
    > etting Group 1 membership as the base outcome.*
153 .
154 . mlogit _traj_Group femeduc polstability goveffect corrupt gii oopexp govexp exthlth land lingfr
    > 1)

Iteration 0:   log likelihood = -179.04955
Iteration 1:   log likelihood = -160.32314
Iteration 2:   log likelihood = -129.23603
Iteration 3:   log likelihood = -110.34248
Iteration 4:   log likelihood = -95.788705
Iteration 5:   log likelihood = -90.755274
Iteration 6:   log likelihood = -89.352092
Iteration 7:   log likelihood = -88.885284
Iteration 8:   log likelihood = -88.789346
Iteration 9:   log likelihood = -88.766746
Iteration 10:  log likelihood = -88.761253
Iteration 11:  log likelihood = -88.760128
Iteration 12:  log likelihood =  -88.75992
Iteration 13:  log likelihood = -88.759899
Iteration 14:  log likelihood = -88.759894

Multinomial logistic regression                 Number of obs     =        346
                                                LR chi2(24)       =     180.58
                                                Prob > chi2       =     0.0000
Log likelihood = -88.759894                     Pseudo R2         =     0.5043
```

_traj_Group	Coef.	Std. Err.	z	P>|z|	[95% Conf.	Interval]
1	(base outcome)					
2						
femeduc	.0244232	.0152222	1.60	0.109	-.0054117	.0542581
polstability	.0423123	.333347	0.13	0.899	-.6110357	.6956603
goveffect	-1.828646	.8749003	-2.09	0.037	-3.543419	-.1138726
corrupt	.5421276	.8823969	0.61	0.539	-1.187339	2.271594
gii	-1.764196	1.095428	-1.61	0.107	-3.911195	.3828027
oopexp	-.0102206	.0141131	-0.72	0.469	-.0378818	.0174405
govexp	.019156	.0061616	3.11	0.002	.0070796	.0312324
exthlth	.0589978	.0244449	2.41	0.016	.0110866	.1069089
land	-6.60e-07	3.46e-07	-1.91	0.056	-1.34e-06	1.69e-08

lingfrac	**2.001122**	**.9702362**	**2.06**	**0.039**	**.0994942**	**3.90275**
distance	**.0140563**	**.01335**	**1.05**	**0.292**	**-.0121093**	**.040222**
integration	**.7673035**	**.2582852**	**2.97**	**0.003**	**.2610737**	**1.273533**
_cons	**-4.958165**	**1.713945**	**-2.89**	**0.004**	**-8.317436**	**-1.598894**
**3**						
femeduc	**-.8416916**	**327.2962**	**-0.00**	**0.998**	**-642.3305**	**640.6471**
polstability	**21.78257**	**7202.625**	**0.00**	**0.998**	**-14095.1**	**14138.67**
goveffect	**-6.558478**	**18000.4**	**-0.00**	**1.000**	**-35286.69**	**35273.58**
corrupt	**-49.70104**	**22753.52**	**-0.00**	**0.998**	**-44645.77**	**44546.37**
gii	**-63.39735**	**14768.16**	**-0.00**	**0.997**	**-29008.46**	**28881.66**
oopexp	**1.760913**	**262.9148**	**0.01**	**0.995**	**-513.5426**	**517.0644**
govexp	**.0745374**	**16.70213**	**0.00**	**0.996**	**-32.66104**	**32.81012**
exthlth	**.398066**	**530.8453**	**0.00**	**0.999**	**-1040.04**	**1040.836**
land	**.0000502**	**.0086052**	**0.01**	**0.995**	**-.0168158**	**.0169161**
lingfrac	**-100.0659**	**32307.89**	**-0.00**	**0.998**	**-63422.37**	**63222.24**
distance	**-1.558608**	**253.3983**	**-0.01**	**0.995**	**-498.2101**	**495.0929**
integration	**-31.34055**	**6971.836**	**-0.00**	**0.996**	**-13695.89**	**13633.21**
_cons	**45.05146**	**36315.43**	**0.00**	**0.999**	**-71131.89**	**71221.99**

Note: 21 observations completely determined. Standard errors questionable.

155 .
end of do-file

156 .
