# Supplementary material for: Group-based trajectory models of integrated vaccine delivery and equity in low- and middle-income countries
Source: Int J Equity Health. 2024 Jan 9;23:5. doi: 10.1186/s12939-023-02088-x (PMC10775446; doi:10.1186/s12939-023-02088-x)
Supplement: Supplementary file 6 — Additional file 6. [file 12939_2023_2088_MOESM6_ESM.pdf]

```
  ___  ____  ____  ____  ____ (R)
 /__    /   ____/   /   ____/
___/   /   /___/   /   /___/   15.1   Copyright 1985-2017 StataCorp LLC
  Statistics/Data Analysis            StataCorp
                                      4905 Lakeway Drive
                                      College Station, Texas 77845 USA
                                      800-STATA-PC        http://www.stata.com
                                      979-696-4600        stata@stata.com
                                      979-696-4601 (fax)


Notes:
      1.  Unicode is supported; see help unicode advice.

. doedit "C:\

. do "C:

. *Import dataset.*
.
. import delimited "C:\
(21 vars, 1,326 obs)

.
. *Assign labels to each variable in the dataset.*
.
. label variable alpha3 "Alpha-3 Code"

. label variable q5coverage "MCV1 Coverage in the Wealthiest Quintile"

. label variable q1coverage "MCV1 Coverage in the Poorest Quintile"

. label variable geoequity "Geographic Equity"

. label variable dtp3 "DTP3 Coverage"

. label variable mcv1 "MCV1 Coverage"

. label variable anc1 "Antenatal Care Coverage (1+ Visits)"

. label variable pab "Protection at Birth Against Neonatal Tetanus"

. label variable femeduc "Female Primary Education Completed"

. label variable polstability "Political Stability"

. label variable goveffect "Government Effectiveness"
```

```
20 . label variable corrupt "Corruption"

21 . label variable gii "Gender Inequality Index"

22 . label variable oopexp "Out-of-Pocket Health Expenditures"

23 . label variable govexp "Domestic Government Health Expenditures"

24 . label variable exthlth "External Health Spending"

25 . label variable land "Land Area"

26 . label variable lingfrac "Linguistic Fractionalization"

27 . label variable distance "Distance to Nearest Health Facility (Walking, 60+ Minutes)"

28 .
29 . ******************************************************
30 . ***Perform multiple imputation to handle missing data***
31 . ******************************************************
32 .
33 . *First, identify missing values in the dataset. Install the "mdesc" package if needed.*
34 .
35 . mdesc q5coverage q1coverage geoequity dtp3 mcv1 anc1 pab femeduc polstability goveffect corrupt
   > rac distance
```

Variable	Missing	Total	Percent Missing
q5coverage	1,110	1,326	83.71
q1coverage	1,113	1,326	83.94
geoequity	173	1,326	13.05
dtp3	10	1,326	0.75
mcv1	8	1,326	0.60
anc1	1,008	1,326	76.02
pab	233	1,326	17.57
femeduc	537	1,326	40.50
polstability	8	1,326	0.60
goveffect	8	1,326	0.60
corrupt	7	1,326	0.53
gii	649	1,326	48.94
oopexp	140	1,326	10.56
govexp	140	1,326	10.56
exthlth	140	1,326	10.56
land	8	1,326	0.60
lingfrac	51	1,326	3.85
distance	24	1,326	1.81

```
36 .
37 . *Create a pairwise correlation matrix to identify potential auxiliary variables.*
38 .
39 . pwcorr q5coverage q1coverage geoequity dtp3 mcv1 anc1 pab femeduc polstability goveffect corrup
   > frac distance
```

	q5cove~e	q1cove~e	geoequ~y	dtp3	mcv1	anc1	pab
q5coverage	**1.0000**						
q1coverage	**0.6281**	**1.0000**					
geoequity	**0.2300**	**0.3073**	**1.0000**				
dtp3	**0.5752**	**0.8465**	**0.4849**	**1.0000**			
mcv1	**0.5457**	**0.8753**	**0.5076**	**0.9327**	**1.0000**		
anc1	**0.4729**	**0.5951**	**0.3168**	**0.6588**	**0.6774**	**1.0000**	
pab	**0.4227**	**0.5877**	**0.4671**	**0.5965**	**0.5821**	**0.4524**	**1.0000**
femeduc	**0.3703**	**0.5205**	**0.3353**	**0.5543**	**0.6116**	**0.4605**	**0.3549**
polstability	**0.2494**	**0.4526**	**0.2423**	**0.5301**	**0.4956**	**0.5417**	**0.3489**
goveffect	**0.3012**	**0.3078**	**0.2922**	**0.4600**	**0.4626**	**0.3677**	**0.3302**
corrupt	**0.2340**	**0.2832**	**0.1959**	**0.4221**	**0.4001**	**0.3809**	**0.3100**
gii	**0.0286**	**0.1052**	**0.0784**	**0.1231**	**0.1048**	**0.0473**	**-0.0129**
oopexp	**-0.2164**	**-0.3076**	**-0.0370**	**-0.1919**	**-0.1982**	**-0.3969**	**-0.0332**
govexp	**0.0955**	**0.3204**	**0.1907**	**0.2190**	**0.2678**	**0.2461**	**0.1847**
exthlth	**0.1314**	**0.2588**	**0.0542**	**0.1583**	**0.1176**	**0.1924**	**0.1691**
land	**-0.0590**	**-0.3470**	**0.0120**	**-0.0627**	**-0.0418**	**0.0529**	**-0.2108**
lingfrac	**-0.0580**	**-0.2804**	**-0.1435**	**-0.2875**	**-0.3367**	**-0.1515**	**-0.0855**
distance	**-0.0729**	**0.0351**	**0.0133**	**0.0245**	**0.0984**	**-0.1866**	**-0.0842**

	femeduc	polsta~y	goveff~t	corrupt	gii	oopexp	govexp
femeduc	**1.0000**						
polstability	**0.3344**	**1.0000**					
goveffect	**0.4249**	**0.4793**	**1.0000**				
corrupt	**0.2481**	**0.5881**	**0.7399**	**1.0000**			
gii	**-0.0221**	**-0.0051**	**0.0268**	**0.0044**	**1.0000**		
oopexp	**-0.0728**	**-0.4911**	**-0.2437**	**-0.4723**	**-0.0045**	**1.0000**	
govexp	**0.2737**	**0.2610**	**0.2712**	**0.3047**	**-0.1186**	**-0.2392**	**1.0000**
exthlth	**0.0355**	**0.2336**	**-0.0415**	**0.1797**	**-0.0721**	**-0.4768**	**-0.1415**
land	**-0.1103**	**-0.1418**	**0.1638**	**-0.0273**	**0.1948**	**0.0910**	**0.0105**
lingfrac	**-0.4576**	**-0.1729**	**-0.0583**	**-0.0730**	**0.0277**	**0.0683**	**-0.3200**
distance	**0.2705**	**0.0602**	**-0.0898**	**-0.0958**	**-0.0326**	**0.0677**	**0.2000**

	exthlth	land	lingfrac	distance
exthlth	**1.0000**			
land	**-0.2399**	**1.0000**		
lingfrac	**0.0890**	**0.0268**	**1.0000**	
distance	**-0.2347**	**0.0031**	**-0.3394**	**1.0000**

```
40 .
41 . *Set the data for multiple imputation. The "mi set mlong" command generates three additional va
   > the imputed datasets and values*
42 .
43 . mi set mlong

44 .
45 . *Summarize missing values in the dataset.*
46 .
47 . mi misstable summarize q5coverage q1coverage geoequity dtp3 mcv1 anc1 pab femeduc polstability 
   > xthlth land lingfrac distance
```

				Obs<.		
Variable	Obs=.	Obs>.	Obs<.	Unique values	Min	Max
q5coverage	**1,110**		**216**	153	20	100
q1coverage	**1,113**		**213**	182	8.2	100
geoequity	**173**		**1,153**	102	0	104
dtp3	**10**		**1,316**	75	19	99
mcv1	**8**		**1,318**	72	16	99
anc1	**1,008**		**318**	204	16.1	100
pab	**233**		**1,093**	63	31	99

```
       femeduc |        537              789 |     >500   16.65596    125.6158
  polstability |          8            1,318 |      357      -3.31        1.42
     goveffect |          8            1,318 |      251      -2.48         .83
       corrupt |          7            1,319 |      235      -1.87        1.65
           gii |        649              677 |      419       .038        .776
        oopexp |        140            1,186 |       85          0          86
        govexp |        140            1,186 |      286          1        2873
       exthlth |        140            1,186 |      102          0         189
          land |          8            1,318 |      175        810     9424703
      lingfrac |         51            1,275 |       74          0       .9227
      distance |         24            1,302 |       76        6.8        91.9
-----------------------------------------------------------------------------

48 .
49 . *Summarize missing patterns in the dataset.*
50 .
51 . mi misstable patterns q5coverage q1coverage geoequity dtp3 mcv1 anc1 pab femeduc polstability g
> thlth land lingfrac distance

                     Missing-value patterns
                       (1 means complete)

              |   Pattern
      Percent |  1  2  3  4    5  6  7  8    9 10 11 12   13 14 15 16
  ------------+------------------------------------------------------
           4% |  1  1  1  1    1  1  1  1    1  1  1  1    1  1  1  1
              |  1  1
              |
           15 |  1  1  1  1    1  1  1  1    1  1  1  1    1  1  0  0
              |  0  0
              |
           15 |  1  1  1  1    1  1  1  1    1  1  1  1    1  1  1  0
              |  0  0
              |
           10 |  1  1  1  1    1  1  1  1    1  1  1  1    1  0  1  0
              |  0  0
              |
            7 |  1  1  1  1    1  1  1  1    1  1  1  1    1  0  0  0
              |  0  0
              |
            3 |  1  1  1  1    1  1  1  1    1  1  1  1    1  1  0  1
              |  1  1
              |
            3 |  1  1  1  1    1  1  1  1    0  0  0  1    1  0  1  0
              |  0  0
              |
            3 |  1  1  1  1    1  1  1  1    1  1  1  1    0  1  1  0
              |  0  0
              |
            3 |  1  1  1  1    1  1  1  1    1  1  1  0    1  1  0  0
              |  0  0
              |
            3 |  1  1  1  1    1  1  1  1    1  1  1  1    0  1  0  0
              |  0  0
              |
            3 |  1  1  1  1    1  1  1  1    1  1  1  1    1  0  1  1
              |  1  1
              |
            2 |  1  1  1  1    1  1  1  1    1  1  1  1    0  0  0  0
              |  0  0
              |
            2 |  1  1  1  1    1  1  1  1    1  1  1  0    0  1  0  0
              |  0  0
              |
            2 |  1  1  1  1    1  1  1  1    1  1  1  1    1  1  1  1
```

																	0	0
**2**	1	1	1	1	1	1	1	1	1	1	1	0	1	0	0	0	0	0
**1**	1	1	1	1	1	1	1	1	0	0	0	1	1	0	0	0	0	0
**1**	1	1	1	1	1	1	1	1	1	1	1	1	1	0	0	1	1	1
**1**	1	1	1	1	1	1	1	1	0	0	0	1	1	1	1	0	0	0
**1**	1	1	1	1	1	1	1	1	1	1	1	1	0	0	1	0	0	0
<1	1	1	1	1	1	1	1	1	1	1	1	1	0	1	0	1	0	0
<1	1	1	1	1	1	1	1	1	1	1	1	1	1	0	0	1	0	0
<1	1	1	1	1	1	1	1	1	1	1	1	1	1	1	0	1	0	0
<1	1	1	1	1	1	1	1	1	1	1	1	1	1	0	1	1	0	0
<1	1	1	1	1	1	1	1	0	1	1	1	1	1	0	1	0	0	0
<1	1	1	1	1	1	1	1	1	1	1	1	0	0	0	0	0	0	0
<1	1	1	1	1	1	1	1	1	1	1	1	1	0	0	1	1	0	0
<1	1	1	1	1	1	1	1	1	1	1	1	1	0	1	1	1	0	0
<1	1	1	1	1	1	1	1	1	1	1	1	1	0	1	1	1	1	1
<1	1	1	1	1	1	1	1	1	0	0	0	0	1	0	0	0	0	0
<1	1	1	1	1	1	1	1	1	1	1	1	0	1	1	1	0	0	0
<1	0	0	0	0	0	0	0	0	0	0	0	0	0	0	0	0	0	0
<1	1	1	1	1	1	1	0	1	1	1	1	1	1	1	1	0	0	0
<1	1	1	1	1	1	1	1	1	1	1	1	0	1	1	0	1	0	0
<1	1	1	1	1	1	1	1	0	1	1	1	1	1	0	0	0	0	0
<1	1	1	1	1	1	1	1	1	0	0	0	1	1	0	1	1	1	1

<1	1	1	1	1	1	1	1	1	1	1	1	0	0	1	0	1	
	0	0															
<1	1	1	1	1	1	1	1	1	1	1	1	0	1	0	1	0	
	0	0															
<1	1	1	1	1	1	1	1	0	0	0	0	1	1	0	1	0	
	0	0															
<1	1	1	1	1	1	1	1	0	1	1	1	1	1	1	1	0	
	0	0															
<1	1	1	1	1	1	1	1	1	0	0	0	1	0	1	1	0	
	0	0															
<1	1	1	1	1	1	1	1	1	0	0	0	1	1	1	0	0	
	0	0															
<1	1	1	1	1	1	1	1	1	1	1	1	1	1	1	1	0	
	1	1															
<1	1	1	1	1	1	1	0	1	1	1	1	0	1	0	0	0	
	0	0															
<1	1	1	1	1	1	1	1	1	1	1	1	0	0	1	1	0	
	0	0															
<1	1	1	1	1	1	1	1	1	1	1	1	0	1	0	0	1	
	1	1															
<1	1	1	1	1	1	1	1	1	1	1	1	0	1	1	0	1	
	1	1															
<1	1	1	1	1	1	1	1	1	1	1	1	1	0	1	0	1	
	1	1															
<1	1	1	1	1	1	1	1	0	1	1	1	1	1	0	1	1	
	1	1															
<1	1	1	1	1	1	1	1	1	0	0	0	1	1	0	0	1	
	1	1															
<1	1	1	1	1	1	1	1	1	0	0	0	1	1	0	1	1	
	0	0															
<1	1	1	1	1	1	1	1	1	0	0	0	1	1	1	1	1	
	1	1															
<1	1	1	1	1	1	1	1	1	1	1	1	0	1	0	0	1	
	0	0															
<1	1	1	1	1	1	1	1	1	1	1	1	1	0	0	0	1	
	0	0															
<1	1	1	1	1	1	1	1	1	1	1	1	1	1	1	0	0	
	1	1															
<1	1	1	1	1	1	1	0	1	1	1	1	1	1	0	0	0	
	0	0															
<1	1	1	1	1	1	1	1	0	0	0	0	1	1	1	1	0	
	0	0															
<1	1	1	1	1	1	1	1	0	1	1	1	0	1	1	0	0	
	0	0															

<1	1	1	1	1	1	1	1	1	0	0	0	1	0	0	1	0	0	0
<1	1	1	1	1	1	1	1	1	1	1	1	0	0	0	0	1	0	0
<1	1	1	1	1	1	1	1	1	1	1	1	0	1	0	1	0	1	0
<1	1	1	1	1	1	1	1	1	1	1	1	0	1	1	1	1	1	1
<1	1	1	1	1	1	1	1	1	1	1	1	1	0	0	0	1	1	1
<1	1	1	1	1	1	1	1	1	1	1	1	1	0	0	1	1	1	1
<1	1	0	0	0	0	0	1	0	0	0	0	0	0	0	1	1	1	1
<1	1	1	1	1	1	0	1	1	1	1	1	0	0	1	0	1	0	0
<1	1	1	1	1	1	0	1	1	1	1	1	1	1	0	0	0	1	1
<1	1	1	1	1	1	1	0	1	0	0	0	1	1	0	1	0	0	0
<1	1	1	1	1	1	1	0	1	1	1	1	0	1	0	0	1	0	0
<1	1	1	1	1	1	1	0	1	1	1	1	1	1	0	1	0	0	0
<1	1	1	1	1	1	1	0	1	1	1	1	1	1	1	1	1	0	0
<1	1	1	1	1	1	1	1	0	0	0	0	0	1	0	1	0	0	0
<1	1	1	1	1	1	1	1	0	0	0	0	1	1	0	1	1	0	0
<1	1	1	1	1	1	1	1	0	1	1	1	0	1	0	0	0	0	0
<1	1	1	1	1	1	1	1	0	1	1	1	0	1	0	1	0	0	0
<1	1	1	1	1	1	1	1	0	1	1	1	0	1	1	1	1	1	1
<1	1	1	1	1	1	1	1	0	1	1	1	1	1	0	0	1	1	1
<1	1	1	1	1	1	1	1	0	1	1	1	1	1	0	1	1	0	0
<1	1	1	1	1	1	1	1	0	1	1	1	1	1	1	0	0	0	0
<1	1	1	1	1	1	1	1	0	1	1	1	1	1	1	0	1		

	1	1														
<1	1	1	1	1	1	1	1	0	1	1	1	1	1	1	1	1
	0	0														
<1	1	1	1	1	1	1	1	0	1	1	1	1	1	1	1	1
	1	1														
<1	1	1	1	1	1	1	1	1	0	0	0	0	0	0	0	0
	0	0														
<1	1	1	1	1	1	1	1	1	0	0	0	0	0	0	1	0
	0	0														
<1	1	1	1	1	1	1	1	1	0	0	0	0	1	0	1	0
	0	0														
<1	1	1	1	1	1	1	1	1	0	0	0	0	1	1	0	0
	0	0														
<1	1	1	1	1	1	1	1	1	0	0	0	1	0	0	0	0
	0	0														
<1	1	1	1	1	1	1	1	1	0	0	0	1	0	0	0	1
	0	0														
<1	1	1	1	1	1	1	1	1	0	0	0	1	0	1	0	0
	0	0														
<1	1	1	1	1	1	1	1	1	0	0	0	1	0	1	1	1
	0	0														
<1	1	1	1	1	1	1	1	1	0	0	0	1	1	0	0	0
	1	1														
<1	1	1	1	1	1	1	1	1	0	0	0	1	1	0	0	1
	0	0														
<1	1	1	1	1	1	1	1	1	0	0	0	1	1	1	0	1
	0	0														
<1	1	1	1	1	1	1	1	1	1	1	1	0	0	0	0	1
	1	1														
<1	1	1	1	1	1	1	1	1	1	1	1	0	0	0	1	0
	0	0														
<1	1	1	1	1	1	1	1	1	1	1	1	0	0	0	1	1
	1	1														
<1	1	1	1	1	1	1	1	1	1	1	1	0	0	1	0	1
	1	1														
<1	1	1	1	1	1	1	1	1	1	1	1	0	1	0	0	0
	1	1														
<1	1	1	1	1	1	1	1	1	1	1	1	0	1	0	1	1
	0	0														
<1	1	1	1	1	1	1	1	1	1	1	1	0	1	1	1	0
	1	0														
<1	1	1	1	1	1	1	1	1	1	1	1	1	1	0	1	0
	1	1														

```
      100%
```

```
  Variables are
      Row 1:   (1) corrupt  (2) goveffect  (3) land  (4) mcv1  (5) polstability  (6) dtp3  (7) di
               (10) govexp  (11) oopexp  (12) geoequity  (13) pab  (14) femeduc  (15) gii  (16) a
      Row 2:   (1) q5coverage  (2) q1coverage

52 .
53 . *Register the variables to be imputed.*
54 .
55 . mi register imputed q5coverage q1coverage geoequity anc1 pab
   (1157 m=0 obs. now marked as incomplete)

56 .
57 . *Specify the imputed model.*
58 .
59 . mi impute mvn geoequity anc1 pab = mcv1, add(10) rseed(54321) force

   Performing EM optimization:
   note: 37 observations omitted from EM estimation because of all imputation variables missing
     observed log likelihood = -8367.4122 at iteration 40

   Performing MCMC data augmentation ...

   Multivariate imputation                      Imputations =       10
   Multivariate normal regression                     added =       10
   Imputed: m=1 through m=10                        updated =        0

   Prior: uniform                                Iterations =     1000
                                                    burn-in =      100
                                                    between =      100
```

	Observations per *m*			
Variable	Complete	Incomplete	Imputed	Total
geoequity	**1153**	**173**	**165**	**1326**
anc1	**318**	**1008**	**1001**	**1326**
pab	**1093**	**233**	**225**	**1326**

```
   (complete + incomplete = total; imputed is the minimum across m
    of the number of filled-in observations.)

   Note: Right-hand-side variables (or weights) have missing values;
         model parameters estimated using listwise deletion.

60 .
61 . *Specify a linear regression model to estimate the missing values.*
62 .
63 . mi estimate: regress q5coverage q1coverage geoequity anc1 pab

   Multiple-imputation estimates                Imputations       =         10
   Linear regression                            Number of obs     =        212
                                                Average RVI       =     0.1211
                                                Largest FMI       =     0.2395
                                                Complete DF       =        207
   DF adjustment:   Small sample                DF:     min       =      82.41
                                                        avg       =     135.34
                                                        max       =     186.54
   Model F test:       Equal FMI                F(   4,  181.6)   =      29.27
   Within VCE type:          OLS                Prob > F          =     0.0000
```

q5coverage	Coef.	Std. Err.	t	P>|t|	[95% Conf.	Interval]
q1coverage	.2735273	.0421289	6.49	0.000	.190417	.3566377
geoequity	.0000196	.0226001	0.00	0.999	-.044601	.0446402
anc1	.0722887	.0531523	1.36	0.178	-.0334402	.1780176
pab	.1054068	.0831516	1.27	0.208	-.0598366	.2706501
_cons	51.2888	5.458233	9.40	0.000	40.50594	62.07167

```
.
. *Perform a diagnostic check to assess how well the imputation was performed.*
.
. mi estimate, vartable dftable
```

Multiple-imputation estimates   Imputations   =   10
Linear regression

Variance information

	Imputation variance					Relative
	Within	Between	Total	RVI	FMI	efficiency
q1coverage	.001692	.000076	.001775	.049141	.047829	.99524
geoequity	.00047	.000037	.000511	.087544	.082642	.991804
anc1	.002179	.000587	.002825	.296505	.239515	.976609
pab	.005428	.001351	.006914	.27388	.224846	.97801
_cons	26.8366	2.68696	29.7923	.110135	.102158	.989887

```
Multiple-imputation estimates                    Imputations      =         10
Linear regression                                Number of obs    =        212
                                                 Average RVI      =     0.1211
                                                 Largest FMI      =     0.2395
                                                 Complete DF      =        207
DF adjustment:   Small sample                    DF:     min      =      82.41
                                                         avg      =     135.34
                                                         max      =     186.54
Model F test:       Equal FMI                    F(   4,  181.6)  =      29.27
Within VCE type:          OLS                    Prob > F         =     0.0000
```

q5coverage	Coef.	Std. Err.	t	P>|t|	DF	% Increase Std. Err.
q1coverage	.2735273	.0421289	6.49	0.000	186.5	2.43
geoequity	.0000196	.0226001	0.00	0.999	166.0	4.29
anc1	.0722887	.0531523	1.36	0.178	82.4	13.86
pab	.1054068	.0831516	1.27	0.208	88.1	12.87
_cons	51.2888	5.458233	9.40	0.000	153.7	5.36

```
68 .
69 . ***************************************************************************************
70 . ***Creating new variables for socioeconomic vaccine equity & integrated vaccine delivery.***
71 . ***************************************************************************************
72 .
73 . *Create and label a new variable, "ceqr," representing the ratio of MCV1 coverage in the wealth
74 .
75 . generate ceqr = q5coverage/q1coverage
   (12,243 missing values generated)

76 . label variable ceqr "Socioeconomic Equity"

77 .
78 . *Create and label a new variable, integration, representing integrated vaccine delivery.*
79 .
80 . generate integration = 0

81 . label variable integration "Integrated Vaccine Delivery"

82 .
83 . *Create new variables representing all possible absolute differences between MCV1, DTP3, ANC1,
84 .
85 . generate abs_mcv1dtp3 = abs(mcv1-dtp3)
   (110 missing values generated)

86 . generate abs_mcv1anc1 = abs(mcv1-anc1)
   (1,089 missing values generated)

87 . generate abs_mcv1pab = abs(mcv1-pab)
   (313 missing values generated)

88 . generate abs_dtp3anc1 = abs(dtp3-anc1)
   (1,110 missing values generated)

89 . generate abs_dtp3pab = abs(dtp3-pab)
   (334 missing values generated)

90 . generate abs_anc1pab = abs(anc1-pab)
   (1,152 missing values generated)

91 .
92 . *Set "integration" to 0 if MCV1, DTP3, ANC1, and PAB all fall below 70%.*
93 .
94 . replace integration=0 if mcv1<70 & dtp3<70 & anc1<70 & pab<70
   (0 real changes made)

95 .
96 . *Set "integration" to 1 if coverage of at least one of the four services is greater than or equ
97 .
98 . replace integration=1 if mcv1>=70 | dtp3>=70 | anc1>=70 | pab>=70
   (12,089 real changes made)

99 .
```

```
100 . *Set "integration" to 2 if co-coverage levels of at least two of the four services are greater
    > ercentage points of one another.*
101 .
102 . replace integration=2 if mcv1>=70 & dtp3>=70 & abs_mcv1dtp3<=10 | mcv1>=70 & anc1>=70 & abs_mcv
    > mcv1pab<=10 | dtp3>=70 & anc1>=70 & abs_dtp3anc1<=10 | dtp3>=70 & pab>=70 & abs_dtp3pab<=10 | a
    (10,517 real changes made)

103 .
104 . *Set "integration" to 3 if co-coverage levels of at least three of the four services are greate
    >  percentage points of one another.*
105 .
106 . replace integration=3 if mcv1>=70 & dtp3>=70 & anc1>=70 & abs_mcv1dtp3<=10 & abs_mcv1anc1<=10 &
    > 70 & pab>=70 & abs_mcv1dtp3<=10 & abs_mcv1pab<=10 & abs_dtp3pab<=10 | mcv1>=70 & anc1>=70 & pab
    > <=10 & abs_anc1pab<=10 | dtp3>=70 & anc1>=70 & pab>=70 & abs_dtp3anc1<=10 & abs_dtp3pab<=10 & a
    (7,627 real changes made)

107 .
108 . *Set "integration" to 4 if co-coverage levels of all four services are greater than or equal to
    >  of one another.*
109 .
110 . replace integration=4 if mcv1>=70 & dtp3>=70 & anc1>=70 & pab>=70 & abs_mcv1dtp3<=10 & abs_mcv1
    > anc1<=10 & abs_dtp3pab<=10 & abs_anc1pab<=10
    (2,503 real changes made)

111 .
112 . *Create a table summarizing all values of "integration."
113 .
114 . tabulate integration
```

Integrated Vaccine Delivery	Freq.	Percent	Cum.
0	**807**	**6.26**	**6.26**
1	**1,572**	**12.19**	**18.45**
2	**2,890**	**22.41**	**40.86**
3	**5,124**	**39.73**	**80.59**
4	**2,503**	**19.41**	**100.00**
Total	**12,896**	**100.00**	

```
115 .
116 . *Create a new variable ("year_dup") that duplicates the "Year" column of the dataset.*
117 .
118 . generate year_dup = year

119 .
120 .
    end of do-file

121 . do "C:\Users\sjrav\AppData\Local\Temp\STD2804_000000.tmp"

122 . *********************************************************************************************
    > ***
```

```
123 . ***Group-based trajectory analysis using socioeconomic equity ("ceqr") as the outcome of intere
    > ***
124 . ***************************************************************************************************
    > ***
125 .
126 . *Repeat the trajectory analysis using "ceqr" (socioeconomic vaccine equity) as the outcome of i
    > g variable. Re-import the dataset, perform multiple imputation, create the variable "integratio
127 .
128 . *Specify a directory for reshaped files.*
129 .
130 . cd "C:\Users\sjrav\Desktop\STATA Folder Temp"
    C:\Users\sjrav\Desktop\STATA Folder Temp

131 .
132 . *Reshape the dataset from long to wide format; wide format is required to run the traj Stata pl
133 . mi reshape wide country q5coverage q1coverage geoequity dtp3 mcv1 anc1 pab femeduc polstability
    > exthlth land lingfrac distance ceqr abs_mcv1dtp3 abs_mcv1anc1 abs_mcv1pab abs_dtp3anc1 abs_dtp3
    >  i(alpha3) j(year)

    reshaping m=0 data ...
    (note: j = 2003 2004 2005 2006 2007 2008 2009 2010 2011 2012 2013 2014 2015 2016 2017 2018 2019)

    Data                               long   ->   wide
    -----------------------------------------------------------------------------
    Number of obs.                     1326   ->      78
    Number of variables                  30   ->     477
    j variable (17 values)             year   ->   (dropped)
    xij variables:
                                    country   ->   country2003 country2004 ... country2019
                                 q5coverage   ->   q5coverage2003 q5coverage2004 ... q5coverage2019
                                 q1coverage   ->   q1coverage2003 q1coverage2004 ... q1coverage2019
                                  geoequity   ->   geoequity2003 geoequity2004 ... geoequity2019
                                       dtp3   ->   dtp32003 dtp32004 ... dtp32019
                                       mcv1   ->   mcv12003 mcv12004 ... mcv12019
                                       anc1   ->   anc12003 anc12004 ... anc12019
                                        pab   ->   pab2003 pab2004 ... pab2019
                                    femeduc   ->   femeduc2003 femeduc2004 ... femeduc2019
                               polstability   ->   polstability2003 polstability2004 ... polstability
                                  goveffect   ->   goveffect2003 goveffect2004 ... goveffect2019
                                    corrupt   ->   corrupt2003 corrupt2004 ... corrupt2019
                                        gii   ->   gii2003 gii2004 ... gii2019
                                     oopexp   ->   oopexp2003 oopexp2004 ... oopexp2019
                                     govexp   ->   govexp2003 govexp2004 ... govexp2019
                                    exthlth   ->   exthlth2003 exthlth2004 ... exthlth2019
                                       land   ->   land2003 land2004 ... land2019
                                   lingfrac   ->   lingfrac2003 lingfrac2004 ... lingfrac2019
                                   distance   ->   distance2003 distance2004 ... distance2019
                                       ceqr   ->   ceqr2003 ceqr2004 ... ceqr2019
                               abs_mcv1dtp3   ->   abs_mcv1dtp32003 abs_mcv1dtp32004 ... abs_mcv1dtp3
                               abs_mcv1anc1   ->   abs_mcv1anc12003 abs_mcv1anc12004 ... abs_mcv1anc1
                                abs_mcv1pab   ->   abs_mcv1pab2003 abs_mcv1pab2004 ... abs_mcv1pab201
                               abs_dtp3anc1   ->   abs_dtp3anc12003 abs_dtp3anc12004 ... abs_dtp3anc1
                                abs_dtp3pab   ->   abs_dtp3pab2003 abs_dtp3pab2004 ... abs_dtp3pab201
                                abs_anc1pab   ->   abs_anc1pab2003 abs_anc1pab2004 ... abs_anc1pab201
                                integration   ->   integration2003 integration2004 ... integration201
                                   year_dup   ->   year_dup2003 year_dup2004 ... year_dup2019
    -----------------------------------------------------------------------------

    reshaping m=1 data ...

    reshaping m=2 data ...

    reshaping m=3 data ...
```

```
    reshaping m=4 data ...

    reshaping m=5 data ...

    reshaping m=6 data ...

    reshaping m=7 data ...

    reshaping m=8 data ...

    reshaping m=9 data ...

    reshaping m=10 data ...

    assembling results ...

134 .
135 . *Run the plugin several times, changing only the number of groups specified ("order") and holdi
    > ect the group number that produces the highest BIC value.*
136 .
137 . traj, model(cnorm) var(ceqr*) indep(year_dup*) min(-1000) max(1000) order (0 0)

    ==== traj stata plugin ====  Jones BL  Nagin DS,  build: Mar 17 2021

    858 observations read.
    99 had no trajectory data.
    759 observations used in the trajectory model.

                             Maximum Likelihood Estimates
                             Model: Censored Normal (cnorm)

                                             Standard       T for H0:
     Group   Parameter        Estimate        Error     Parameter=0   Prob > |T|

     1       Intercept         1.30917      0.00969        135.106       0.0000

     2       Intercept         3.83211      0.04706         81.436       0.0000

             Sigma             0.45353      0.00666         68.082       0.0000

      Group membership
     1       (%)              96.70227      0.69614        138.912       0.0000
     2       (%)               3.29773      0.69614          4.737       0.0000

     BIC= -1588.45 (N=2343)  BIC= -1586.20 (N=759)  AIC= -1576.94  ll=  -1572.94

     Entropy = 0.956

138 . traj, model(cnorm) var(ceqr*) indep(year_dup*) min(-1000) max(1000) order (0 0 0)

    ==== traj stata plugin ====  Jones BL  Nagin DS,  build: Mar 17 2021

    858 observations read.
    99 had no trajectory data.
    759 observations used in the trajectory model.

                             Maximum Likelihood Estimates
                             Model: Censored Normal (cnorm)
```

Group	Parameter	Estimate	Standard Error	T for H0: Parameter=0	Prob > |T|
1	Intercept	2.12900	0.03410	62.435	0.0000
2	Intercept	1.23249	0.00908	135.703	0.0000
3	Intercept	4.30515	0.04263	100.988	0.0000
	Sigma	0.37365	0.00568	65.812	0.0000
Group membership					
1	(%)	13.59573	1.42739	9.525	0.0000
2	(%)	84.95490	1.47444	57.618	0.0000
3	(%)	1.44937	0.43429	3.337	0.0009

BIC= -1352.84 (N=2343) BIC= -1349.46 (N=759) AIC= -1335.57 ll= -1329.57

Entropy = 0.838

```
139 . traj, model(cnorm) var(ceqr*) indep(year_dup*) min(-1000) max(1000) order (0 0 0 0)
```

==== traj stata plugin ==== Jones BL Nagin DS, build: Mar 17 2021

858 observations read.
99 had no trajectory data.
759 observations used in the trajectory model.

Maximum Likelihood Estimates
Model: Censored Normal (cnorm)

Group	Parameter	Estimate	Standard Error	T for H0: Parameter=0	Prob > |T|
1	Intercept	1.19663	0.02342	51.094	0.0000
2	Intercept	1.28746	0.03072	41.908	0.0000
3	Intercept	2.13300	0.03263	65.360	0.0000
4	Intercept	4.30517	0.04214	102.163	0.0000
	Sigma	0.36920	0.00546	67.584	0.0000
Group membership					
1	(%)	50.68896	10.46004	4.846	0.0000
2	(%)	34.31097	10.79958	3.177	0.0015
3	(%)	13.55073	1.40141	9.669	0.0000
4	(%)	1.44935	0.43447	3.336	0.0009

BIC= -1359.85 (N=2343) BIC= -1355.34 (N=759) AIC= -1336.81 ll= -1328.81
Warning: variance matrix is nonsymmetric or highly singular

Entropy = 0.509

```
140 . traj, model(cnorm) var(ceqr*) indep(year_dup*) min(-1000) max(1000) order (0 0 0 0 0)

    ==== traj stata plugin ====  Jones BL  Nagin DS,  build: Mar 17 2021

    858 observations read.
    99 had no trajectory data.
    759 observations used in the trajectory model.

                          Maximum Likelihood Estimates
                          Model: Censored Normal (cnorm)

                                              Standard       T for H0:
     Group   Parameter        Estimate        Error     Parameter=0   Prob > |T|

     1       Intercept         1.18527      0.04879          24.294       0.0000

     2       Intercept         1.18527      0.01199          98.838       0.0000

     3       Intercept         1.77021      0.02483          71.297       0.0000

     4       Intercept         2.76229      0.04984          55.428       0.0000

     5       Intercept         4.30517      0.03929         109.579       0.0000

             Sigma             0.34409      0.00562          61.179       0.0000

      Group membership
     1       (%)               8.28242   4717.85935           0.002       0.9986
     2       (%)              66.68085   4717.85973           0.014       0.9887
     3       (%)              19.35626      2.05674           9.411       0.0000
     4       (%)               4.23117      0.78550           5.387       0.0000
     5       (%)               1.44929      0.43428           3.337       0.0009

     BIC= -1288.54 (N=2343)  BIC= -1282.90 (N=759)  AIC= -1259.74  ll=  -1249.74

     Entropy = 0.603

141 .
142 . *Next, toggle the polynomial order for each of the groups to achieve the largest possible BIC.
    >  option with a high BIC value, high entropy, and at least 1% membership in the smallest group.*
143 .
144 . traj, model (cnorm) var(ceqr*) indep(year_dup*) min(0) max(6) order (1 0 1) tcov(integration*)

    ==== traj stata plugin ====  Jones BL  Nagin DS,  build: Mar 17 2021

    858 observations read.
    99 had no trajectory data.
    759 observations used in the trajectory model.

    Start
    Parameter estimates

        0.72526,    0.00000,    0.00000,    1.43083,    0.00000,    2.13641,
        0.00000,    0.00000,    0.70558,   33.33333,   33.33333,   33.33333


              Neg. Log       Percent
              Likelihood     Decrease

         0        2496.3545797
         1        2326.7198866   6.79529641
         2        2217.0682264   4.71271428
         3        2180.8425208   1.63394636
         4        2156.1376261   1.13281424
         5        2142.5081573   0.63212425
```

6	2141.0240155	0.06927123
7	2138.9637912	0.09622612
8	2138.2020550	0.03561240
9	2137.6973897	0.02360232
10	2132.6779167	0.23480746
11	1916.8239385	10.12126475
12	1911.8711275	0.25838633
13	1909.2038708	0.13951027
14	1907.5034921	0.08906219
15	1905.9545348	0.08120338
16	1904.7892248	0.06114049
17	1903.4093853	0.07244053
18	1602.7668067	15.79495094
19	1445.9178209	9.78613889
20	1440.0777043	0.40390377
21	1439.9438773	0.00929304
22	1439.8448057	0.00688024
23	1407.2219214	2.26572226
24	1383.3844043	1.69394157
25	1288.3820494	6.86738658
26	1251.6872784	2.84812808
27	1247.2072585	0.35791847
28	1203.9208267	3.47066868
29	1203.9208267	-0.00000000
29	1203.9208267	-0.00000000
30	1203.9075265	0.00110474
31	1203.9016229	0.00049038
32	1203.8950577	0.00054532
33	1203.8797104	0.00127480
34	1203.8626946	0.00141341
35	1199.4920190	0.36305433
36	1193.1525833	0.52851004
37	1187.9113055	0.43927976
38	1159.0516710	2.42944354
39	1158.1252306	0.07993090
40	1158.1042271	0.00181358
41	1158.0253041	0.00681484
42	1157.9830338	0.00365020
43	1157.9756290	0.00063946
44	1157.9657096	0.00085662
45	1154.0990677	0.33391680
46	1154.0822178	0.00146000
47	1154.0497720	0.00281139
48	1154.0313867	0.00159311
49	1137.3896551	1.44205191
50	1137.3776819	0.00105269
51	1102.3514628	3.07955920
52	1064.3735198	3.44517554
53	1054.2186096	0.95407392
54	1050.4454945	0.35790633
55	1049.5757024	0.08280221
56	1049.5700406	0.00053943
57	1049.5686947	0.00012823
58	1049.5659423	0.00026224
59	1048.1135982	0.13837569
60	1047.6430916	0.04489080
61	1045.6215326	0.19296258
62	1040.8934572	0.45217847
63	1039.6614493	0.11836061
64	1038.6121811	0.10092403
65	1038.3449950	0.02572531
66	1038.1436207	0.01939378
67	1037.9627639	0.01742117
68	1037.3974298	0.05446574
69	1036.0670989	0.12823734

70	1033.7273807	0.22582690
71	1030.7875461	0.28439167
72	1028.4740911	0.22443568
73	1026.7649562	0.16618162
74	1026.3398725	0.04140029
75	1026.2966853	0.00420789
76	1026.2763892	0.00197761
77	1026.2651008	0.00109994
78	1026.2262405	0.00378657
79	1026.1581501	0.00663503
80	1026.0219239	0.01327536
81	1025.8691700	0.01488797
82	1025.7833699	0.00836365
83	1025.7668560	0.00160988
84	1025.7658262	0.00010039
85	1025.7657976	0.00000279
86	1025.7657957	0.00000018
87	1025.7657956	0.00000001
88	1025.7657956	0.00000000

Maximum Likelihood Estimates
Model: Censored Normal (cnorm)

Group	Parameter	Estimate	Standard Error	T for H0: Parameter=0	Prob > |T|
1	Intercept	11.29572	11.31957	0.998	0.3184
	Linear	-0.00417	0.00563	-0.740	0.4594
	integration2	-0.45322	0.02372	-19.105	0.0000
2	Intercept	1.73245	0.02581	67.127	0.0000
	integration2	-0.16612	0.00822	-20.213	0.0000
3	Intercept	79.75245	15.90533	5.014	0.0000
	Linear	-0.03752	0.00791	-4.744	0.0000
	integration2	-0.79609	0.05551	-14.341	0.0000
	Sigma	0.33967	0.00538	63.084	0.0000
Group membership					
1	(%)	14.50142	2.03064	7.141	0.0000
2	(%)	82.66876	2.04003	40.523	0.0000
3	(%)	2.82982	0.75038	3.771	0.0002

BIC= -1068.44 (N=2343) BIC= -1062.24 (N=759) AIC= -1036.77 ll= -1025.77

Parameter estimates for adding risk factors

11.29572,   -0.00417,   -0.45322,   1.73245,   -0.16612,   79.75245,
-0.03752,   -0.79609,   0.33967,   1.74060,   -1.63403

Parameter estimates

11.29572,   -0.00417,   -0.45322,   1.73245,   -0.16612,   79.75245,
-0.03752,   -0.79609,   0.33967,   14.50142,   82.66876,   2.82982

Entropy = 0.752

```
145 .
146 . *Plot the trajectories for the three groups, along with accompanying confidence intervals. Asse
    > es an additional diagnostic check for the model (i.e., narrower, non-overlapping CIs are prefer
147 .
148 . trajplot, xtitle("Year") ytitle("Socioeconomic Vaccine Equity Ratio") ci

149 .
    end of do-file

150 . graph export "C:\Users\sjrav\Desktop\Dissertation\Aim 3 - Quantitative Analysis\Data\STATA Outp
    > ty.png", as(png) replace
    (note: file C:\Users\sjrav\Desktop\Dissertation\Aim 3 - Quantitative Analysis\Data\STATA Output\M
    > ng not found)
    (file C:\Users\sjrav\Desktop\Dissertation\Aim 3 - Quantitative Analysis\Data\STATA Output\MI GBTM
    > tten in PNG format)

151 . do "C:\Users\sjrav\AppData\Local\Temp\STD2804_000000.tmp"

152 . matrix strt = 11.29572, -0.00417, -0.45322, 1.73245, -0.16612, 79.75245, -0.03752, -0.79609, 0.

153 .
154 . bootstrap _b (100/(1+exp(_b[theta2]))) (100*exp(_b[theta2])/(1+exp(_b[theta2]))), reps(1000) do
    > ) indep(year_dup*) min(0) max(6) order (1 0 1) tcov(integration*) detail start(strt) novar
    (running traj on estimation sample)

    Bootstrap replications (1000)
    ----+--- 1 ---+--- 2 ---+--- 3 ---+--- 4 ---+--- 5
    ..................................................   500
    ..................................................  1000

    Bootstrap results                               Number of obs     =        858
                                                    Replications      =      1,000

          command:  traj, model(cnorm) var(ceqr*) indep(year_dup*) min(0) max(6) order(1 0 1) tcov(in
                        novar
        [_eq2]_bs_1:  100/(1+exp(_b[theta2]))
        [_eq2]_bs_2:  100*exp(_b[theta2])/(1+exp(_b[theta2]))
```

	Observed Coef.	Bootstrap Std. Err.	z	P>|z|	Normal-based [95% Conf.	Interval]
**_eq1**						
interc1	**11.29572**	**323.7757**	**0.03**	**0.972**	**-623.2929**	**645.8844**
linear1	**-.0041679**	**.1611444**	**-0.03**	**0.979**	**-.3200051**	**.3116694**
integration2G1	**-.4532207**	**.326841**	**-1.39**	**0.166**	**-1.093817**	**.1873759**
interc2	**1.732449**	**.0642917**	**26.95**	**0.000**	**1.606439**	**1.858458**
integration2G2	**-.1661203**	**.0154312**	**-10.77**	**0.000**	**-.1963648**	**-.1358758**
interc3	**79.75245**	**4.545301**	**17.55**	**0.000**	**70.84382**	**88.66108**
linear3	**-.0375161**	**.0022589**	**-16.61**	**0.000**	**-.0419435**	**-.0330887**
integration2G3	**-.7960891**	**.1172229**	**-6.79**	**0.000**	**-1.025842**	**-.5663364**
sigma	**.3396704**	**.0150866**	**22.51**	**0.000**	**.3101012**	**.3692396**
theta2	**1.740595**	**.8512299**	**2.04**	**0.041**	**.0722153**	**3.408975**
theta3	**-1.634033**	**1.006986**	**-1.62**	**0.105**	**-3.60769**	**.3396234**
**_eq2**						
_bs_1	**14.92373**	**6.125558**	**2.44**	**0.015**	**2.917861**	**26.92961**
_bs_2	**85.07627**	**6.125558**	**13.89**	**0.000**	**73.07039**	**97.08214**

```
155 .
156 . estat bootstrap, percentile bc

    Bootstrap results                               Number of obs     =        858
                                                    Replications      =       1000

          command:  traj, model(cnorm) var(ceqr*) indep(year_dup*) min(0) max(6) order(1 0 1) tcov(ir
                        novar
      [_eq2]_bs_1:  100/(1+exp(_b[theta2]))
      [_eq2]_bs_2:  100*exp(_b[theta2])/(1+exp(_b[theta2]))

    ------------------------------------------------------------------------------
                 |    Observed               Bootstrap
                 |       Coef.       Bias    Std. Err.  [95% Conf. Interval]
    -------------+----------------------------------------------------------------
    _eq1         |
         interc1 |    11.29572   252.0916   323.77566   -31.58296   669.2193   (P)
                 |                                      -36.80255   666.5546  (BC)
         linear1 |  -.00416786  -.1254908   .16114442   -.3316471   .0171122   (P)
                 |                                      -.3303199   .0197288  (BC)
    integratio~1 |  -.45322069   .2804412     .326841   -.4880465   .2712846   (P)
                 |                                      -.5265808  -.3451803  (BC)
         interc2 |   1.7324487   .0408966   .06429173    1.671633   1.891477   (P)
                 |                                       1.640257   1.857885  (BC)
    integratio~2 |  -.16612027  -.0096441   .01543115    -.204167  -.1496037   (P)
                 |                                      -.1941656  -.1413382  (BC)
         interc3 |    79.75245  -1.156366   4.5453009    72.40398   82.26769   (P)
                 |                                        73.6969   82.69172  (BC)
         linear3 |   -.0375161   .0005746   .00225894   -.0387678  -.0338628   (P)
                 |                                      -.0389796  -.0345066  (BC)
    integrati~G3 |  -.79608909   .0013102   .11722293   -.8335739  -.7639533   (P)
                 |                                      -.8279467  -.7566609  (BC)
           sigma |   .33967039  -.0095594    .0150866    .3000907   .3598782   (P)
                 |                                       .3201697    .373725  (BC)
          theta2 |   1.7405953   .6596783   .85122993    1.465697   3.717476   (P)
                 |                                       1.371057   3.518992  (BC)
          theta3 |  -1.6340335   .6921629   1.0069863    -2.26368   .7319252   (P)
                 |                                      -2.560508   .4249983  (BC)
    -------------+----------------------------------------------------------------
    _eq2         |
           _bs_1 |   14.923733  -4.458555   6.1255578    2.371894   18.75975   (P)
                 |                                       2.877665   20.24492  (BC)
           _bs_2 |   85.076267   4.458555   6.1255577    81.24025   97.62811   (P)
                 |                                       79.75508   97.12234  (BC)
    ------------------------------------------------------------------------------
    (P)    percentile confidence interval
    (BC)   bias-corrected confidence interval

157 .
    end of do-file

158 . do "C:\Users\sjrav\AppData\Local\Temp\STD2804_000000.tmp"
```

```
159 . *Create a program, "trajstats," to calculate several other diagnostic criteria for group-based
160 .
161 . program trajstats
  1.
162 . preserve
  2.
163 . *This step calculates the average posterior probability.*
164 .
165 . generate Mp=0
  3.
166 . foreach i of varlist _traj_ProbG* {
  4.               replace Mp = `i' if `i' > Mp
  5.           }
  6.       sort _traj_Group
  7.
167 . by _traj_Group: generate countG = _N
  8.
168 . *This step calculates the odds of correct classification.*
169 .
170 .     by _traj_Group: egen groupAPP = mean(Mp)
  9.       by _traj_Group: generate counter = _n
 10.       generate n = groupAPP/(1 - groupAPP)
 11.       generate p = countG/ _N
 12.       generate d = p/(1-p)
 13.       generate occ = n/d
 14.
171 . *This step calculates the estimated group probabilities vs. the proportion of the sample assigr
172 .
173 .         scalar c = 0
 15.       gen TotProb = 0
 16.       foreach i of varlist _traj_ProbG* {
 17.          scalar c = c + 1
 18.          quietly summarize `i'
 19.          replace TotProb = r(sum)/ _N if _traj_Group == c
 20.       }
 21.           gen d_pp = TotProb/(1 - TotProb)
 22.           gen occ_pp = n/d_pp
 23.
174 .  *This step displays:
175 .     *Group number [_traj_~p],
176 .     *Count per group (based on the max post prob), [countG]
177 .     *Average posterior probability for each group, [groupAPP]
178 .     *Odds of correct classification (based on the maximum posterior group assignment rule), [oc
179 .     *Odds of correct classification (based on the weighted posterior probabilities), [occ_pp]
180 .     *Observed probability of groups [p] versus the probability based on the posterior probabili
181 .
182 . list _traj_Group countG groupAPP occ occ_pp p TotProb if counter == 1
 24.
183 . restore
 25.
184 . end

185 .
```

```
186 . *Now, run the trajstats program.*
187 .
188 . trajstats
    (858 real changes made)
    (754 real changes made)
    (22 real changes made)
    (82 real changes made)
    (754 real changes made)
    (22 real changes made)
```

	traj~p	countG	groupAPP	occ	occ_pp	p	TotProb
1.	1	82	.8061659	39.35882	24.52127	.0955711	.1450141
83.	2	754	.9213567	1.615952	2.456145	.8787879	.8266878
837.	3	22	.7986288	150.7062	136.1827	.025641	.0282982

```
189 .
    end of do-file

190 . do "C:\Users\sjrav\AppData\Local\Temp\STD2804_000000.tmp"

191 . *Reshape the dataset into long format.*
192 .
193 . mi reshape long country q5coverage q1coverage geoequity dtp3 mcv1 anc1 pab femeduc polstability
    > exthlth land lingfrac distance ceqr abs_mcv1dtp3 abs_mcv1anc1 abs_mcv1pab abs_dtp3anc1 abs_dtp3
    >  i(alpha3) j(year)

    reshaping m=0 data ...
    (note: j = 2003 2004 2005 2006 2007 2008 2009 2010 2011 2012 2013 2014 2015 2016 2017 2018 2019)

    Data                                   wide   ->   long
    -----------------------------------------------------------------------------

    Number of obs.                           78   ->    1326
    Number of variables                     481   ->      34
    j variable (17 values)                        ->   year
    xij variables:
    country2003 country2004 ... country2019   ->   country
    q5coverage2003 q5coverage2004 ... q5coverage2019->q5coverage
    q1coverage2003 q1coverage2004 ... q1coverage2019->q1coverage
    geoequity2003 geoequity2004 ... geoequity2019->geoequity
              dtp32003 dtp32004 ... dtp32019   ->   dtp3
              mcv12003 mcv12004 ... mcv12019   ->   mcv1
              anc12003 anc12004 ... anc12019   ->   anc1
                 pab2003 pab2004 ... pab2019   ->   pab
    femeduc2003 femeduc2004 ... femeduc2019   ->   femeduc
    polstability2003 polstability2004 ... polstability2019->polstability
    goveffect2003 goveffect2004 ... goveffect2019->goveffect
    corrupt2003 corrupt2004 ... corrupt2019   ->   corrupt
                 gii2003 gii2004 ... gii2019   ->   gii
       oopexp2003 oopexp2004 ... oopexp2019   ->   oopexp
       govexp2003 govexp2004 ... govexp2019   ->   govexp
    exthlth2003 exthlth2004 ... exthlth2019   ->   exthlth
              land2003 land2004 ... land2019   ->   land
    lingfrac2003 lingfrac2004 ... lingfrac2019->   lingfrac
    distance2003 distance2004 ... distance2019->   distance
              ceqr2003 ceqr2004 ... ceqr2019   ->   ceqr
    abs_mcv1dtp32003 abs_mcv1dtp32004 ... abs_mcv1dtp32019->abs_mcv1dtp3
    abs_mcv1anc12003 abs_mcv1anc12004 ... abs_mcv1anc12019->abs_mcv1anc1
    abs_mcv1pab2003 abs_mcv1pab2004 ... abs_mcv1pab2019->abs_mcv1pab
    abs_dtp3anc12003 abs_dtp3anc12004 ... abs_dtp3anc12019->abs_dtp3anc1
    abs_dtp3pab2003 abs_dtp3pab2004 ... abs_dtp3pab2019->abs_dtp3pab
    abs_anc1pab2003 abs_anc1pab2004 ... abs_anc1pab2019->abs_anc1pab
    integration2003 integration2004 ... integration2019->integration
```

```
year_dup2003 year_dup2004 ... year_dup2019->   year_dup
--------------------------------------------------------------------------------

reshaping m=1 data ...

reshaping m=2 data ...

reshaping m=3 data ...

reshaping m=4 data ...

reshaping m=5 data ...

reshaping m=6 data ...

reshaping m=7 data ...

reshaping m=8 data ...

reshaping m=9 data ...

reshaping m=10 data ...

assembling results ...

194 .
195 . *Perform multinomial logistic regression to identify covariates with statistically significant
    > etting Group 1 membership as the base outcome.*
196 .
197 . mlogit _traj_Group femeduc polstability goveffect corrupt gii oopexp govexp exthlth land lingfr
    > 1)

Iteration 0:   log likelihood = -1388.6768
Iteration 1:   log likelihood = -1168.0126
Iteration 2:   log likelihood = -869.64423
Iteration 3:   log likelihood = -795.47526
Iteration 4:   log likelihood = -751.75884
Iteration 5:   log likelihood = -738.19827
Iteration 6:   log likelihood = -734.22841
Iteration 7:   log likelihood = -733.11051
Iteration 8:   log likelihood = -732.82523
Iteration 9:   log likelihood = -732.76152
Iteration 10:  log likelihood = -732.74799
Iteration 11:  log likelihood = -732.74486
Iteration 12:  log likelihood =  -732.7441
Iteration 13:  log likelihood = -732.74395
Iteration 14:  log likelihood = -732.74392

Multinomial logistic regression                 Number of obs     =      3,286
                                                LR chi2(24)       =    1311.87
                                                Prob > chi2       =     0.0000
Log likelihood = -732.74392                     Pseudo R2         =     0.4723
```

_traj_Group	Coef.	Std. Err.	z	P>|z|	[95% Conf.	Interval]
**1**	(base outcome)					
**2**						
femeduc	.0335643	.0057491	5.84	0.000	.0222963	.0448324
polstability	.2190126	.1167022	1.88	0.061	-.0097195	.4477448
goveffect	-1.352827	.3111789	-4.35	0.000	-1.962727	-.7429278
corrupt	-.143716	.3211633	-0.45	0.655	-.7731845	.4857525
gii	1.282163	.3894425	3.29	0.001	.5188694	2.045456
oopexp	-.0165694	.0051206	-3.24	0.001	-.0266055	-.0065333

govexp	.014214	.002298	6.19	0.000	.00971	.0187181
exthlth	.0333253	.0082424	4.04	0.000	.0171705	.04948
land	-9.34e-07	1.28e-07	-7.33	0.000	-1.18e-06	-6.84e-07
lingfrac	1.529981	.38087	4.02	0.000	.7834891	2.276472
distance	.0059308	.0049337	1.20	0.229	-.0037391	.0156007
integration	.6124119	.0791537	7.74	0.000	.4572735	.7675504
_cons	-4.40065	.672575	-6.54	0.000	-5.718873	-3.082428
**3**						
femeduc	-.7187821	158.7327	-0.00	0.996	-311.8291	310.3915
polstability	5.386423	7081.18	0.00	0.999	-13873.47	13884.25
goveffect	-4.53724	9674.134	-0.00	1.000	-18965.49	18956.42
corrupt	-11.25889	6490.384	-0.00	0.999	-12732.18	12709.66
gii	52.10258	9972.683	0.01	0.996	-19494	19598.2
oopexp	-.8452196	185.0853	-0.00	0.996	-363.6058	361.9153
govexp	.0817501	9.445754	0.01	0.993	-18.43159	18.59509
exthlth	-1.743924	581.1434	-0.00	0.998	-1140.764	1137.276
land	.0000257	.0069168	0.00	0.997	-.013531	.0135823
lingfrac	87.24992	31676.4	0.00	0.998	-61997.36	62171.86
distance	-2.383606	131.6725	-0.02	0.986	-260.457	255.6898
integration	-17.87322	3817.841	-0.00	0.996	-7500.704	7464.957
_cons	69.14296	18927.01	0.00	0.997	-37027.11	37165.4

Note: 154 observations completely determined. Standard errors questionable.

```
198 .
    end of do-file

199 .
```
